# Supplementary material for: A diabetic milieu increases ACE2 expression and cellular susceptibility to SARS-CoV-2 infections in human kidney organoids and patient cells
Source: Cell Metab. 2022 Jun 7;34(6):857–873.e9. doi: 10.1016/j.cmet.2022.04.009 (PMC9097013; doi:10.1016/j.cmet.2022.04.009)
Supplement: Document S1. Figures S1–S9 [file mmc1.pdf]

Cell Metabolism, Volume *34*

Supplemental information

A diabetic milieu increases ACE2 expression and cellular susceptibility to SARS-CoV-2 infections in human kidney organoids and patient cells

Elena Garreta, Patricia Prado, Megan L. Stanifer, Vanessa Monteil, Andrés Marco, Asier Ullate-Agote, Daniel Moya-Rull, Amaia Vilas-Zornoza, Carolina Tarantino, Juan Pablo Romero, Gustav Jonsson, Roger Oria, Alexandra Leopoldi, Astrid Hagelkruys, Maria Gallo, Federico González, Pere Domingo-Pedrol, Aleix Gavaldà, Carmen Hurtado del Pozo, Omar Hasan Ali, Pedro Ventura-Aguiar, Josep María Campistol, Felipe Prosper, Ali Mirazimi, Steeve Boulant, Josef M. Penninger, and Nuria Montserrat

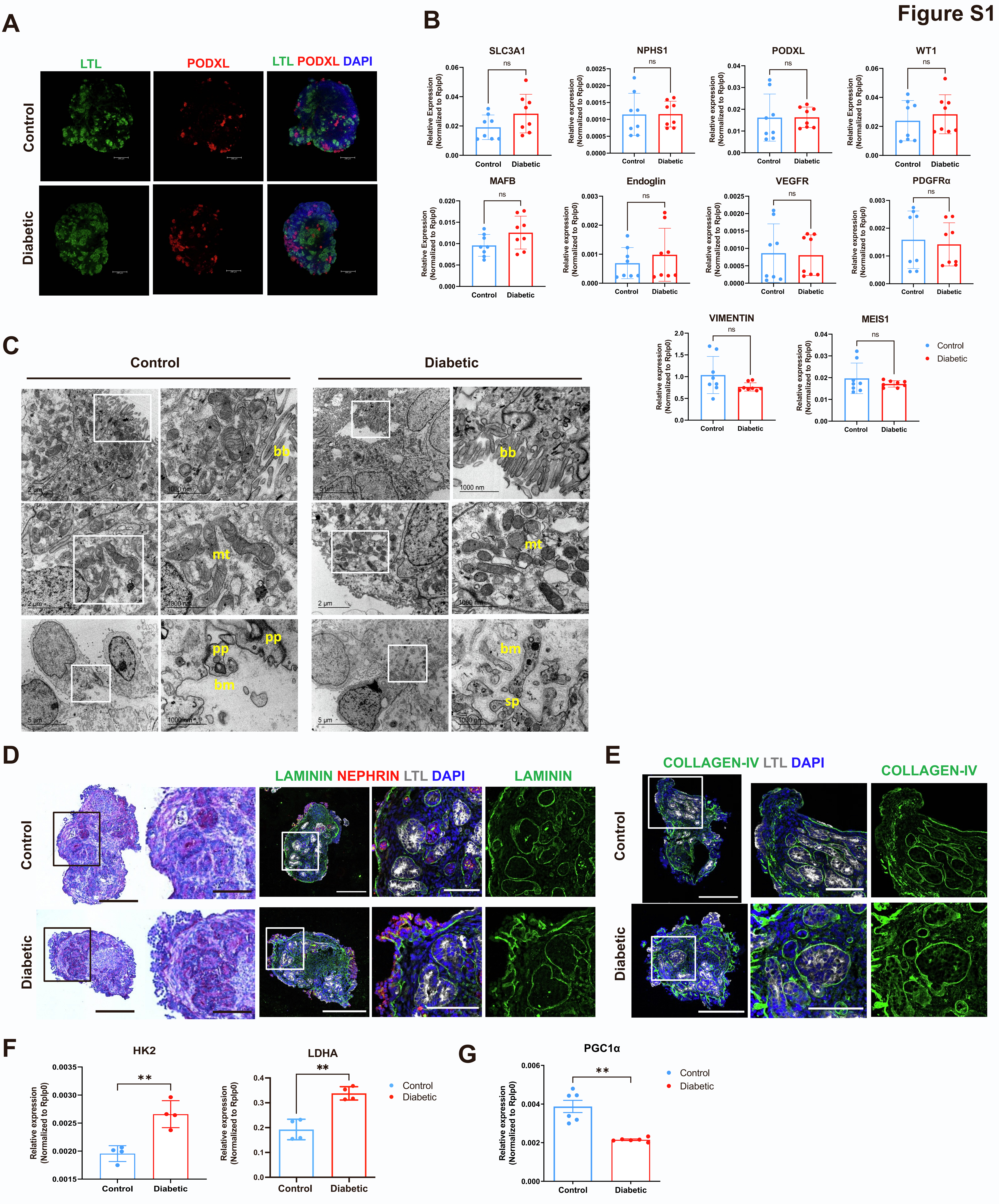

Figure S1
A
LTL
PODXL
LTL PODXL DAPI
Control
Diabetic
B
SLC3A1
NPHS1
PODXL
WT1
MAFB
Endoglin
VEGFR
PDGFRα
VIMENTIN
MEIS1
ns
Relative Expression (Normalized to Rplp0)
Relative expression (Normalized to Rplp0)
Control
Diabetic
C
Control
Diabetic
bb
mt
pp
bm
sp
D
LAMININ NEPHRIN LTL DAPI
LAMININ
Control
Diabetic
E
COLLAGEN-IV LTL DAPI
COLLAGEN-IV
Control
Diabetic
F
HK2
LDHA
**
Relative expression (Normalized to Rplp0)
Control
Diabetic
G
PGC1α
**
Relative expression (Normalized to Rplp0)
Control
Diabetic

Figure S1. High Oscillatory Glucose Regime Promotes Diabetogenic-like Responses in Human Kidney Organoids, Related to Figure 1.

A) Representative immunofluorescence staining of LTL (green), Podocalyxin (PODXL; red) and DAPI (blue) in kidney organoids cultured under Control or Diabetic conditions for 7 days. Scale bars, 250 µm.

B) mRNA expression level of proximal tubule markers (*SLC3A1*, podocyte markers (*NPHS1*, *PODXL*, *WT1*, *MAFB*), endothelial-like markers (*Endoglin*, *VEGFR*, *PDGFR*α) and stromal-like markers (*MEIS* and *VIMENTIN*) in Control and Diabetic kidney organoids. The data are represented as mean ± s.d. n = 4 independent biological replicates from a pool of 12 organoids/group with at least two technical replicates each; ns, no statistical significance, unpaired Student's *t*-test.

C) TEM in kidney organoids cultured in Control or Diabetic conditions for 7 days. Magnified views of the boxed regions show details of tubular-like epithelial cell-related structures including brush borders (bb) and high mitochondrial (mit) content (upper and middle panels). Bottom panels show podocyte-related structures, including the deposition of a basement membrane (bm), and primary (pp) and secondary cell processes (sp). Scale bars, 2 µm and 5 µm; 1000 nm (magnified views).

D) Representative Periodic Acid-Schiff (PAS) staining in Control and Diabetic kidney organoids. Scale bars, 250 µm, 100 µm (magnified views). Consecutive sections were stained for LAMININ (green), NEPHRIN (red), LTL (grey) and DAPI (blue). Scale bars, 250 µm, 100 µm (magnified views).

E) Representative immunofluorescence staining of COLLAGEN-IV (green) LTL (grey) and DAPI (blue) in Control and Diabetic kidney organoids. Scale bars, 250 µm, 100 µm (magnified views).

F) mRNA expression level of glycolytic enzymes (*HK2* and *LDHA*) in Control and Diabetic kidney organoids. The data are represented as mean ± s.d. n = 2 independent biological replicates per condition with at least two technical replicates each. $^{**}P < 0.01$, unpaired Student's *t*-test.

G) mRNA expression level of *PGC1*α in Control and Diabetic kidney organoids. The data are represented as mean ± s.d. n = 3 independent biological replicates per condition with at least two technical replicates each. ns, no statistical significance, $^{**}P < 0.01$, unpaired Student's *t*-test.

Figure S2

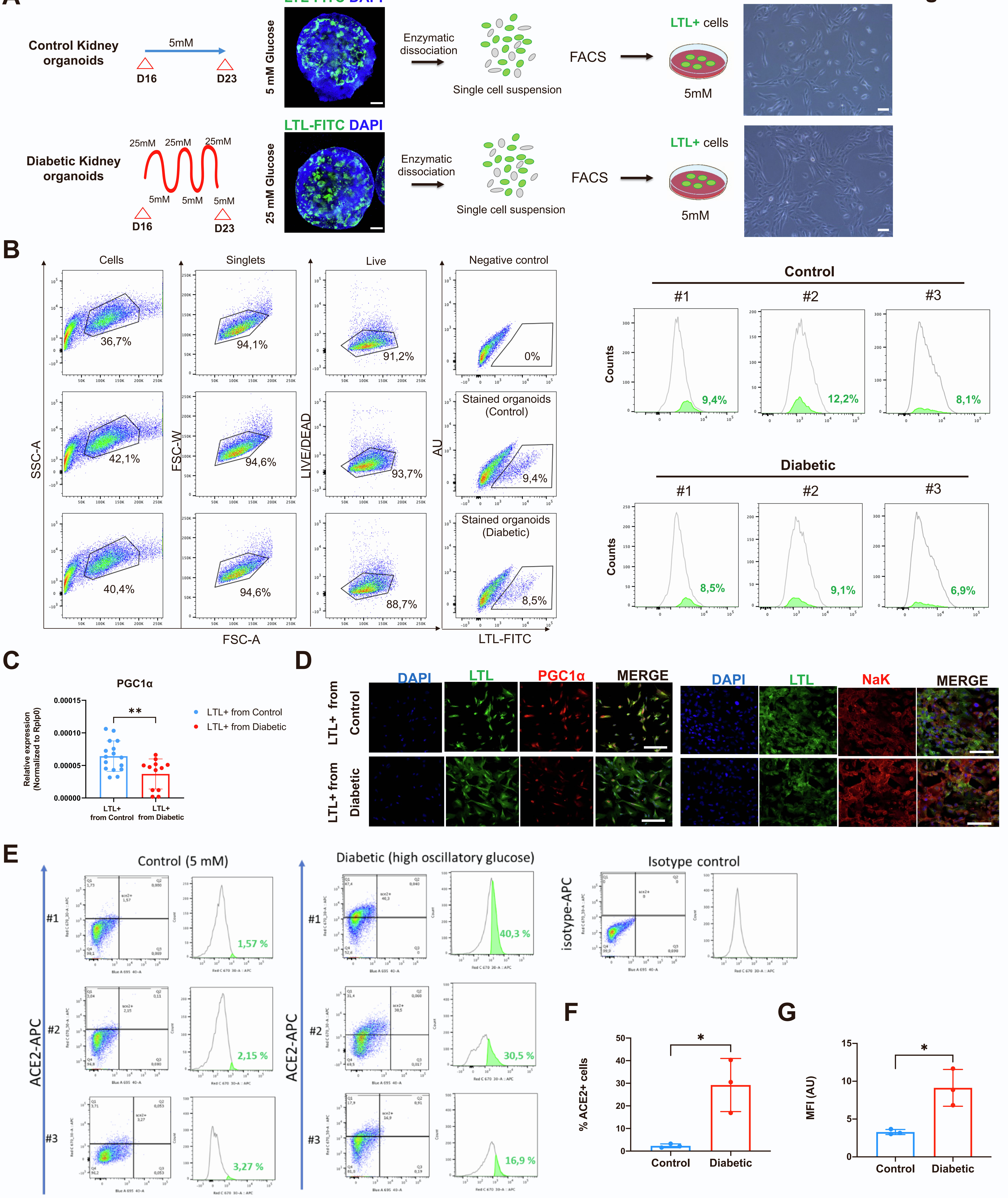
A
Control Kidney organoids
5mM
D16
D23
Diabetic Kidney organoids
25mM 25mM 25mM
5mM 5mM 5mM
LTL-FITC DAPI
5 mM Glucose
25 mM Glucose
Enzymatic dissociation
Single cell suspension
FACS
LTL+ cells
5mM
B
Cells
Singlets
Live
Negative control
Stained organoids (Control)
Stained organoids (Diabetic)
SSC-A
FSC-W
LIVE/DEAD
AU
FSC-A
LTL-FITC
36,7%
94,1%
91,2%
0%
42,1%
94,6%
93,7%
9,4%
40,4%
94,6%
88,7%
8,5%
Control
#1 #2 #3
Counts
9,4%
12,2%
8,1%
Diabetic
8,5%
9,1%
6,9%
C
PGC1α
Relative expression (Normalized to Rplp0)
0.00015
0.00010
0.00005
0.00000
**
LTL+ from Control
LTL+ from Diabetic
D
DAPI LTL PGC1α MERGE
DAPI LTL NaK MERGE
LTL+ from Control
LTL+ from Diabetic
E
Control (5 mM)
Diabetic (high oscillatory glucose)
Isotype control
ACE2-APC
isotype-APC
#1 #2 #3
1,57 %
2,15 %
3,27 %
40,3 %
30,5 %
16,9 %
F
% ACE2+ cells
50 40 30 20 10 0
*
Control Diabetic
G
MFI (AU)
15 10 5 0
*
Control Diabetic

Figure S2. Characterization of Tubular Epithelial Cells Derived from Diabetic Human Kidney Organoids Reveals Alterations in Cellular Metabolism, Related to Figure 1 and Figure 2.

A) Kidney organoids exposed to Control or Diabetic conditions were stained with LTL-FITC and dissociated to single cells. Proximal-like epithelial cells (LTL+ cells) were isolated by cell sorting for LTL marker expression and further cultured in 5 mM glucose culture medium. Right panels show representative bright field images. Scale bars, 100 μm.

B) Gating strategy for cell sorting of LTL+ cells from kidney organoids exposed to Control or Diabetic conditions. Numbers in outlined areas indicate percent cells. AU, autofluorescence. Histograms representing the overlay of live cells and LTL+ cells isolated from Control and Diabetic kidney organoids from 3 independent cell sorting experiments.

C) mRNA expression levels of *PGC1α* in LTL+ cells isolated from kidney organoids exposed to Control or Diabetic conditions. The data are presented as mean ± s.d. of at least n = 6 independent biological replicates per condition with two technical replicates each. **$P < 0.01$, unpaired Student's *t*-test.

D) Representative immunofluorescence staining in LTL+ cells isolated from Control or Diabetic kidney organoids and subsequently expanded in 5 mM glucose culture medium for the expression of proximal tubular cell markers including LTL (green), PGC1α (red) and Na-K ATPase (NaK; red). Nuclei were counterstained with DAPI (blue). Scale bars, 100 μm.

E) ACE2 expression in kidney organoids cultured under control or diabetic-like conditions.

F) Representative FACS plots of kidney organoid cells after exposure to control or diabetic-like conditions. Data from 3 independent experiments.

G) Correspondent quantification of the percentage of $ACE2^+$ cells in Control and Diabetic organoids in F). Data are mean ± SD of n = 3 independent experiments, unpaired Student's *t*-test.

H) Mean fluorescence intensity of ACE2+ cells in Control or Diabetic conditions. Data are mean ± SD of n = 3 independent experiments, unpaired Student's *t*-test.

Figure S3

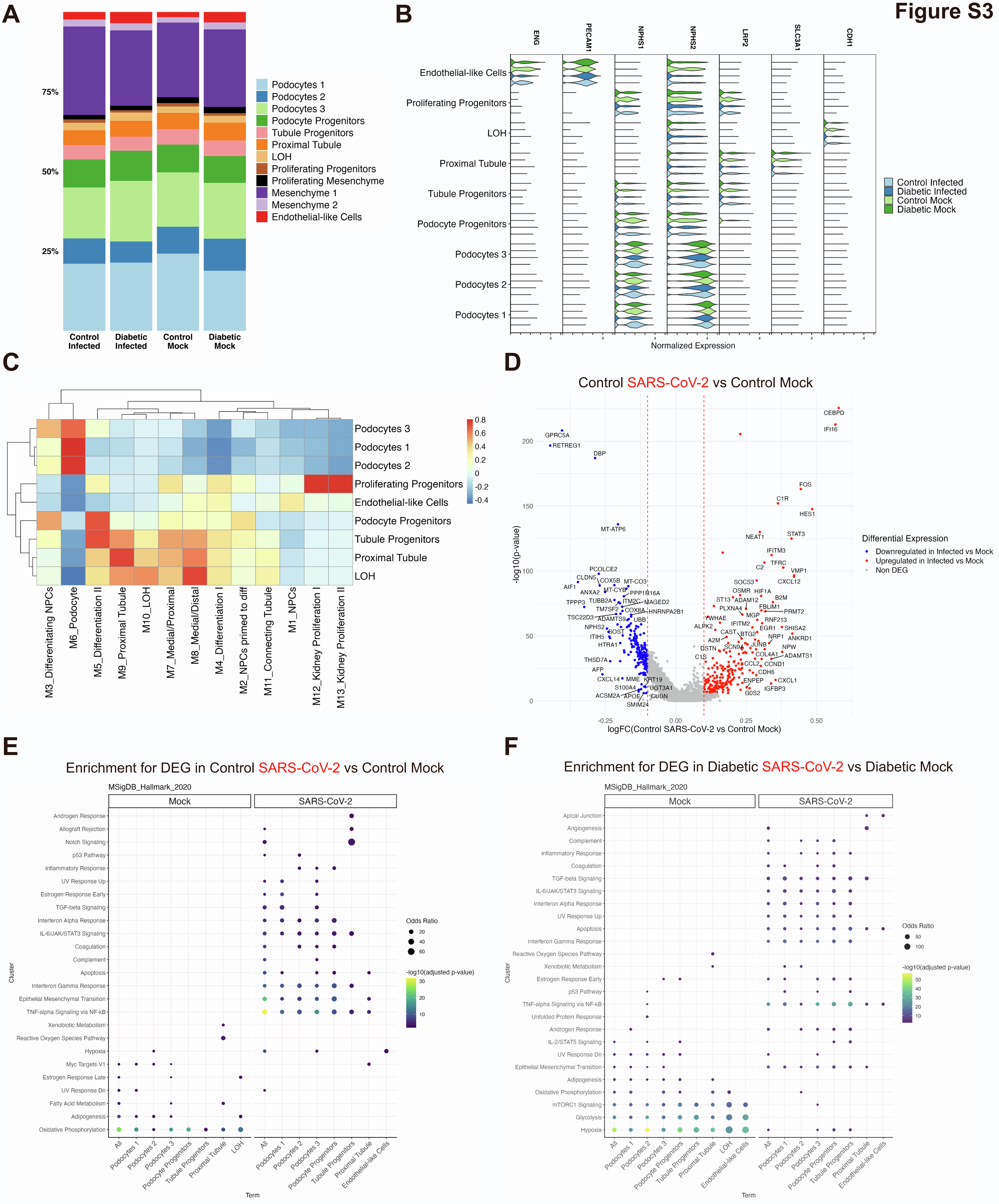

A
B
C
D
E
F
75%
50%
25%
Control Infected
Diabetic Infected
Control Mock
Diabetic Mock
Podocytes 1
Podocytes 2
Podocytes 3
Podocyte Progenitors
Tubule Progenitors
Proximal Tubule
LOH
Proliferating Progenitors
Proliferating Mesenchyme
Mesenchyme 1
Mesenchyme 2
Endothelial-like Cells
ENG
PECAM1
NPHS1
NPHS2
LRP2
SLC3A1
CDH1
Normalized Expression
M3_Differenitating NPCs
M6_Podocyte
M5_Differentiation II
M9_Proximal Tubule
M10_LOH
M7_Medial/Proximal
M8_Medial/Distal
M4_Differentiation I
M2_NPCs primed to diff
M11_Connecting Tubule
M1_NPCs
M12_Kidney Proliferation I
M13_Kidney Proliferation II
Control SARS-CoV-2 vs Control Mock
-log10(p-value)
logFC(Control SARS-CoV-2 vs Control Mock)
Differential Expression
Downregulated in Infected vs Mock
Upregulated in Infected vs Mock
Non DEG
Enrichment for DEG in Control SARS-CoV-2 vs Control Mock
Enrichment for DEG in Diabetic SARS-CoV-2 vs Diabetic Mock
MSigDB_Hallmark_2020
Mock
SARS-CoV-2
Cluster
Term
Odds Ratio
-log10(adjusted p-value)

Figure S3. Phenotypic and Transcriptional Changes in Diabetic Human Kidney Organoids after SARS-CoV-2 Infection, Related to Figure 3.

A) Cell type proportions in Mock or SARS-CoV-2 infected (10^6 virus particles/organoid as determined in Vero cells) kidney organoids exposed to Control or Diabetic conditions after discarding non-renal cells and Proliferating Progenitors 2 clusters.

B) Violin plots for the indicated markers in Mock or SARS-CoV-2 infected (10^6 virus particles/organoid as determined in Vero cells) kidney organoids exposed to Control or Diabetic conditions.

C) Correlation analysis between scores for gene modules from second semester fetal-kidney cell types [1] and scores for gene signatures from the annotated renal-like cell types in Mock and SARS-CoV-2 infected kidney organoids. Scores were obtained with the *AddModuleScore* function from Seurat. Color scale denotes Pearson correlation coefficients.

D) Differentially expressed genes (DEGs) in the comparison of SARS-CoV-2 infected (10^6 virus particles/organoid as determined in Vero cells) against Mock kidney organoids in Control conditions considering only renal-like cell types (Podocytes 1/2/3, PT, LOH, Tubule Progenitors, Podocyte Progenitors, Proliferating Progenitors and Endothelial-like Cells). In the volcano plot, the x-axis indicates log fold change (FC) and the y-axis indicates statistical significance with the –log10(p-value). Genes with an adjusted p-value < 0.05 are considered upregulated (red) if the logFC > 0.1 and downregulated (blue) if the logFC < -0.1. Non DEG are shown in grey.

E) Gene over-representation analysis using the Hallmark database considering DEGs (adjusted p-value < 0.05 and logFC > 0.1) to compare Mock and SARS-CoV-2 infected (10^6 virus particles/organoid as determined in Vero cells) kidney organoids cultured in Control conditions. Each column corresponds to the analysis of DEG upregulated in each condition. Only the ten gene sets with lowest adjusted p-value in each comparison are shown. Circles are coded by color (p-value) and size (Odds ratio).

F) Gene over-representation analysis using the Hallmark database considering DEGs (adjusted p-value < 0.05 and logFC > 0.1) to compare Mock and SARS-CoV-2 infected (10^6 virus particles/organoid as determined in Vero cells) kidney organoids cultured in Diabetic conditions. Each column corresponds to the analysis of DEG upregulated in each condition. Only the ten gene sets with lowest adjusted p-value in each comparison are shown. Circles are coded by color (p-value) and size (Odds ratio).

Figure S4

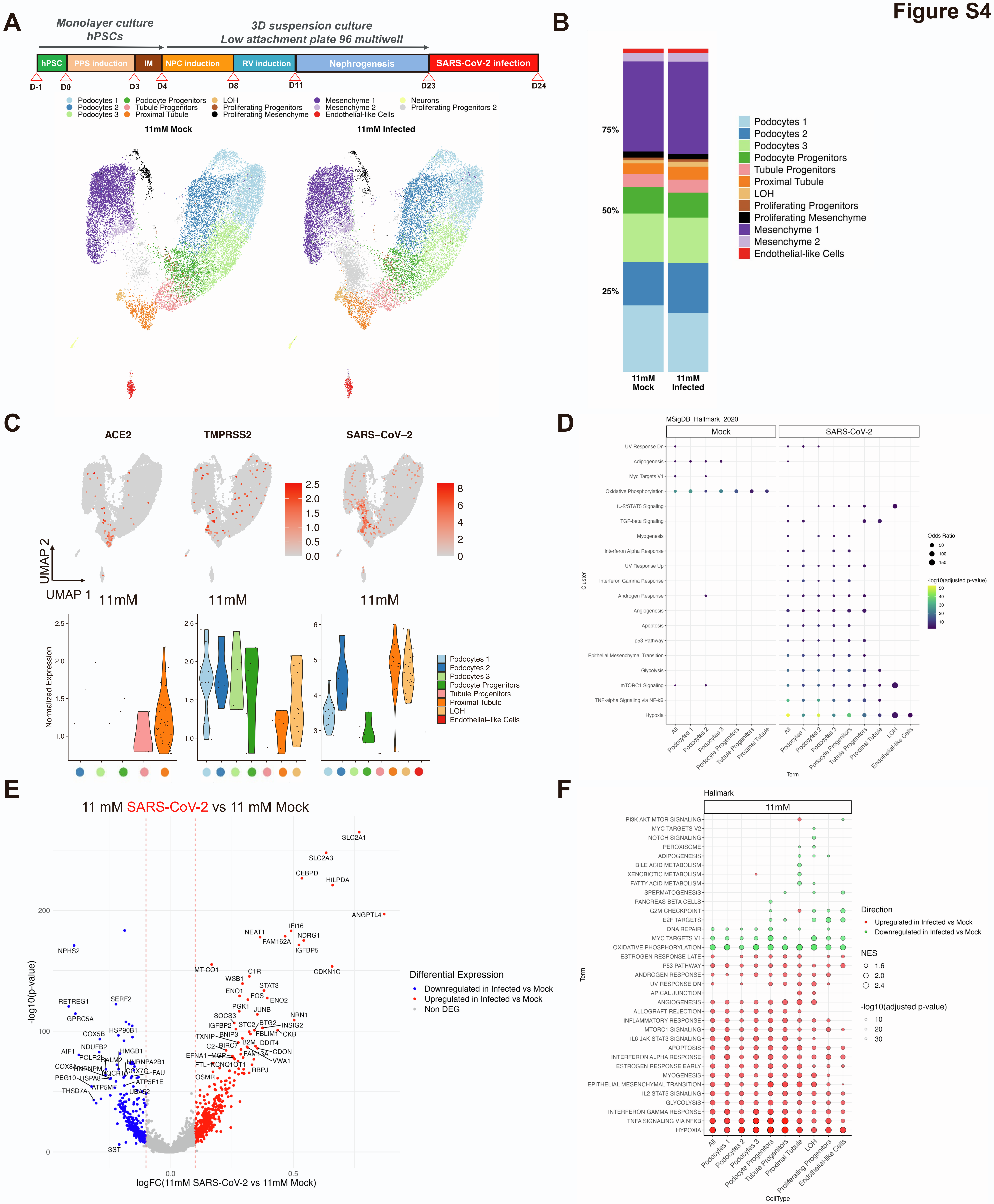

A
Monolayer culture hPSCs
3D suspension culture
Low attachment plate 96 multiwell
hPSC
PPS induction
IM
NPC induction
RV induction
Nephrogenesis
SARS-CoV-2 infection
D-1
D0
D3
D4
D8
D11
D23
D24
Podocytes 1
Podocytes 2
Podocytes 3
Podocyte Progenitors
Tubule Progenitors
Proximal Tubule
LOH
Proliferating Progenitors
Proliferating Mesenchyme
Mesenchyme 1
Mesenchyme 2
Endothelial-like Cells
Neurons
Proliferating Progenitors 2
11mM Mock
11mM Infected
B
75%
50%
25%
11mM Mock
11mM Infected
Podocytes 1
Podocytes 2
Podocytes 3
Podocyte Progenitors
Tubule Progenitors
Proximal Tubule
LOH
Proliferating Progenitors
Proliferating Mesenchyme
Mesenchyme 1
Mesenchyme 2
Endothelial-like Cells
C
ACE2
TMPRSS2
SARS–CoV–2
UMAP 2
UMAP 1
11mM
11mM
11mM
Normalized Expression
Podocytes 1
Podocytes 2
Podocytes 3
Podocyte Progenitors
Tubule Progenitors
Proximal Tubule
LOH
Endothelial–like Cells
D
MSigDB_Hallmark_2020
Mock
SARS-CoV-2
UV Response Dn
Adipogenesis
Myc Targets V1
Oxidative Phosphorylation
IL-2/STAT5 Signaling
TGF-beta Signaling
Myogenesis
Interferon Alpha Response
UV Response Up
Interferon Gamma Response
Androgen Response
Angiogenesis
Apoptosis
p53 Pathway
Epithelial Mesenchymal Transition
Glycolysis
mTORC1 Signaling
TNF-alpha Signaling via NF-kB
Hypoxia
Cluster
Term
Odds Ratio
-log10(adjusted p-value)
E
11 mM SARS-CoV-2 vs 11 mM Mock
-log10(p-value)
logFC(11mM SARS-CoV-2 vs 11mM Mock)
Differential Expression
Downregulated in Infected vs Mock
Upregulated in Infected vs Mock
Non DEG
SLC2A1
SLC2A3
CEBPD
HILPDA
ANGPTL4
IFI16
NEAT1
NDRG1
FAM162A
IGFBP5
CDKN1C
MT-CO1
C1R
NPHS2
RETREG1
SERF2
GPRC5A
HSP90B1
COX5B
NDUFB2
HMGB1
AIF1
SST
F
Hallmark
11mM
PI3K AKT MTOR SIGNALING
MYC TARGETS V2
NOTCH SIGNALING
PEROXISOME
ADIPOGENESIS
BILE ACID METABOLISM
XENOBIOTIC METABOLISM
FATTY ACID METABOLISM
SPERMATOGENESIS
PANCREAS BETA CELLS
G2M CHECKPOINT
E2F TARGETS
DNA REPAIR
MYC TARGETS V1
OXIDATIVE PHOSPHORYLATION
ESTROGEN RESPONSE LATE
P53 PATHWAY
ANDROGEN RESPONSE
UV RESPONSE DN
APICAL JUNCTION
ANGIOGENESIS
ALLOGRAFT REJECTION
INFLAMMATORY RESPONSE
MTORC1 SIGNALING
IL6 JAK STAT3 SIGNALING
APOPTOSIS
INTERFERON ALPHA RESPONSE
ESTROGEN RESPONSE EARLY
MYOGENESIS
EPITHELIAL MESENCHYMAL TRANSITION
IL2 STAT5 SIGNALING
GLYCOLYSIS
INTERFERON GAMMA RESPONSE
TNFA SIGNALING VIA NFKB
HYPOXIA
Term
CellType
Direction
Upregulated in Infected vs Mock
Downregulated in Infected vs Mock
NES
-log10(adjusted p-value)

Figure S4. Single Cell RNA Sequencing Analysis of SARS-CoV-2 Infected Human Kidney Organoids exposed to 11 mM Glucose Conditions, Related to Figure 3.

A) Schematics for the infection of kidney organoids under 11 mM glucose conditions. Uniform manifold approximation and projection (UMAP) of kidney organoids at 1 dpi with SARS-CoV-2 (10^6 virus particles/organoid as determined in Vero cells). Clusters are colored by cell type annotation.

B) Cell type proportions in the indicated kidney organoid samples after discarding non-renal cells and Proliferating Progenitors 2 clusters.

C) UMAPs for *ACE2, TMPRSS2* and SARS-CoV-2 mRNA expression in kidney organoids exposed to 11 mM glucose at 1dpi. For SARS-CoV-2, expression is considered as undetectable for cells expressing < 5 UMIs. Cells are color-coded based on the expression levels. The violin plots in the bottom panels represent expression level in cells expressing the gene of interest (*ACE2* or *TMPRSS2*) and in cells expressing ≥ 5 UMIs for SARS-CoV-2 mRNA for the indicated cell types.

D) Gene over-representation analysis using the Hallmark database considering DEGs (adjusted p-value < 0.05 and logFC > 0.1) to compare Mock and SARS-CoV-2 infected kidney organoids (10^6 virus particles/organoid as determined in Vero cells) cultured in 11 mM glucose. Each column corresponds to the analysis of DEG upregulated in each condition. Only the ten gene sets with lowest adjusted p-value in each comparison are shown. Circles are coded by color (p-value) and size (Odds ratio).

E) Differentially expressed genes (DEGs) in SARS-CoV-2 infected (10^6 virus particles/organoid as determined in Vero cells) against Mock samples in 11 mM glucose considering only renal-like cell types (Podocytes 1/2/3, PT, LOH, Tubule Progenitors, Podocyte Progenitors, Proliferating Progenitors y Endothelial-like cells). In the volcano plot, the x-axis indicates log fold change (FC) and the y-axis indicates statistical significance with the –log10(p-value Genes with an adjusted p-value < 0.05 are considered upregulated (red) if the logFC > 0.1 and downregulated (blue) if the logFC < -0.1. Non DEG are shown in grey.

F) A hallmark gene set enrichment analysis (GSEA) was performed for kidney organoids cultured in 11 mM glucose comparing SARS-CoV-2 infected (10^6 virus particles/organoid as determined in Vero cells) versus Mock samples. The ten gene sets per direction and sample with lowest adjusted p-value are shown. Each column corresponds to one of the comparisons. Circles are coded by color (direction), size (NES) and transparency (p-value).

Figure S5

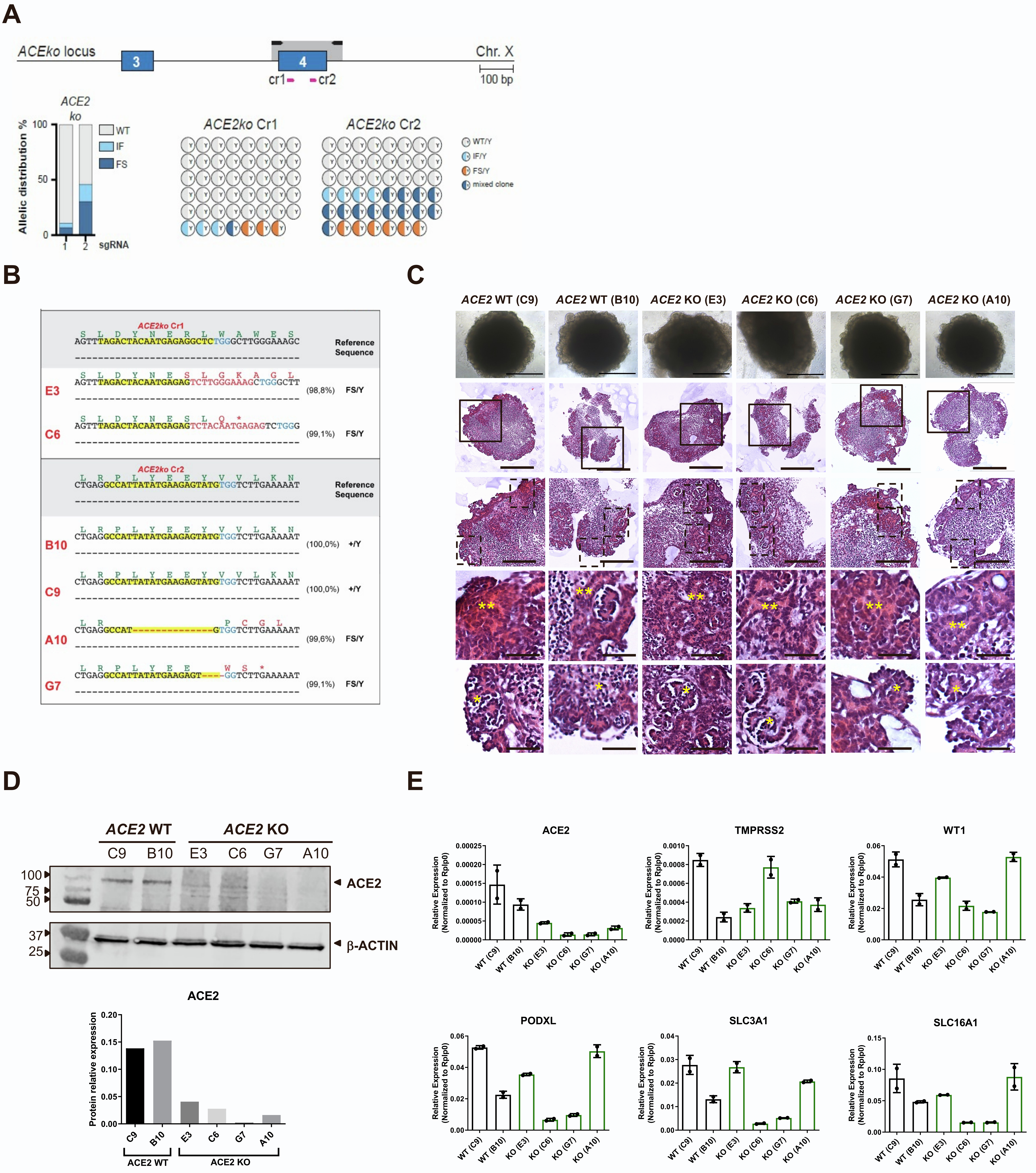
A
ACEko locus
3
4
cr1
cr2
Chr. X
100 bp
ACE2 ko
Allelic distribution %
100
50
0
1
2
sgRNA
WT
IF
FS
ACE2ko Cr1
ACE2ko Cr2
WT/Y
IF/Y
FS/Y
mixed clone
B
ACE2ko Cr1
Reference Sequence
E3
(98,8%)
FS/Y
C6
(99,1%)
FS/Y
ACE2ko Cr2
Reference Sequence
B10
(100,0%)
+/Y
C9
(100,0%)
+/Y
A10
(99,6%)
FS/Y
G7
(99,1%)
FS/Y
C
ACE2 WT (C9)
ACE2 WT (B10)
ACE2 KO (E3)
ACE2 KO (C6)
ACE2 KO (G7)
ACE2 KO (A10)
D
ACE2 WT
ACE2 KO
C9
B10
E3
C6
G7
A10
100
75
50
37
25
ACE2
β-ACTIN
ACE2
Protein relative expression
0.20
0.15
0.10
0.05
0.00
ACE2 WT
ACE2 KO
E
ACE2
TMPRSS2
WT1
PODXL
SLC3A1
SLC16A1
Relative Expression (Normalized to Rplp0)
WT (C9)
WT (B10)
KO (E3)
KO (C6)
KO (G7)
KO (A10)

Figure S5. Generation and Infection of *ACE2* KO Human Kidney Organoids, Related to Figure 4.

A) Schematic of Cas9/gRNA-targeting sites (pink arrows) in *ACE2 locus* showing exon structure (blue boxes) and PCR amplicons (light gray boxes in all figures in this study). Histogram shows allelic sequence distribution after the transfection of the different gRNAs in undifferentiated human pluripotent stem cells expressing an inducible Cas9 (iCas9). wt, wild-type; mut, mutation; FS, frameshift.

B) Representative sequence of the wild type (+Y) or *ACE2* mutant clones generated with the different gRNAs.

C) Representative phase contrast images of *ACE2* WT and *ACE2* KO kidney organoids. Scale bars, 250 µm. Same specimens were analyzed by Hematoxylin and Eosin staining showing tubular-like (**) and glomerular-like (*) structures. Scale bars, Scale bars, 250 µm, 100 µm (magnified views); 50 µm (magnified views from dashed line boxes).

D) Protein levels of ACE2 in the generated clones as determined by Western Blot. β-ACTIN was used as loading control. Quantification of changes in ACE2 protein expression is shown. The data are represented as mean ± s.d. *n* = 1 independent experiment from a pool of 12 organoids/group.

E) mRNA expression level of *ACE2* and *TMPRSS2*, podocyte markers (*WT1* and *PODXL*) and proximal tubular markers (*SLC3A1* and *SLC16A1*) in *ACE2* WT and *ACE2* KO kidney organoids. The data are represented as mean ± s.d. *n* = 1 independent experiment from a pool of 12 organoids/group with at least two technical replicates each.

Figure S6

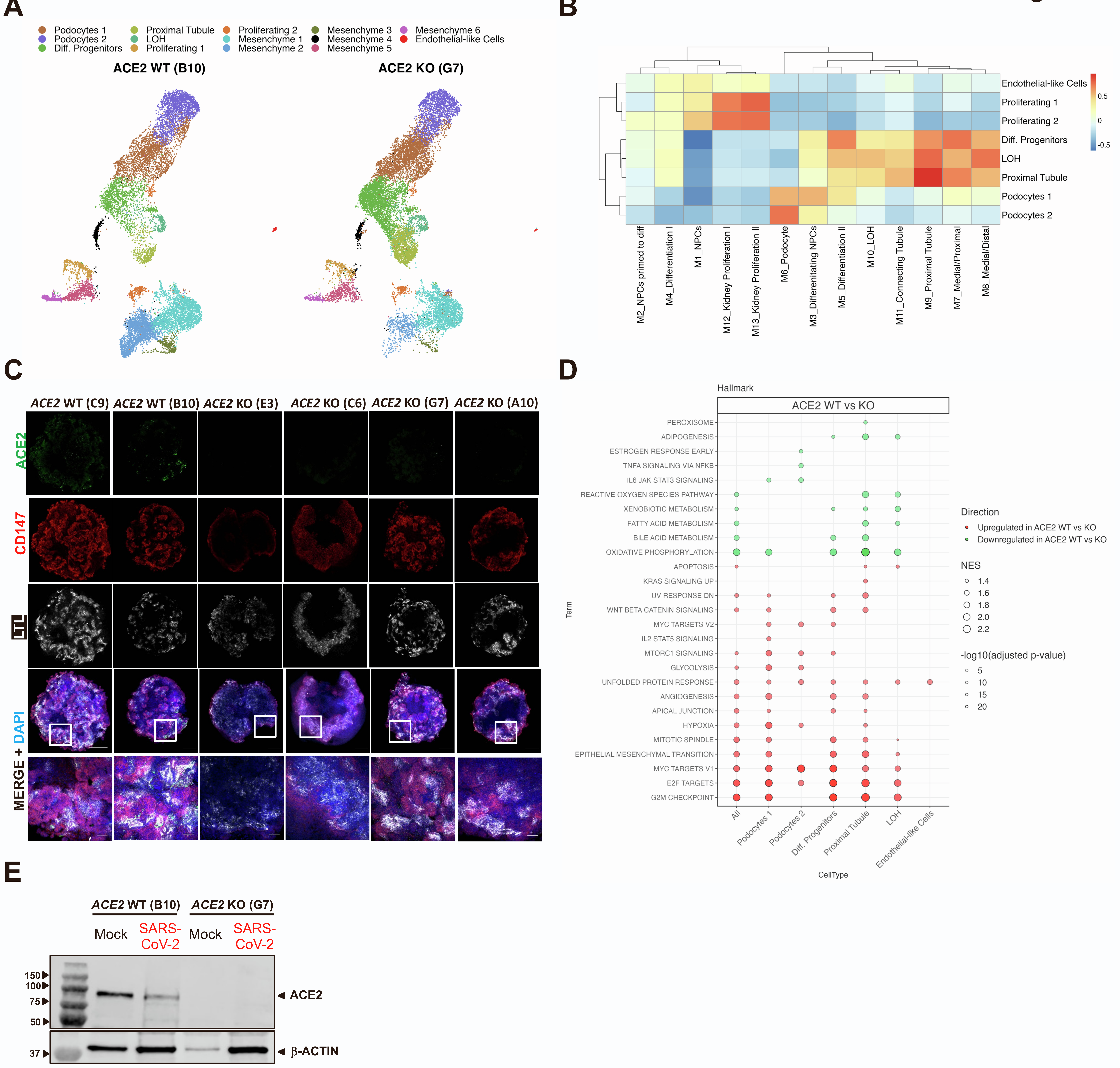
A
Podocytes 1
Podocytes 2
Diff. Progenitors
Proximal Tubule
LOH
Proliferating 1
Proliferating 2
Mesenchyme 1
Mesenchyme 2
Mesenchyme 3
Mesenchyme 4
Mesenchyme 5
Mesenchyme 6
Endothelial-like Cells
ACE2 WT (B10)
ACE2 KO (G7)
B
Endothelial-like Cells
Proliferating 1
Proliferating 2
Diff. Progenitors
LOH
Proximal Tubule
Podocytes 1
Podocytes 2
0.5
0
-0.5
M2_NPCs primed to diff
M4_Differentiation I
M1_NPCs
M12_Kidney Proliferation I
M13_Kidney Proliferation II
M6_Podocyte
M3_Differenitating NPCs
M5_Differentiation II
M10_LOH
M11_Connecting Tubule
M9_Proximal Tubule
M7_Medial/Proximal
M8_Medial/Distal
C
ACE2 WT (C9)
ACE2 WT (B10)
ACE2 KO (E3)
ACE2 KO (C6)
ACE2 KO (G7)
ACE2 KO (A10)
ACE2
CD147
LTL
MERGE + DAPI
D
Hallmark
ACE2 WT vs KO
PEROXISOME
ADIPOGENESIS
ESTROGEN RESPONSE EARLY
TNFA SIGNALING VIA NFKB
IL6 JAK STAT3 SIGNALING
REACTIVE OXYGEN SPECIES PATHWAY
XENOBIOTIC METABOLISM
FATTY ACID METABOLISM
BILE ACID METABOLISM
OXIDATIVE PHOSPHORYLATION
APOPTOSIS
KRAS SIGNALING UP
UV RESPONSE DN
WNT BETA CATENIN SIGNALING
MYC TARGETS V2
IL2 STAT5 SIGNALING
MTORC1 SIGNALING
GLYCOLYSIS
UNFOLDED PROTEIN RESPONSE
ANGIOGENESIS
APICAL JUNCTION
HYPOXIA
MITOTIC SPINDLE
EPITHELIAL MESENCHYMAL TRANSITION
MYC TARGETS V1
E2F TARGETS
G2M CHECKPOINT
Term
All
Podocytes 1
Podocytes 2
Diff. Progenitors
Proximal Tubule
LOH
Endothelial-like Cells
CellType
Direction
Upregulated in ACE2 WT vs KO
Downregulated in ACE2 WT vs KO
NES
1.4
1.6
1.8
2.0
2.2
-log10(adjusted p-value)
5
10
15
20
E
ACE2 WT (B10)
ACE2 KO (G7)
Mock
SARS-CoV-2
Mock
SARS-CoV-2
150
100
75
50
37
ACE2
β-ACTIN

Figure S6. SARS-CoV-2 Infection in *ACE2* KO Human Kidney Organoids, Related to Figure 4.

A) UMAP of *ACE2* WT kidney organoids (generated from clone B10) and *ACE2* KO kidney organoids (generated from *ACE2* KO clone G7) colored by annotated cell types.

B) Correlation analysis between scores for gene modules from second semester fetal-kidney cell types and scores for gene signatures from the annotated renal-like cell types in *ACE2* WT and *ACE2* KO kidney organoids. Scores were obtained with the *AddModuleScore* function from Seurat. Color scale denotes Pearson correlation coefficients.

C) Immunofluorescence staining for ACE2 (green), CD147 (red), LTL (grey) and DAPI (blue) in *ACE2* WT and *ACE2* KO kidney organoids. Scale bars, 250 μm, 50 μm (magnified views).

D) Hallmark gene set enrichment analysis (GSEA) was performed in kidney organoids generated from *ACE2* WT and *ACE2* KO lines. The ten gene sets per direction and sample with lowest adjusted p-value are shown. Circles are coded by color (direction), size (NES) and transparency (p-value).

E) Protein levels of ACE2 in Mock treated and SARS-CoV-2 infected (10^6 virus particles/organoid as determined in Vero cells) *ACE2* WT and *ACE2* KO kidney organoids are shown by Western Blot analysis. β-ACTIN was used as loading control. Data from a pool of 12 organoids/group is shown.

Figure S7

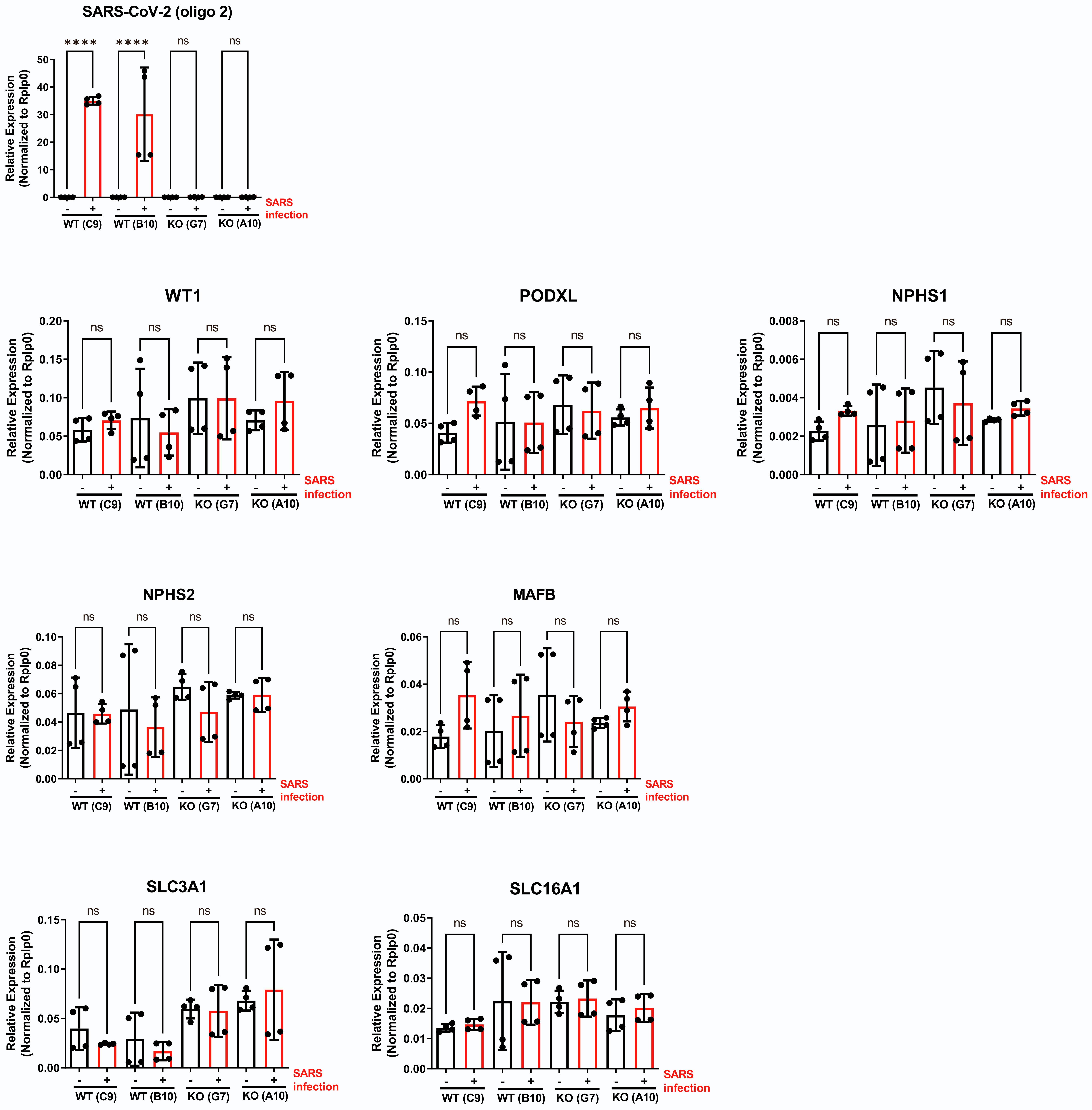
SARS-CoV-2 (oligo 2)
Relative Expression (Normalized to Rplp0)
****
****
ns
ns
WT (C9)
WT (B10)
KO (G7)
KO (A10)
SARS infection
WT1
PODXL
NPHS1
NPHS2
MAFB
SLC3A1
SLC16A1

Figure S7. SARS-CoV-2 Infection in *ACE2* KO Human Kidney Organoids, Related to Figure 4.

Quantitative real time analysis was performed at 3 dpi in Mock or SARS-CoV-2 infected (10^6 virus particles/organoid as determined in Vero cells) *ACE2* WT and *ACE2* KO kidney organoids to quantify the mRNA expression levels of SARS-CoV-2, podocyte markers (*WT1*, *PODXL*, *NPHS1*, *NPHS2*, *MAFB*), and proximal tubular markers (*SLC3A1* and *SLC16A1*). Data from a pool of 12 organoids/group is shown. The data are represented as mean ± s.d. $n = 2$ independent experimental replicates from a pool of 12 organoids/group with at least two technical replicates each. ****$P < 0.0001$, ns, no statistical significance, One-way ANOVA, Tukey's multiple comparisons test.

Figure S8

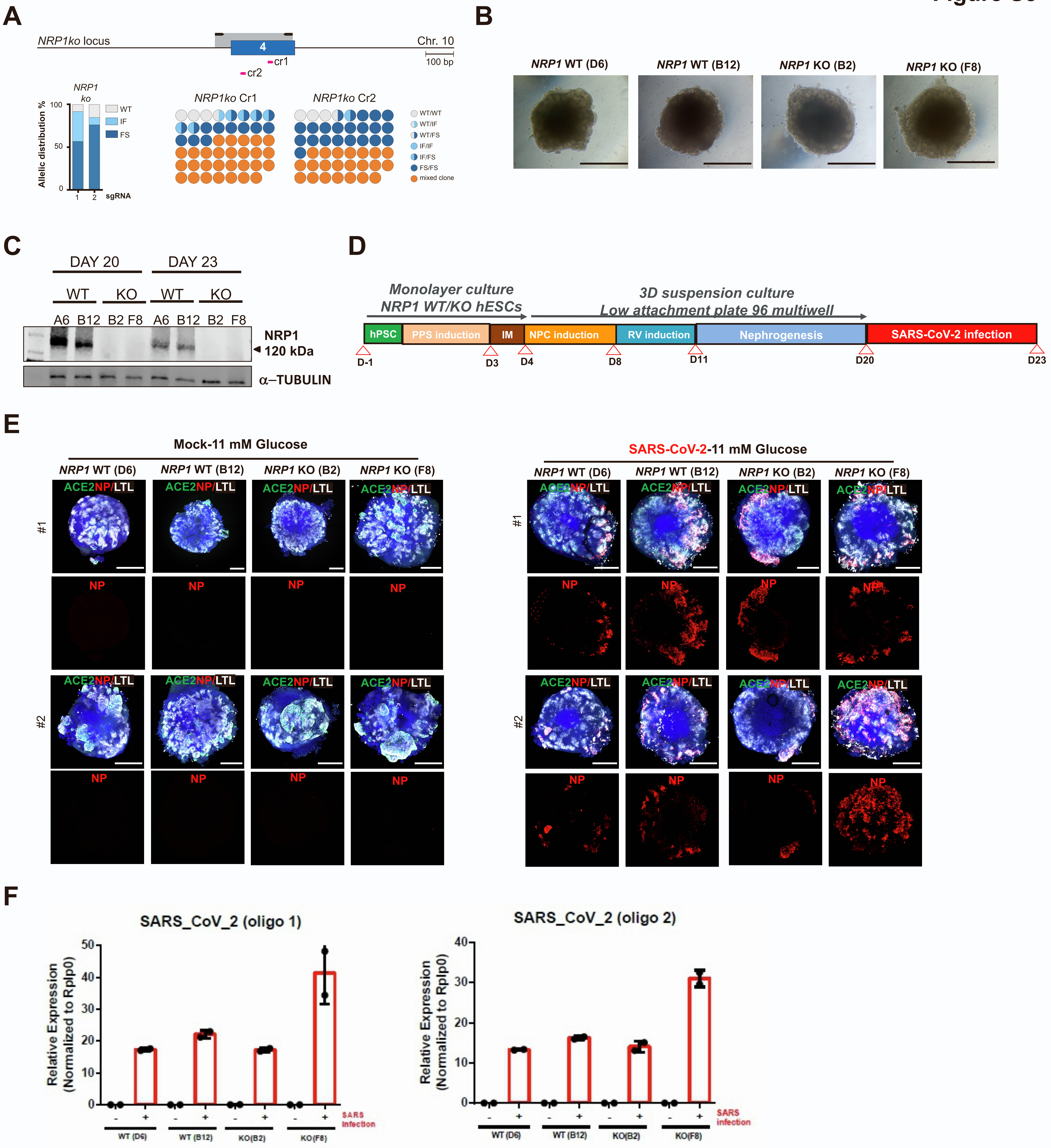

A
NRP1ko locus
4
Chr. 10
100 bp
cr1
cr2
NRP1 ko
Allelic distribution %
WT
IF
FS
sgRNA
NRP1ko Cr1
NRP1ko Cr2
WT/WT
WT/IF
WT/FS
IF/IF
IF/FS
FS/FS
mixed clone
B
NRP1 WT (D6)
NRP1 WT (B12)
NRP1 KO (B2)
NRP1 KO (F8)
C
DAY 20
DAY 23
WT
KO
A6 B12 B2 F8 A6 B12 B2 F8
NRP1
120 kDa
α–TUBULIN
D
Monolayer culture
NRP1 WT/KO hESCs
3D suspension culture
Low attachment plate 96 multiwell
hPSC
PPS induction
IM
NPC induction
RV induction
Nephrogenesis
SARS-CoV-2 infection
D-1
D3
D4
D8
D11
D20
D23
E
Mock-11 mM Glucose
SARS-CoV-2-11 mM Glucose
ACE2NP/LTL
NP
#1
#2
F
SARS_CoV_2 (oligo 1)
SARS_CoV_2 (oligo 2)
Relative Expression (Normalized to Rplp0)
SARS infection
WT (D6)
WT (B12)
KO(B2)
KO(F8)

Figure S8. Generation and Infection of *NRP1* KO Human Kidney Organoids, Related to Figure 4 and Figure 5.

A) Schematic of Cas9/gRNA-targeting sites (pink arrows) in the *NRP1 locus* showing exon structure (blue boxes) and PCR amplicons (light gray boxes). Histogram shows allelic sequence distribution after the transfection of the different gRNAs in undifferentiated ES[4] cells expressing an inducible Cas9 (iCas9). wt, wild-type; mut, mutation; FS, frameshift.

B) Representative bright field images of *NRP1* WT and *NRP1* KO kidney organoids. Scale bars, 250 μm.

C) Protein levels of NRP1 in *NRP1* WT and *NRP1* KO kidney organoids are shown by Western blot analysis. αTUBULIN was used as loading control. Data from a pool of 12 organoids/group is shown.

D) Experimental scheme for the generation and viral infection of *NRP1* WT and *NRP1* KO kidney organoids.

E) Immunofluorescence was performed at 3 dpi in Mock treated or SARS-CoV-2 infected (10^6 virus particles/organoid as determined in Vero cells) *NRP1* WT and *NRP1* KO kidney organoids. Representative confocal images show the detection of ACE2 (green), viral nuclear protein (NP, red), LTL (grey) and DAPI (blue). Scale bars, 250 μm.

F) Quantitative real time analysis was performed at 3 dpi in Mock treated or SARS-CoV-2 infected (10^6 virus particles/organoid as determined in Vero cells) *NRP1* WT and *NRP1* KO specimens for the detection of SARS-CoV-2 mRNA expression levels. The data are represented as mean ± s.d. n = 1 independent experiment from a pool of 12 organoids/group with at least two technical replicates each.

Figure S9

A

	Non Diabetic	Diabetic
**Age (yr)***	58.0 ± 23.07	71.3 ± 5.86
**Sex, F/M**	1/2	0/3
**Diabetic onset**	N/A	Type 2

*** Mean ± SD**

B

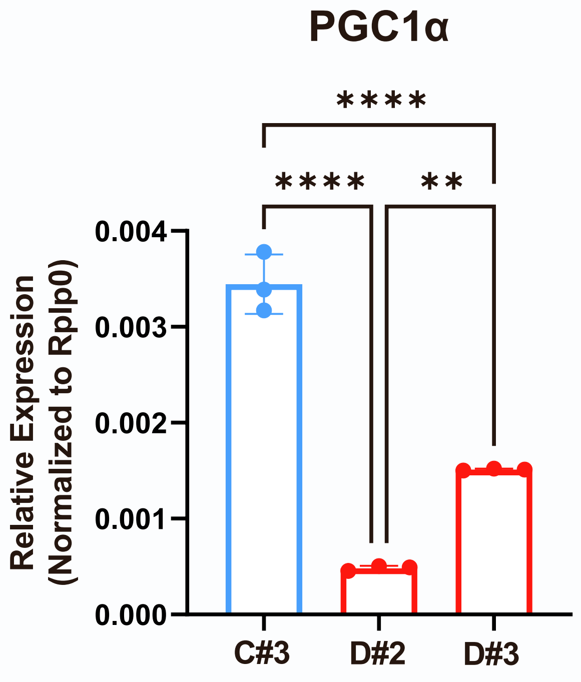

C

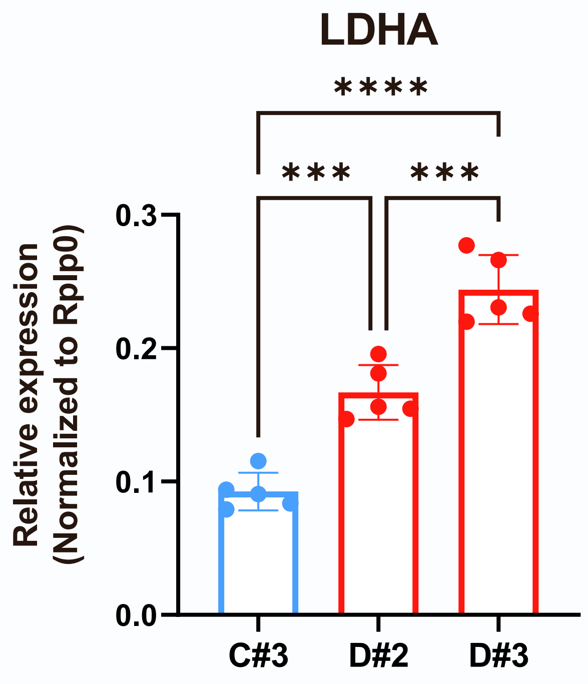

D

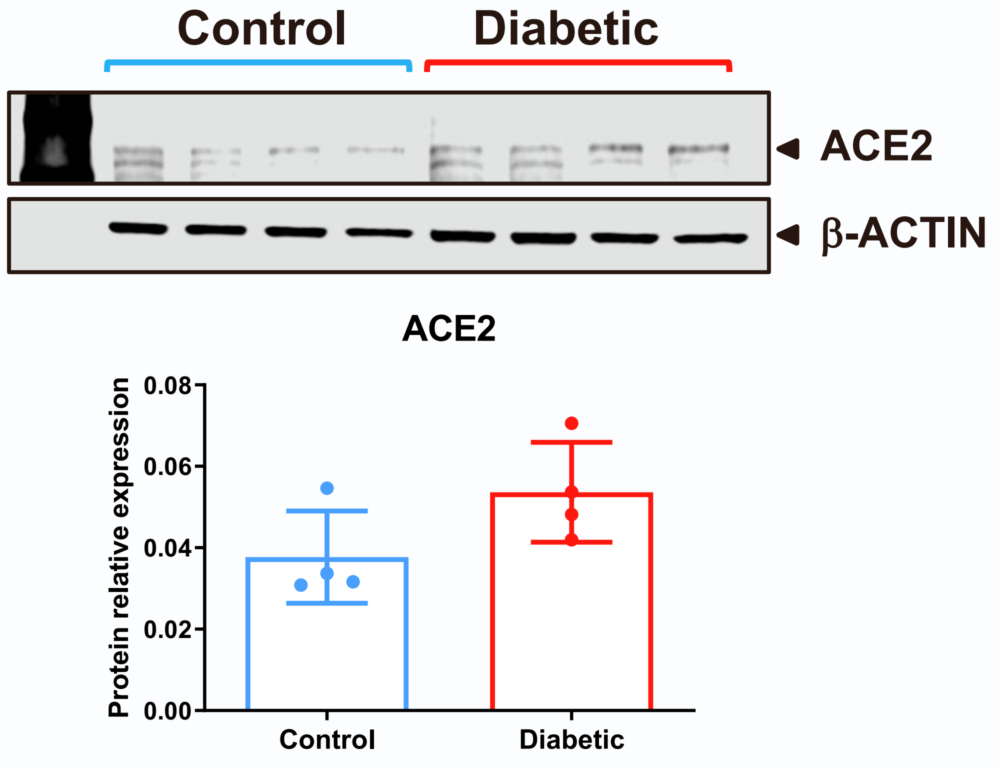

E

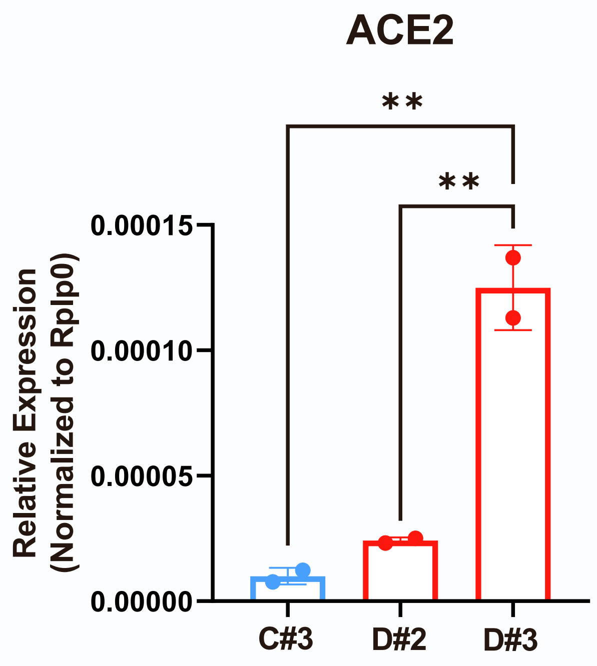

F

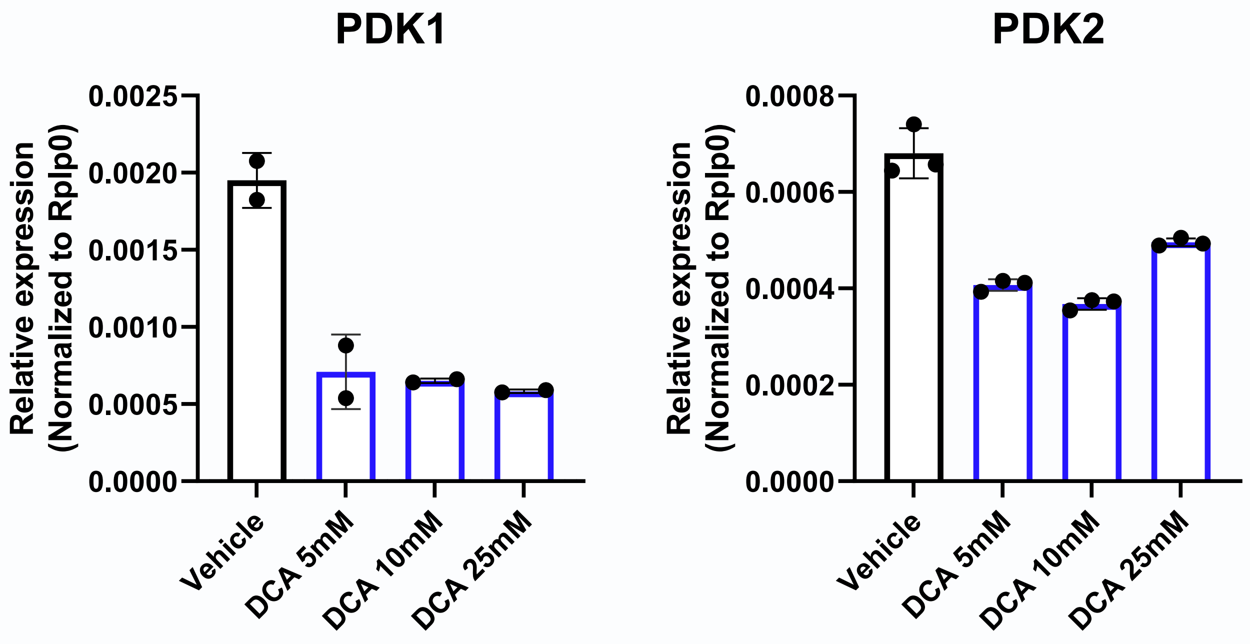

Figure S9. SARS-CoV-2 Infection in Patient Kidney Cells, Related to Figure 7.

A) Characteristics of non-diabetic (control) and diabetic patients, from which primary human proximal tubular cells (HPTCs) were isolated and used in this study.

B) mRNA expression levels of *PGC1α* in Control and Diabetic HPTCs determined by qPCR. The data are presented as mean ± s.d. of at least $n = 1$ independent biological replicate per condition with three technical replicates each. $^{**}P < 0.005$, $^{****}P < 0.0001$, One-way ANOVA, Tukey's multiple comparisons test.

C) mRNA expression levels of *LDHA* by qPCR in Control and Diabetic HPTCs. The data are presented as mean ± s.d. of at least $n = 1$ independent biological replicate per condition with five technical replicates each. $^{***}P < 0.001$, $^{****}P < 0.0001$, One-way ANOVA, Tukey's multiple comparisons test.

D) Protein levels of ACE2 in HPTCs from Control and Diabetic donors as determined by Western blotting. β-ACTIN was used as loading control. The data are presented as mean ± s.d. of $n = 2$ independent biological replicates per condition with two technical replicates each.

E) mRNA expression levels of *ACE2* by qPCR in Control and Diabetic HPTCs. The data are presented as mean ± s.d. of at least $n = 1$ independent experiment with two technical replicates each. $^{**}P < 0.005$, One-way ANOVA, Tukey's multiple comparisons test.

F) mRNA expression levels of *PDK1* and *PDK2* in HPTCs after treatment with different doses of dichloroacetate (DCA) or vehicle, determined by qPCR. The data are presented as mean ± s.d. of $n = 1$ independent experiment with at least two technical replicates each.
