# Supplementary material for: A diabetic milieu increases ACE2 expression and cellular susceptibility to SARS-CoV-2 infections in human kidney organoids and patient cells
Source: Cell Metab. 2022 Jun 7;34(6):857–873.e9. doi: 10.1016/j.cmet.2022.04.009 (PMC9097013; doi:10.1016/j.cmet.2022.04.009)
Supplement: Data S1. Unprocessed source data underlying all blots and graphs and supporting data, related to Figures 1–7 and S1–S9 [file mmc2.zip › DataS1 Source Data/Data S1_Microscopy Items.pdf]

Extended data 1 -Related to Figure 1

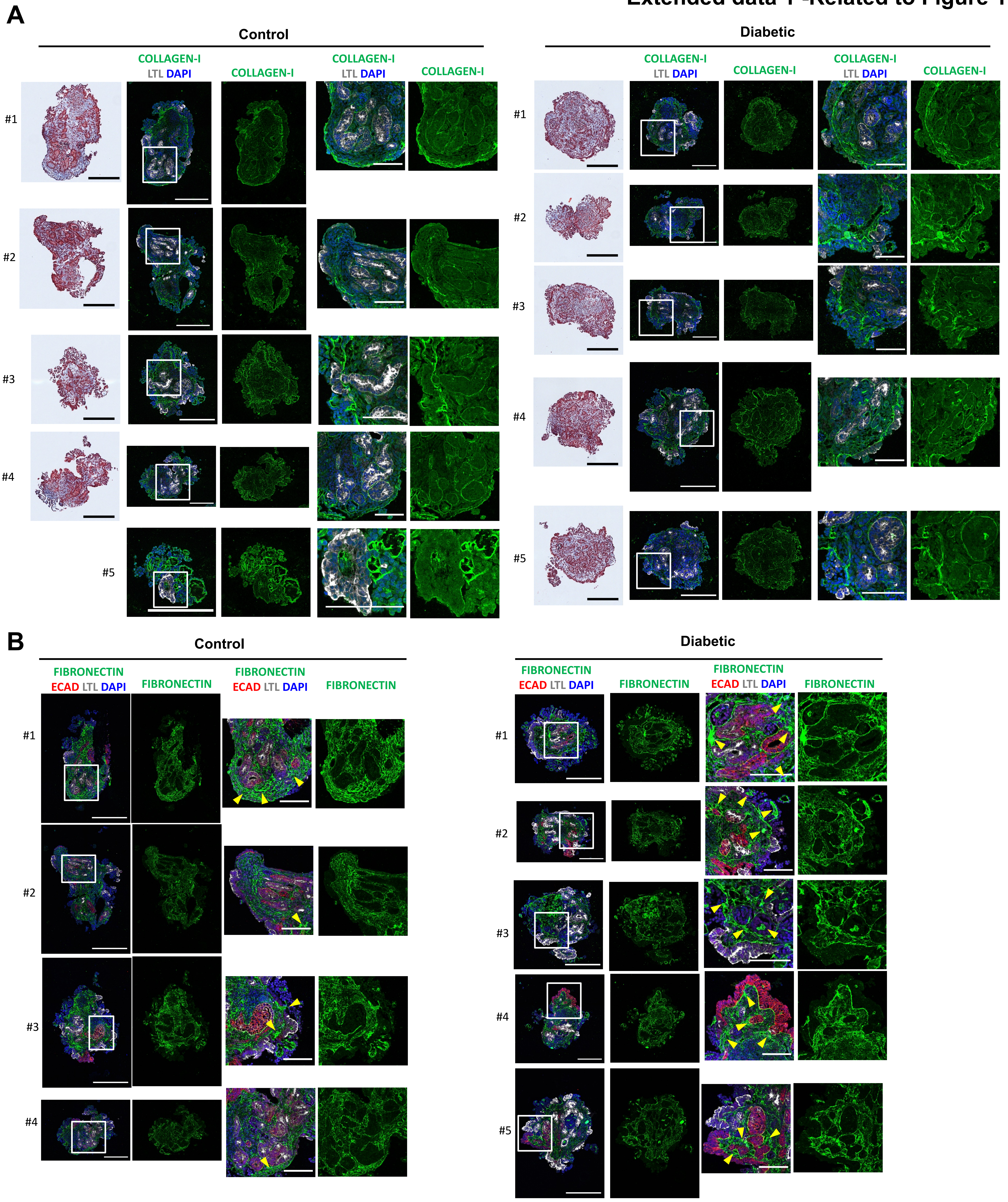

Extended Data 1, Related to Figure 1.

A) Representative Trichrome Masson staining in kidney organoids exposed to Control or Diabetic conditions for 7 days. Scale bars, 250 µm. Consecutive sections were stained for Collagen-I (green), LTL (grey) and DAPI (blue). Scale bars, 250 µm, 100 µm (magnified views). *n* = 5 organoids/group.

B) Representative immunofluorescence staining of Fibronectin (green), E-cadherin (ECAD; red), LTL (grey) and DAPI (blue) exposed to Control or Diabetic conditions for 7 days. Scale bars, 250 µm, 100 µm (magnified views). *n* = 4 Control organoids; *n* = 5 Diabetic organoids.

Extended Data 2 -Related to Figure 1

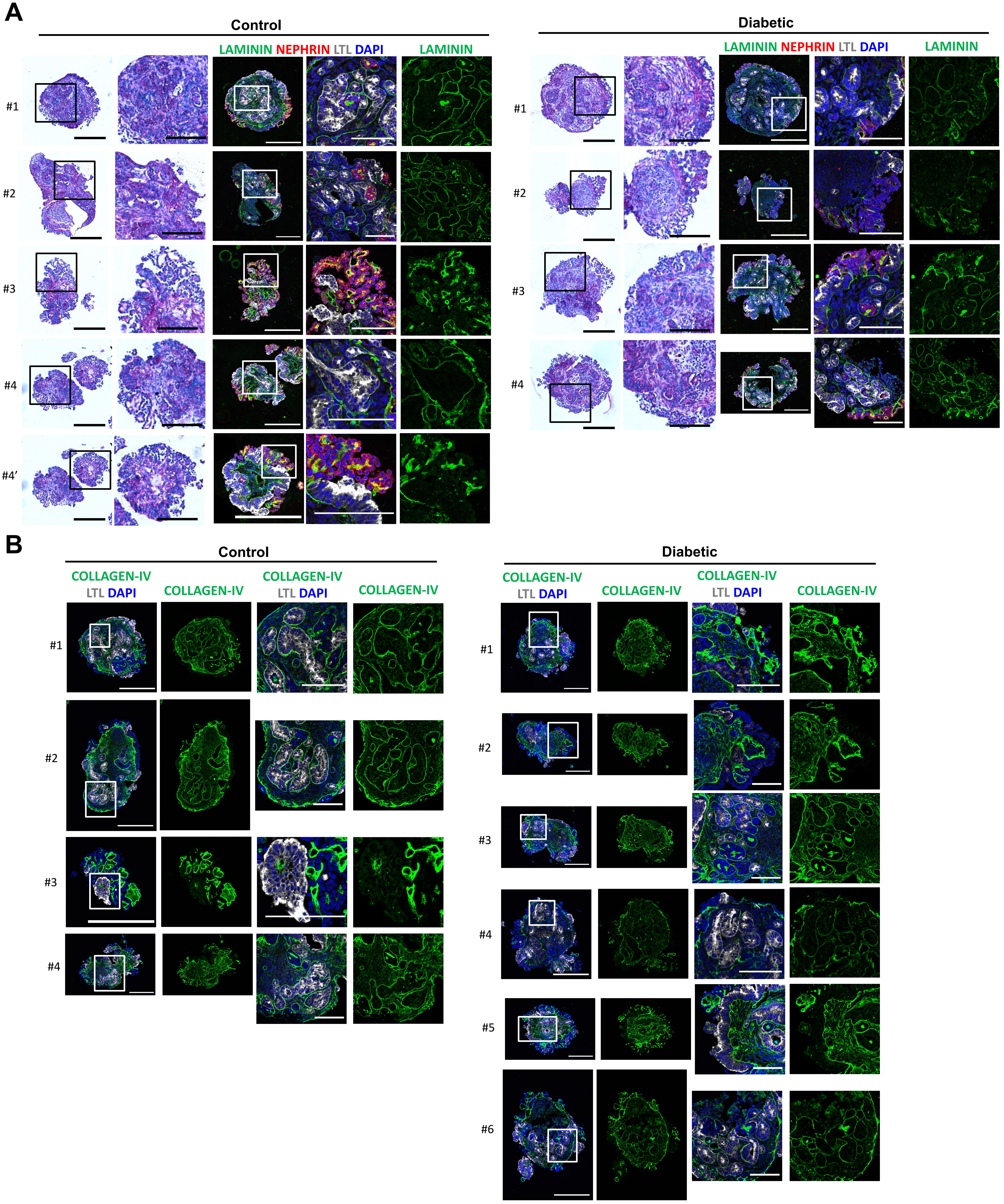

Extended Data 2, Related to Figure 1.

A) Representative Periodic Acid-Schiff (PAS) staining in kidney organoids exposed to Control or Diabetic conditions for 7 days. Scale bars, 250 μm, 100 μm (magnified views). Consecutive sections were stained for Laminin (green), Nephrin (red), LTL (grey) and DAPI (blue). Scale bars, 250 μm, 100 μm (magnified views). *n* = 4 organoids/group.

B) Representative immunofluorescence staining of Collagen-IV (green), LTL (grey) and DAPI (blue) in kidney organoids exposed to Control or Diabetic conditions for 7 days. Scale bars, 250 μm, 100 μm (magnified views). *n* = 4 Control organoids; *n* = 6 Diabetic organoids.

Extended Data 3 -Related to Figure 2

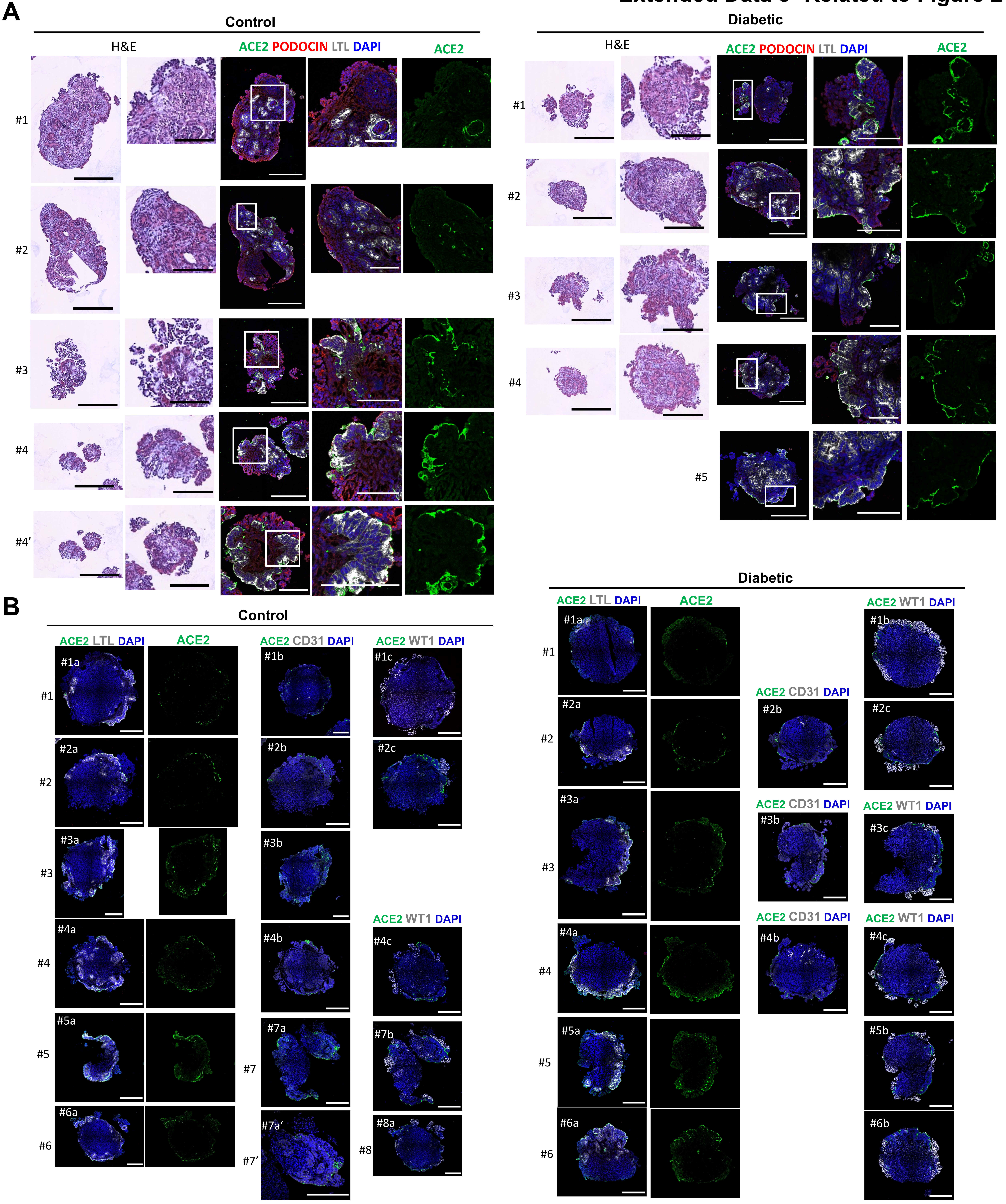

Extended Data 3, Related to Figure 2.

A) Representative Hematoxylin and Eosin (HE) staining in kidney organoids exposed to Control or Diabetic conditions for 7 days. Scale bars, 250 μm, 100 μm (magnified views). Consecutive sections were stained for ACE2 (green), Podocin (red), LTL (grey) and DAPI (blue). Scale bars, 250 μm, 100 μm (magnified views). *n* = 4 Control organoids; *n* = 5 Diabetic organoids.

B) Representative immunofluorescence stainings of kidney organoids exposed to Control or Diabetic conditions for 7 days. Consecutive sections were stained for ACE2 (green) /LTL (grey) /DAPI (blue), ACE2 (green) /CD31 (grey) /DAPI (blue) and ACE2 (green) /WT1 (grey) /DAPI (blue). Scale bars, 200 μm. *n* = 8 Control organoids; *n* = 6 Diabetic organoids.

Extended Data 4, Related to Figure 2

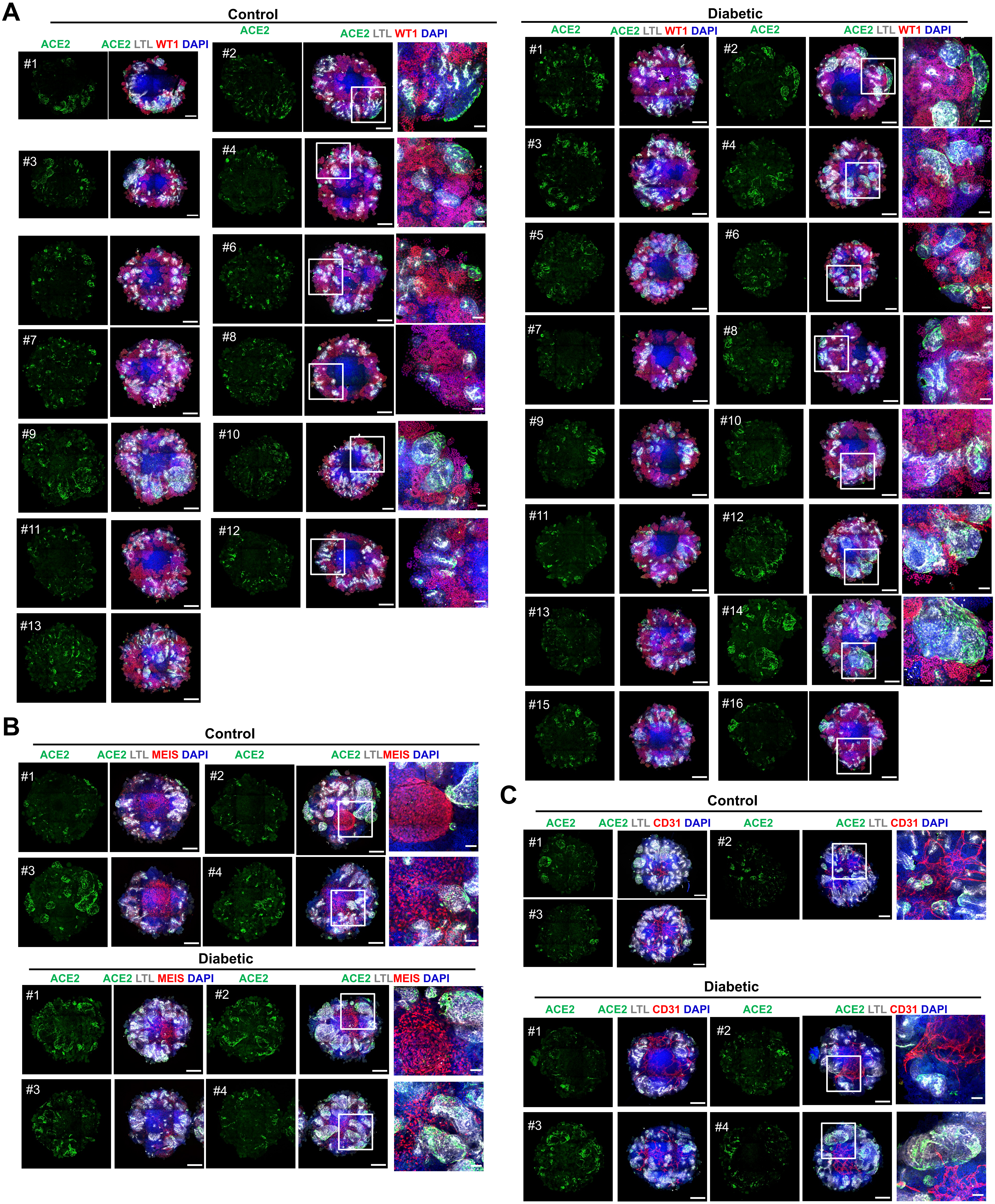

Extended Data 4, Related to Figure 2.

A) Whole mount immunofluorescence staining of ACE2 (green), WT1 (red), LTL (grey) and DAPI (blue) in kidney organoids exposed to Control or Diabetic conditions for 7 days. Scale bars, 200 μm, 50 μm (magnified views). *n* = 12 Control organoids; *n* = 16 Diabetic organoids.

B) Whole mount immunofluorescence staining of CD31 (red), LTL (grey) and DAPI (blue) in kidney organoids exposed to Control or Diabetic conditions for 7 days. Scale bars, 200 μm, 50 μm (magnified views). *n* = 4 Control organoids; *n* = 4 Diabetic organoids.

C) Whole mount immunofluorescence staining of MEIS (red), LTL (grey) and DAPI (blue) in kidney organoids exposed to Control or Diabetic conditions for 7 days. Scale bars, 200 μm, 50 μm (magnified views). *n* = 3 Control organoids; *n* = 4 Diabetic organoids.

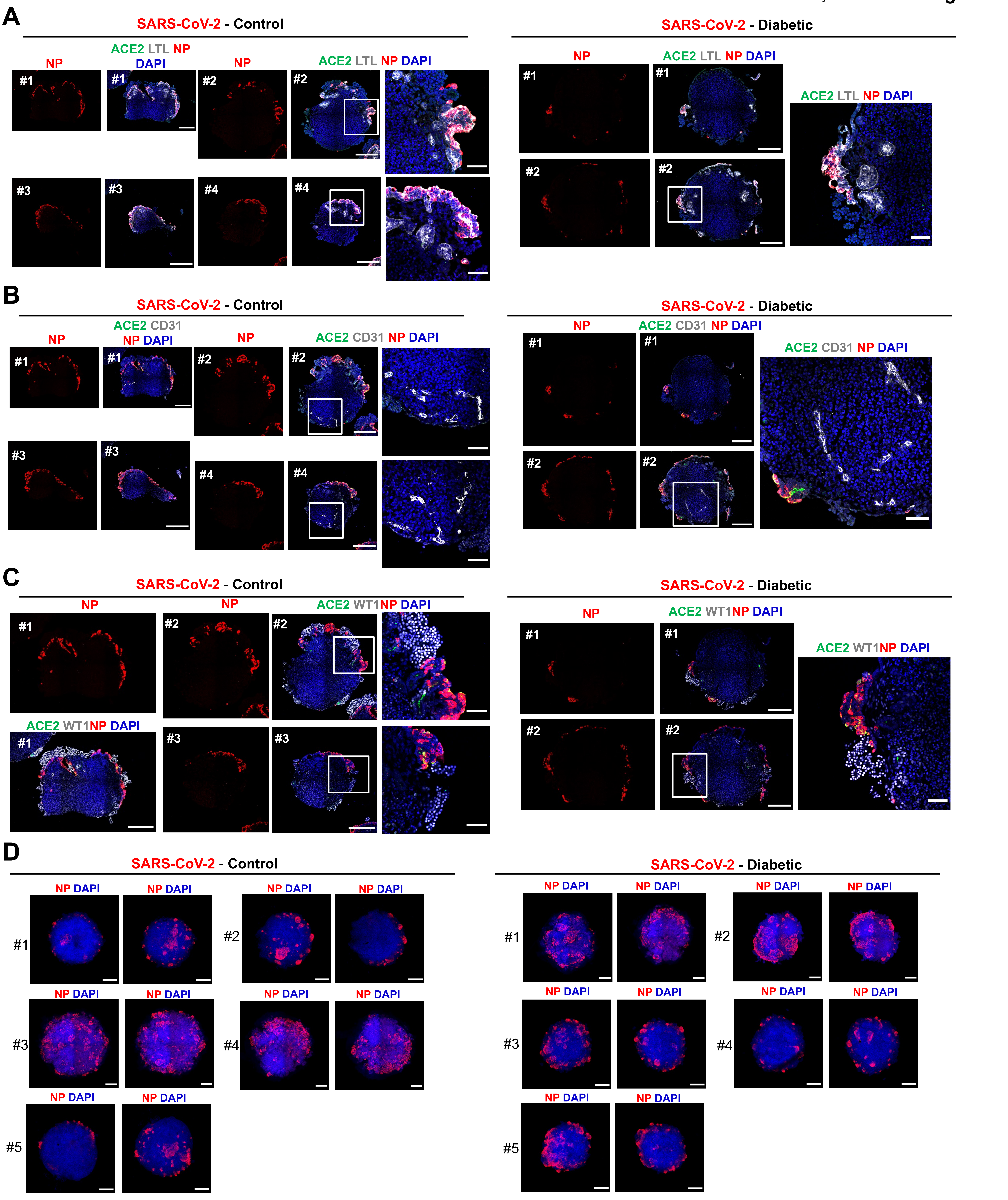
A
SARS-CoV-2 - Control
NP
ACE2 LTL NP DAPI
#1
#2
#3
#4
SARS-CoV-2 - Diabetic
B
SARS-CoV-2 - Control
ACE2 CD31 NP DAPI
SARS-CoV-2 - Diabetic
C
SARS-CoV-2 - Control
ACE2 WT1NP DAPI
SARS-CoV-2 - Diabetic
D
SARS-CoV-2 - Control
NP DAPI
#5
SARS-CoV-2 - Diabetic

Extended Data 5, Related to Figure 2.

A) Immunofluorescence staining of consecutive sections of SARS-CoV-2 infected (10^6 virus particles/organoid as determined in Vero cells) Control or Diabetic kidney organoids at 1 day post infection (1 dpi) for the detection of ACE2 (green), viral nuclear protein (NP; red), LTL (magenta) and DAPI (blue). Scale bars, 200 μm, 50 μm (magnified views). *n* = 4 SARS-CoV-2 Control organoids; *n* = 2 SARS-CoV-2 Diabetic organoids.

B) Immunofluorescence staining of SARS-CoV-2 infected (10^6 virus particles/organoid as determined in Vero cells) Control or Diabetic kidney organoids at 1 day post infection (1 dpi) for the detection of ACE2 (green), viral nuclear protein (NP; red), WT1 (magenta) and DAPI (blue). Scale bars, 200 μm, 50 μm (magnified views). *n* = 4 SARS-CoV-2 Control organoids; *n* = 2 SARS-CoV-2 Diabetic organoids.

C) Immunofluorescence staining of SARS-CoV-2 infected (10^6 virus particles/organoid as determined in Vero cells) Control or Diabetic kidney organoids at 1 day post infection (1 dpi) for the detection of ACE2 (green), viral nuclear protein (NP; red), CD31 (magenta) and DAPI (blue). Scale bars, 200 μm, 50 μm (magnified views). *n* = 3 SARS-CoV-2 Control organoids. *N* = 2 SARS-CoV-2 Diabetic organoids.

D) Whole mount immunofluorescence staining of SARS-CoV-2 infected (10^6 virus particles/organoid as determined in Vero cells) Control or Diabetic kidney organoids at 1 day post infection (1 dpi) for the detection of viral nuclear protein (NP; red) and DAPI (blue). Scale bars, 200 μm. *n* = 5 SARS-CoV-2 Control organoids; *n* = 5 SARS-CoV-2 Diabetic organoids.

Extended Data 6, Related to Figure 6

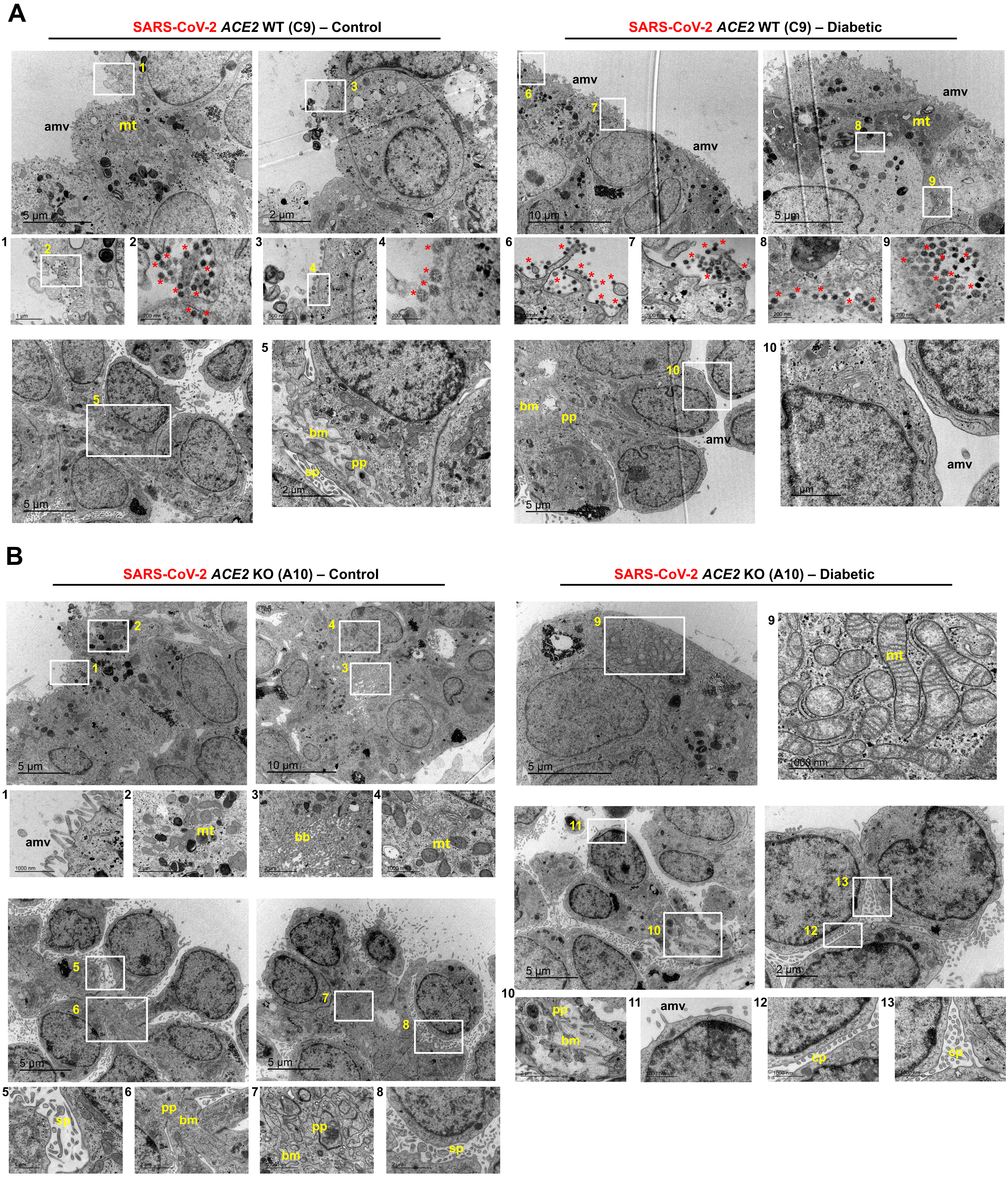

Extended Data 6, Related to Figure 6.

A) TEM analysis of *ACE2* WT (C9) kidney organoids exposed to Control or Diabetic conditions infected with SARS-CoV-2 (10^6 virus particles/organoid as determined in Vero cells) and recovered at 1 dpi. Representative images of infected *ACE2* WT (C9) Control specimen show multiple viral particles (asterisks) in contact with the apical microvilli (amv) of tubular-like cells (1-4). Details for podocyte-like cells exhibiting podocyte related-structures including primary processes (pp) (5) and the deposition of a basement membrane (bm) (5) are shown. Scale bars, 5 µm, 2 µm; 1 µm (1); 200 nm (2); 500 nm (3); 200 nm (4); 2 µm (5). Representative images of infected ACE2 WT (C9) Diabetic specimen show numerous viral particles (asterisks) in contact with the apical microvilli (amv) of tubular-like cells (6,7) and in-between tubular-like cells in close contact with cell membranes (8,9). Podocyte-like cells with apical microvilli (amv), primary cell processes (pp) and basal deposition of a basement membrane are also shown (10). Scale bars, 10 µm, 5 µm; 500 nm (6,7); 200 nm (8,9); 1 µm (10).

B) TEM analysis of *ACE2* KO (A10) kidney organoids exposed to Control or Diabetic conditions infected with SARS-CoV-2 (10^6 virus particles/organoid as determined in Vero cells) and recovered at 1 dpi. Representative images of infected *ACE2* KO (A10) Control specimen show tubular-like cells with apical microvilli (amv), high mitochondrial content (mt) and dense brush borders (bb) (1-4). Glomerular-like structures with podocyte-like cells that exhibit primary (pp) and secondary (sp) cell processes, and deposition of basement membrane (bm) are shown (5-8). Scale bars, 5 µm, 10 µm; 1 µm (1,4,5); 2 µm (2,3,6,8). Representative images of infected *ACE2* KO (A10) Diabetic specimen show tubular-like cells with high mitochondrial content (mt) (9). Podocyte-like cells display primary cell processes (pp), deposition of basement membrane (bm) and apical microvilli (amv) (10-13). Scale bars, 5 µm, 2 µm; 1 µm (9,11-13); 2 µm (10).
