# Supplementary figures and images for: A diabetic milieu increases ACE2 expression and cellular susceptibility to SARS-CoV-2 infections in human kidney organoids and patient cells
Source: Cell Metab. 2022 Jun 7;34(6):857–873.e9. doi: 10.1016/j.cmet.2022.04.009 (PMC9097013; doi:10.1016/j.cmet.2022.04.009)

Figure 2G

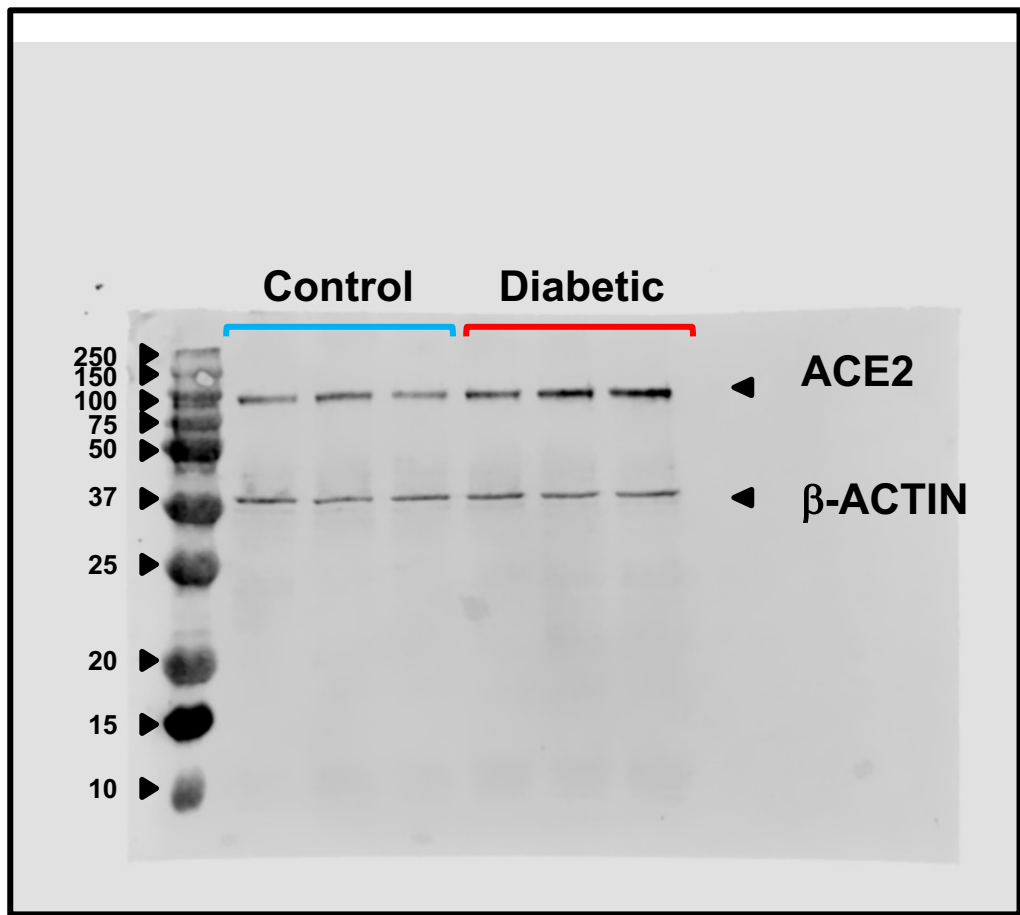

Figure 5E

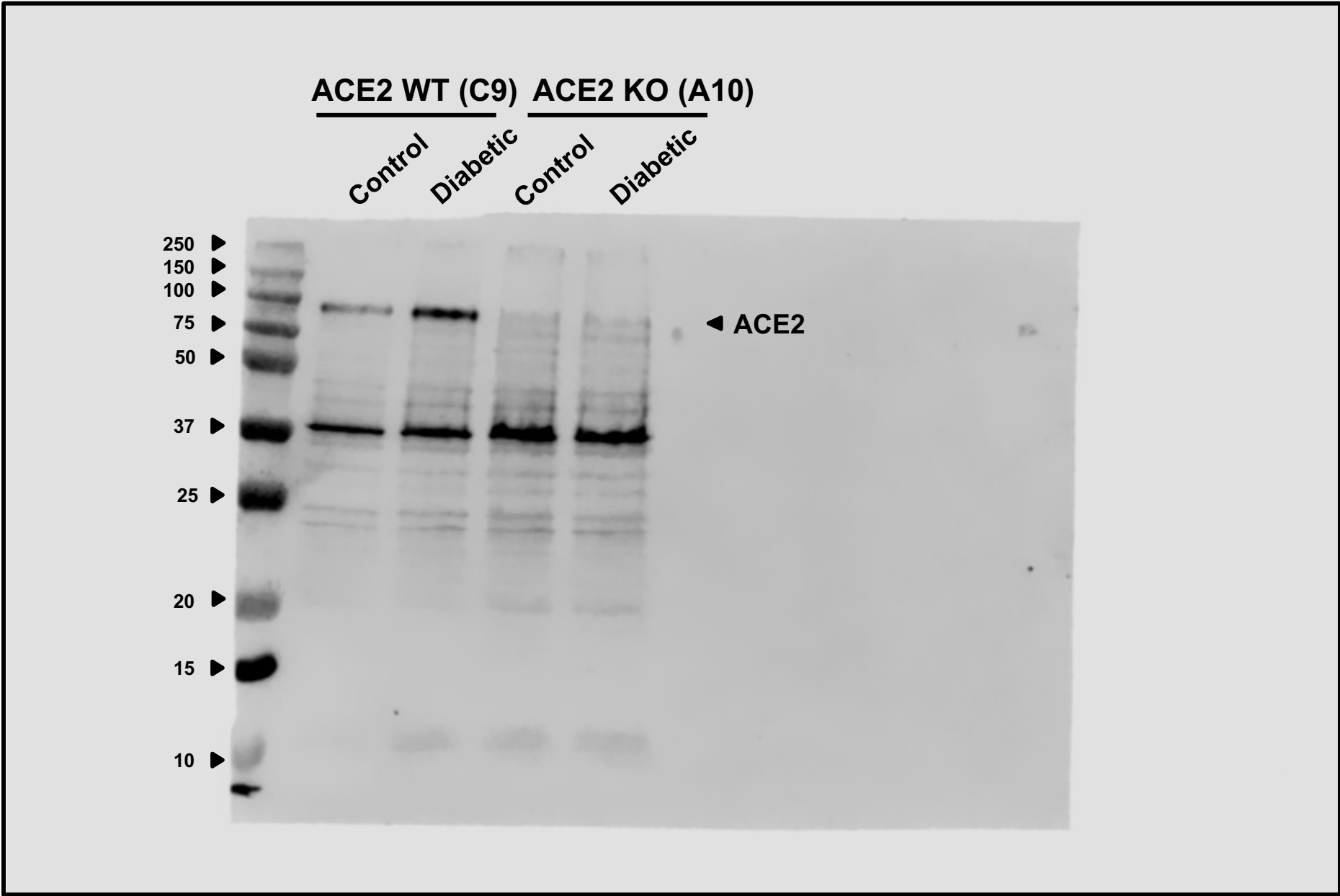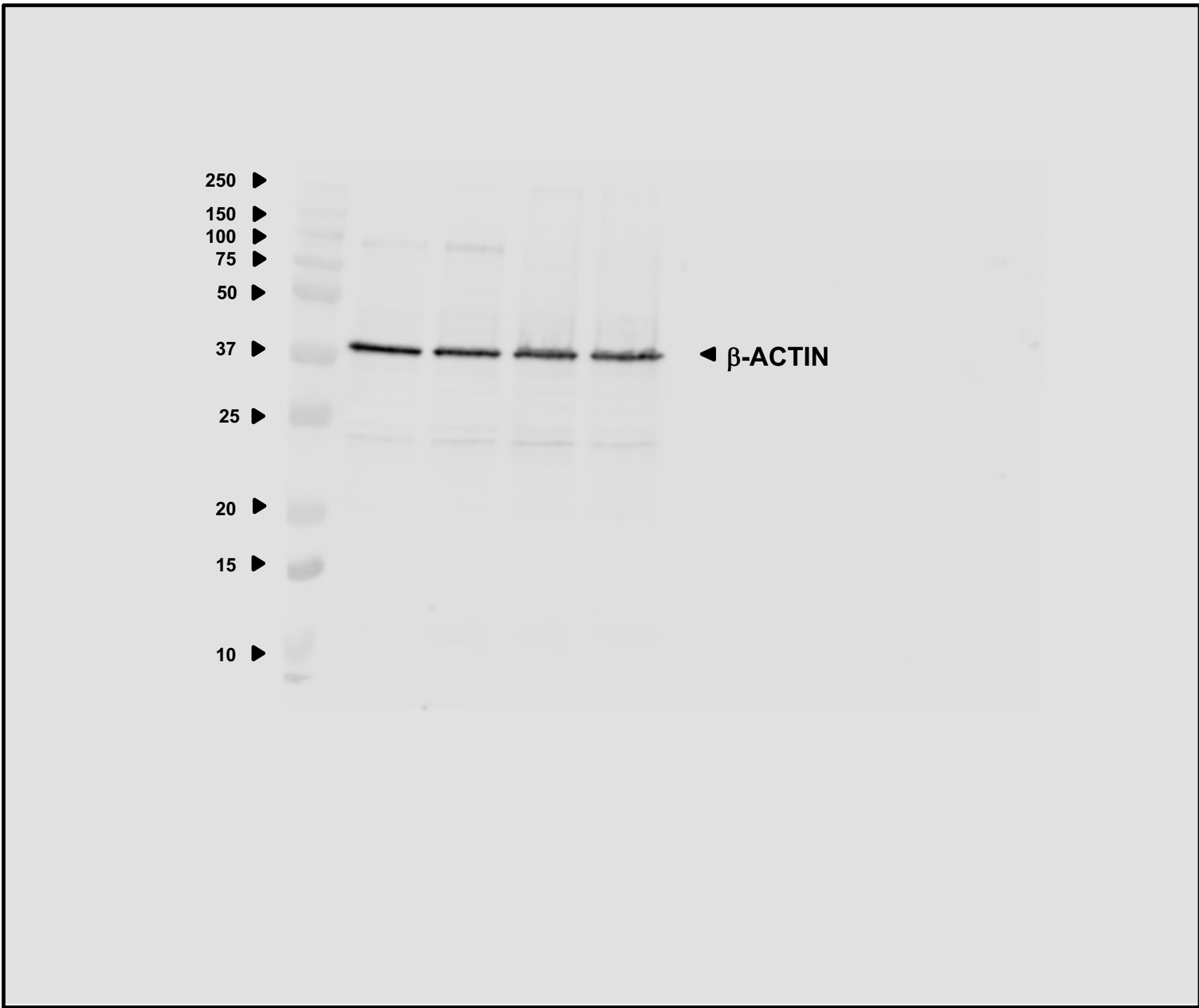

Figure S5D

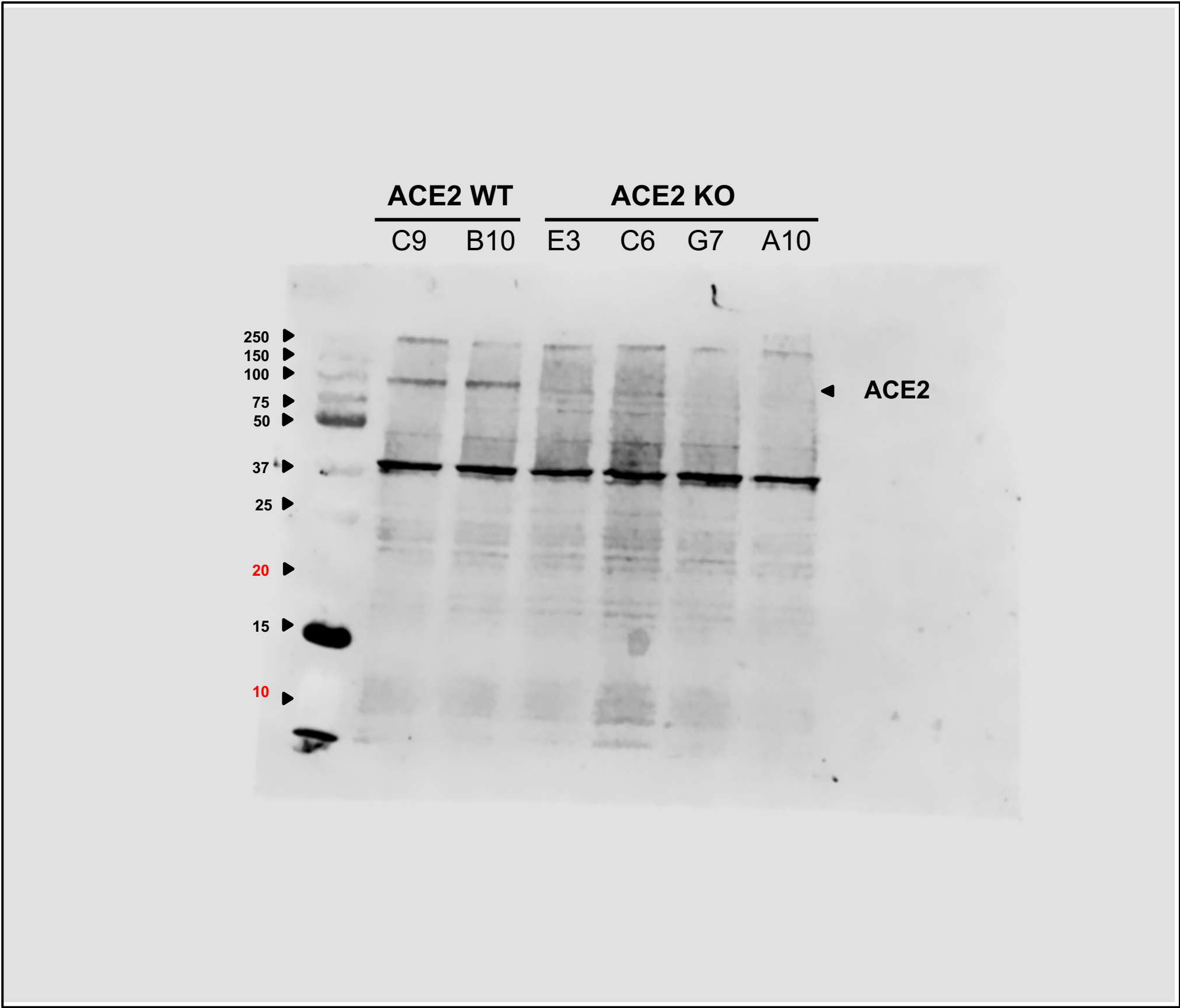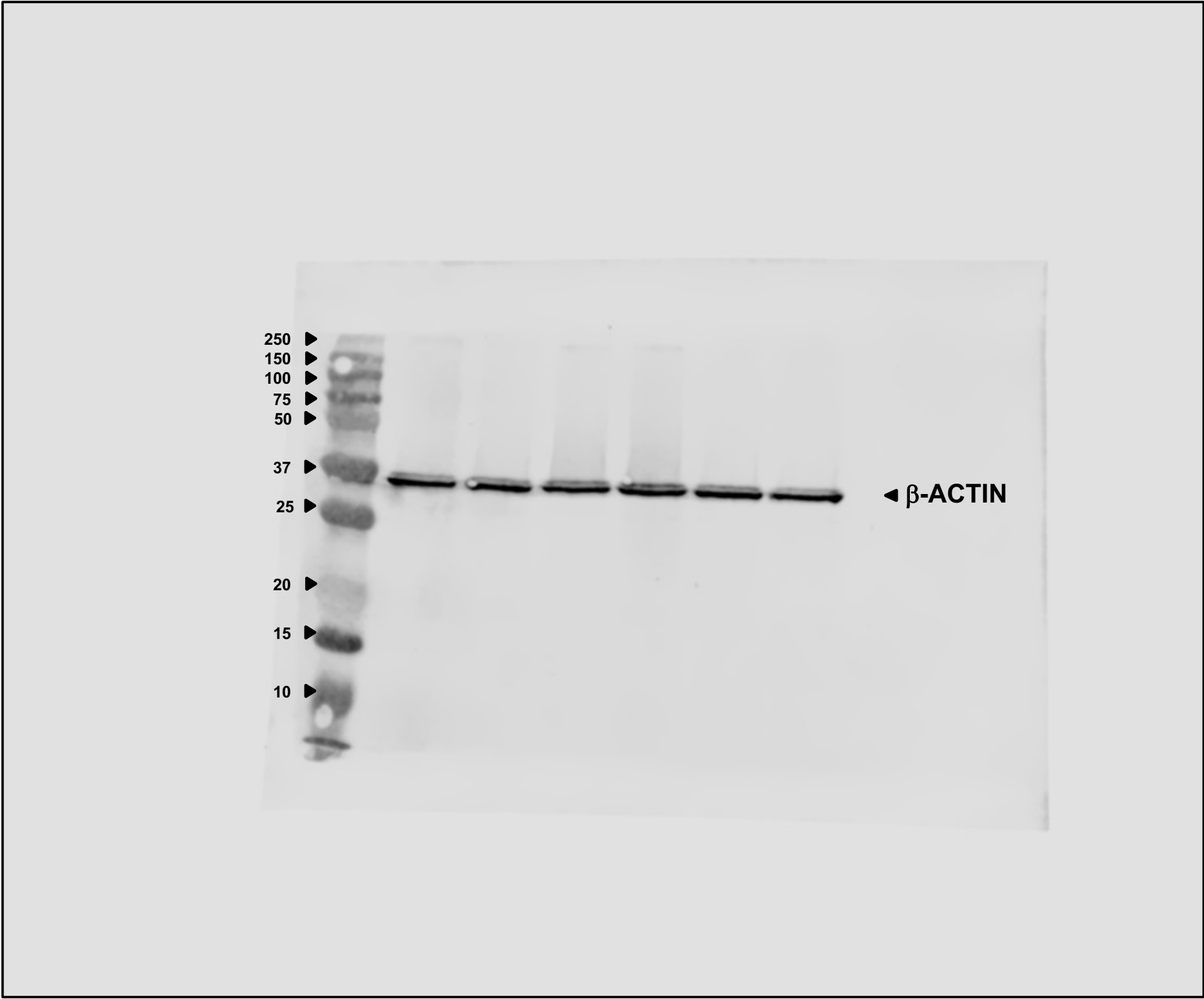

Figure S6E

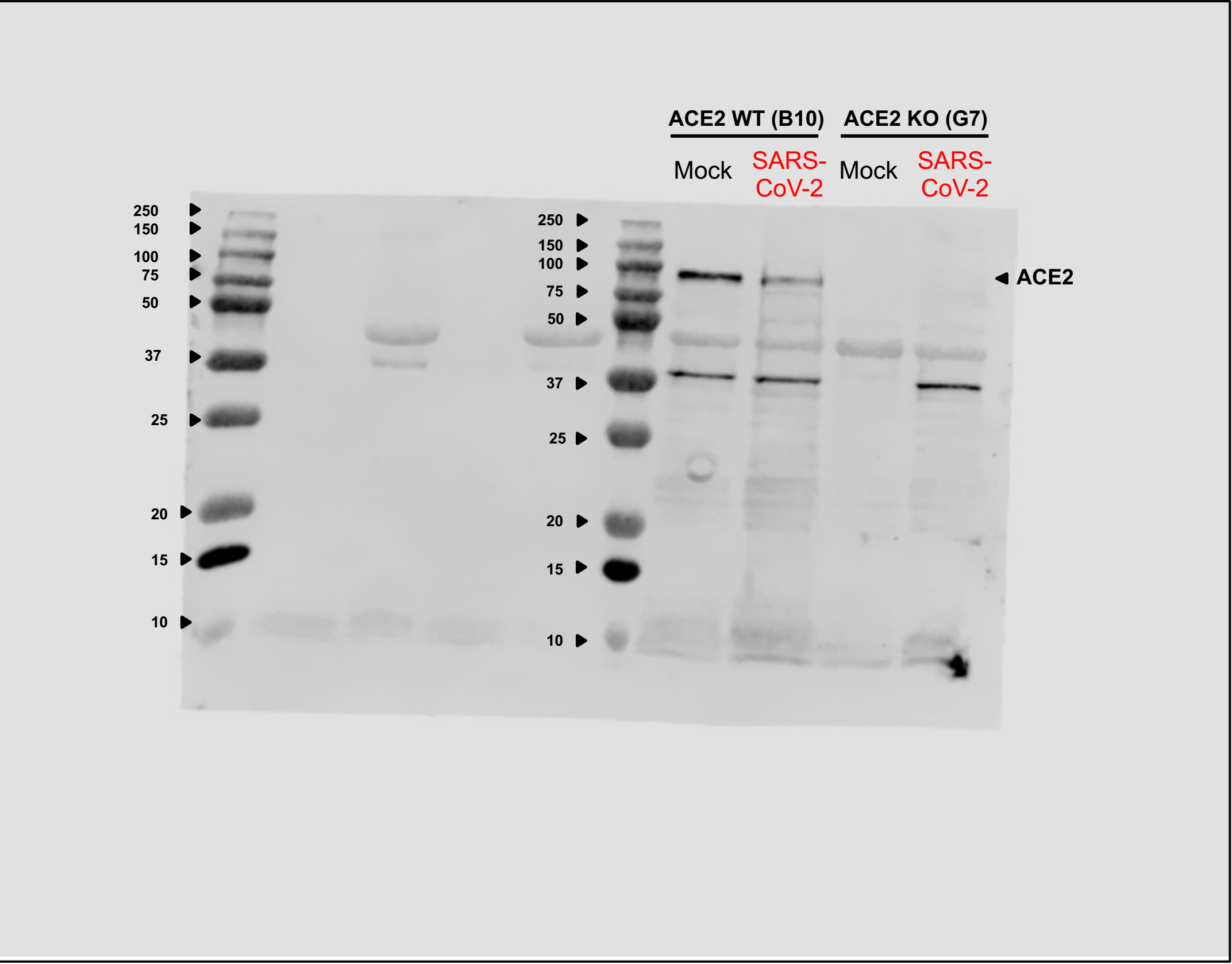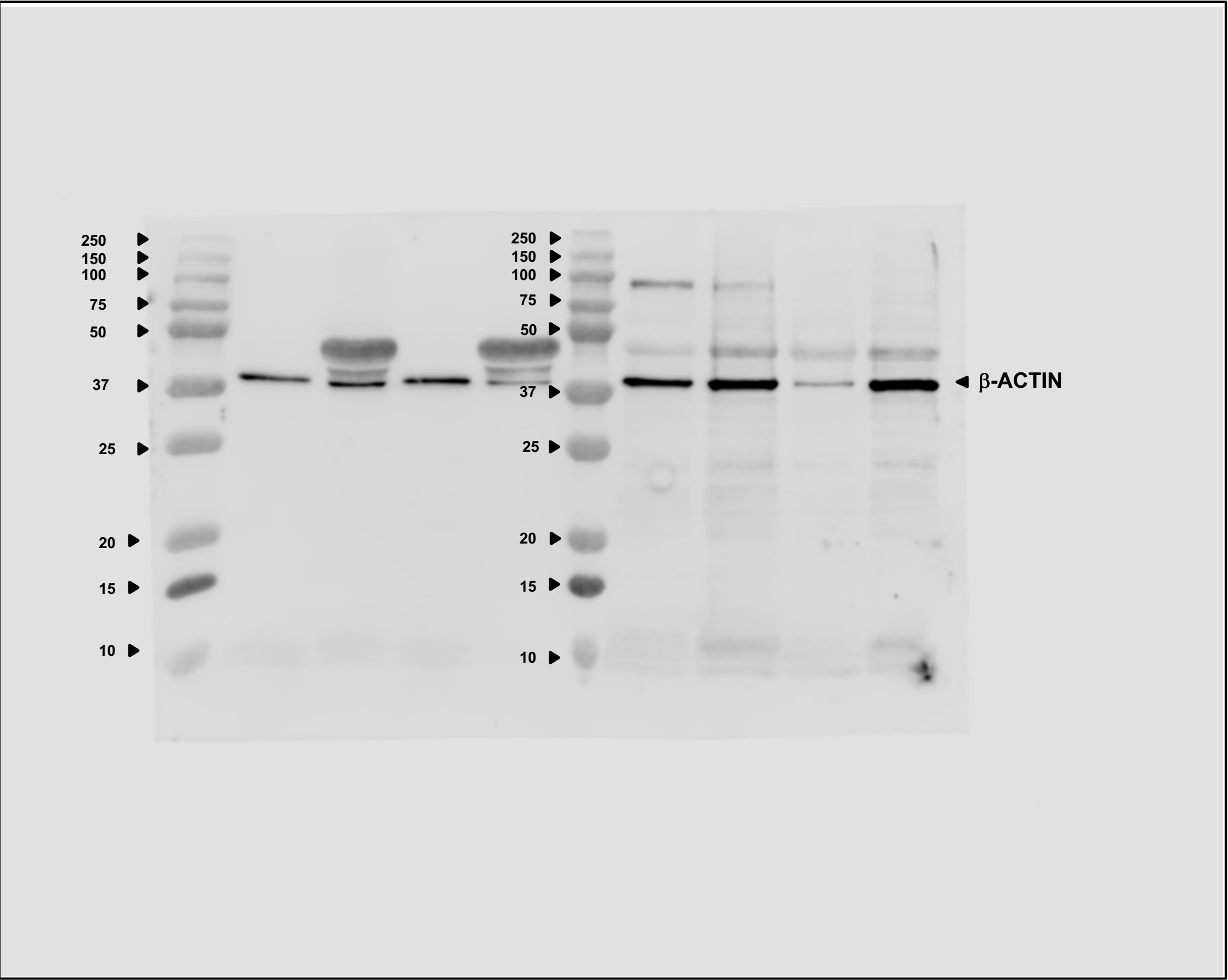

Figure S8C

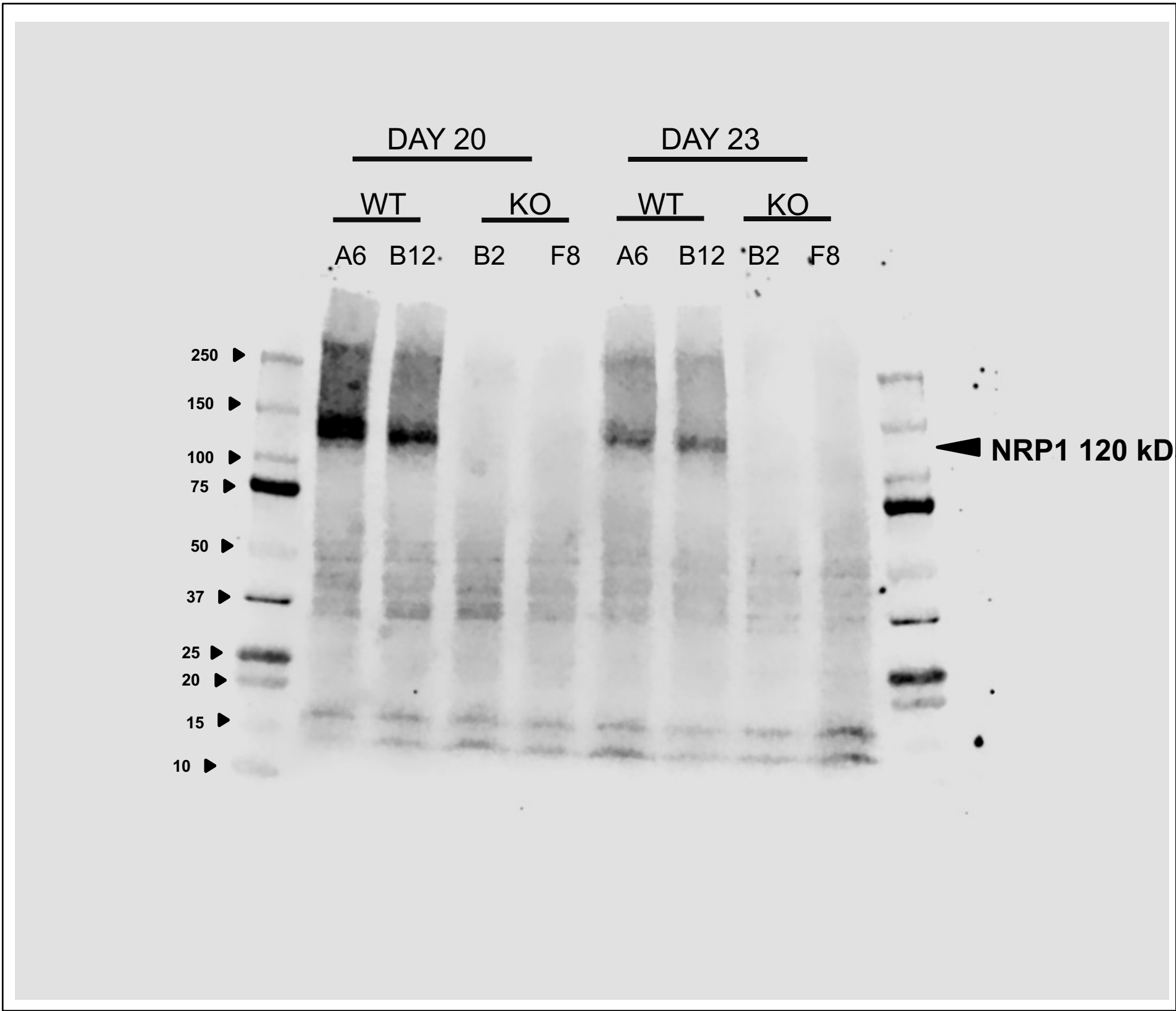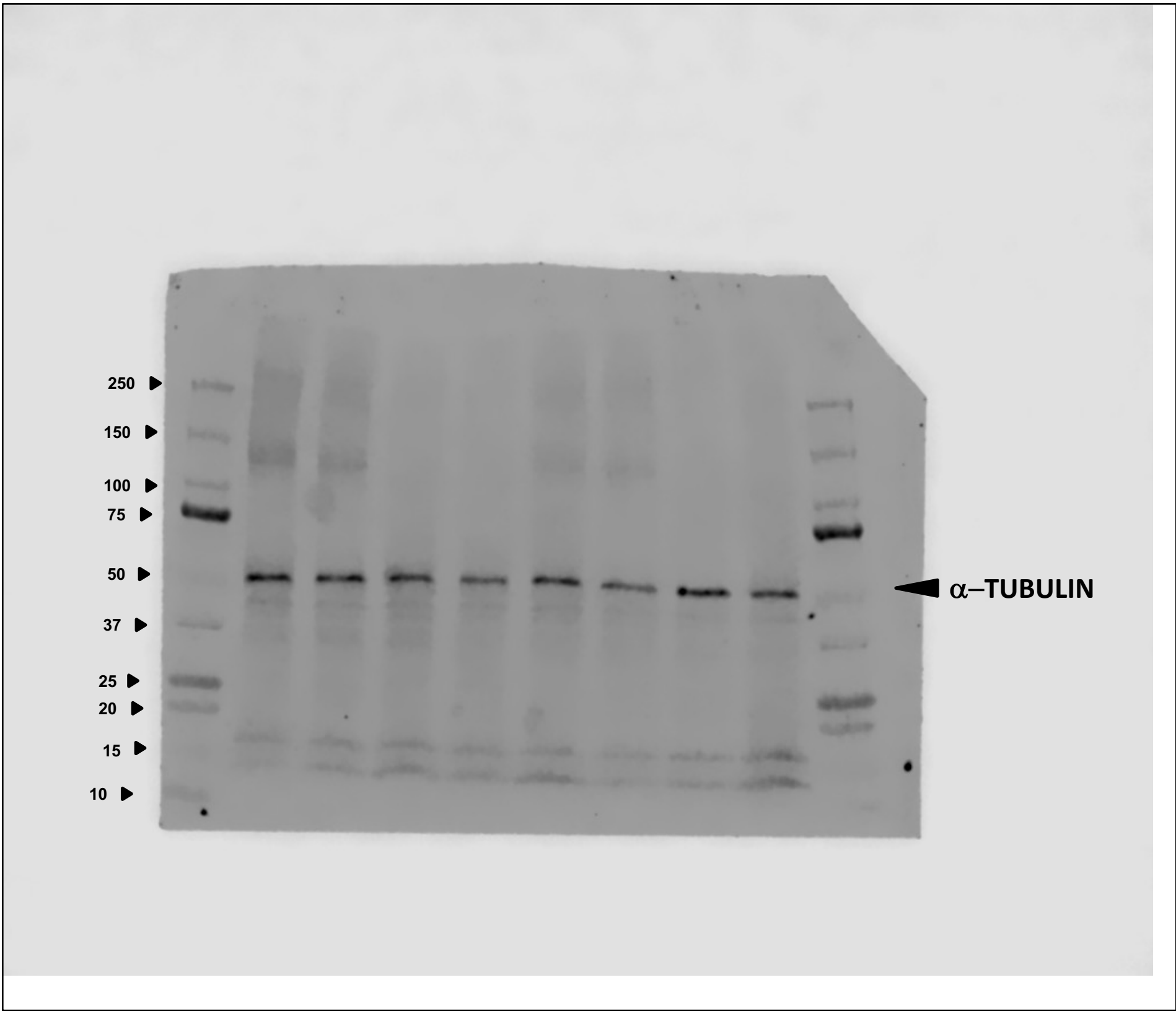

Figure S9D

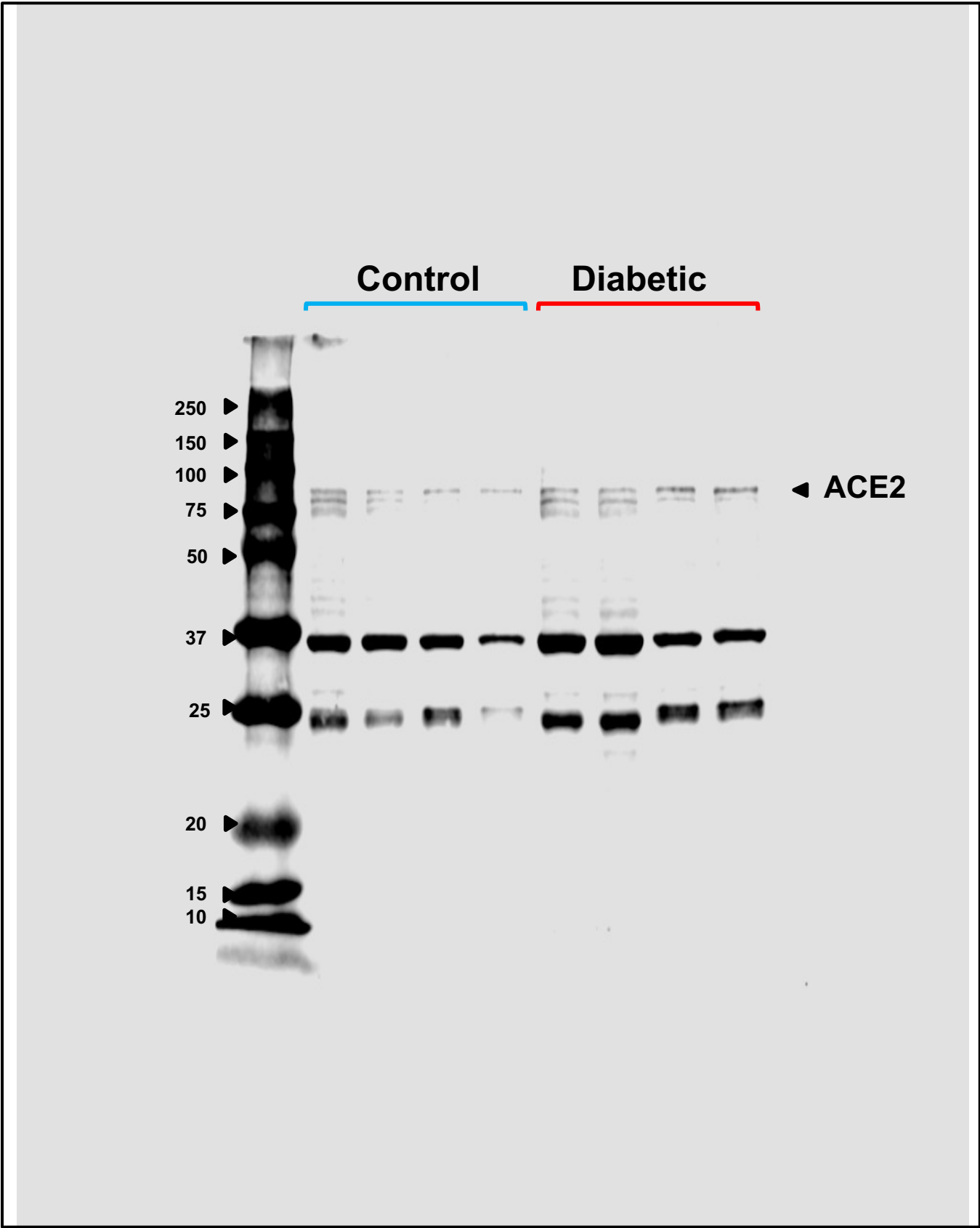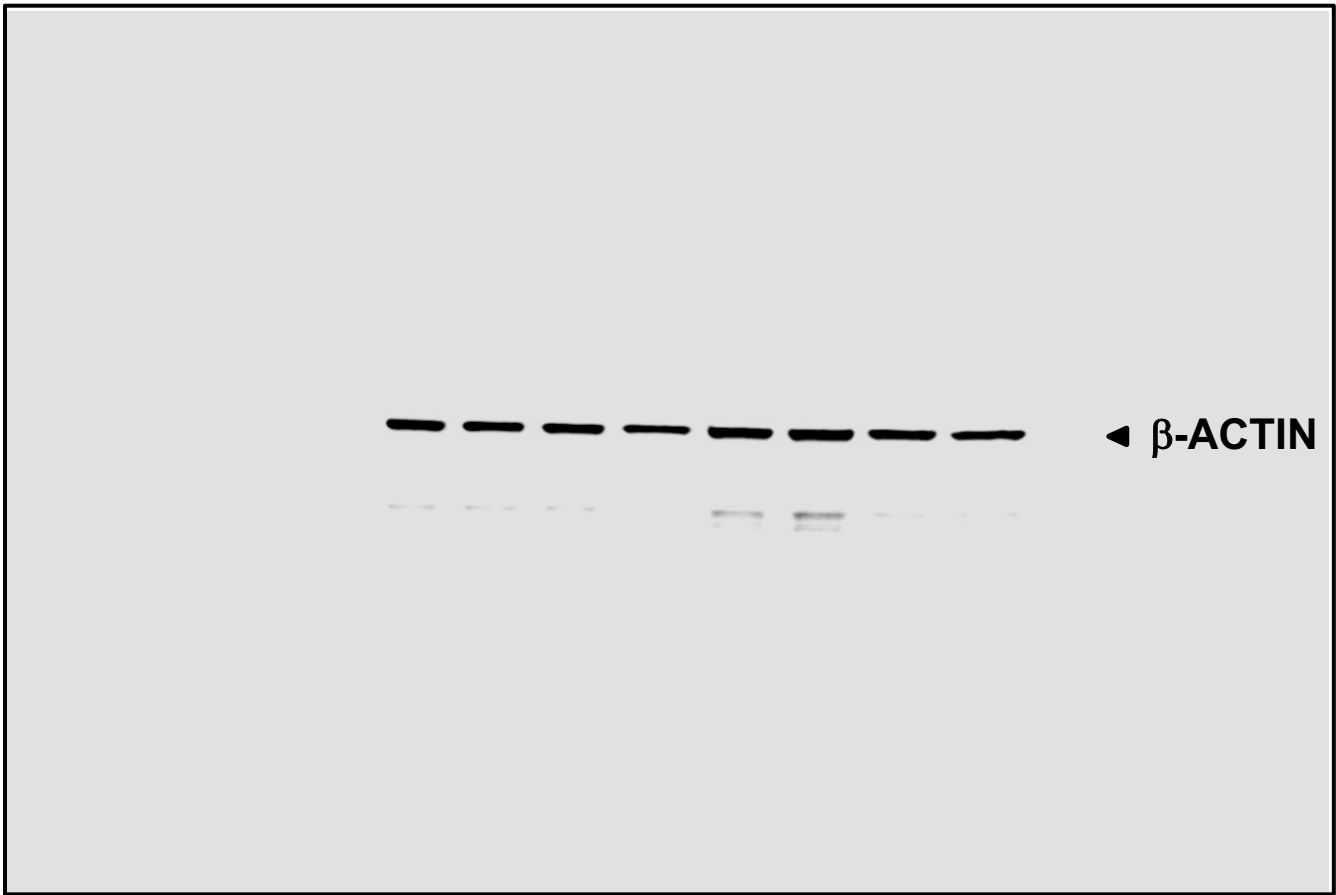

Supplement: Data S1. Unprocessed source data underlying all blots and graphs and supporting data, related to Figures 1–7 and S1–S9 [file mmc2.zip › DataS1 Source Data/Data S1_Western blots.pdf]
