# Supplementary material for: A diabetic milieu increases ACE2 expression and cellular susceptibility to SARS-CoV-2 infections in human kidney organoids and patient cells
Source: Cell Metab. 2022 Jun 7;34(6):857–873.e9. doi: 10.1016/j.cmet.2022.04.009 (PMC9097013; doi:10.1016/j.cmet.2022.04.009)
Supplement: Document S2. Article plus supplemental information [file mmc3.pdf]

Article

Cell Metabolism

A diabetic milieu increases ACE2 expression and cellular susceptibility to SARS-CoV-2 infections in human kidney organoids and patient cells

Graphical abstract

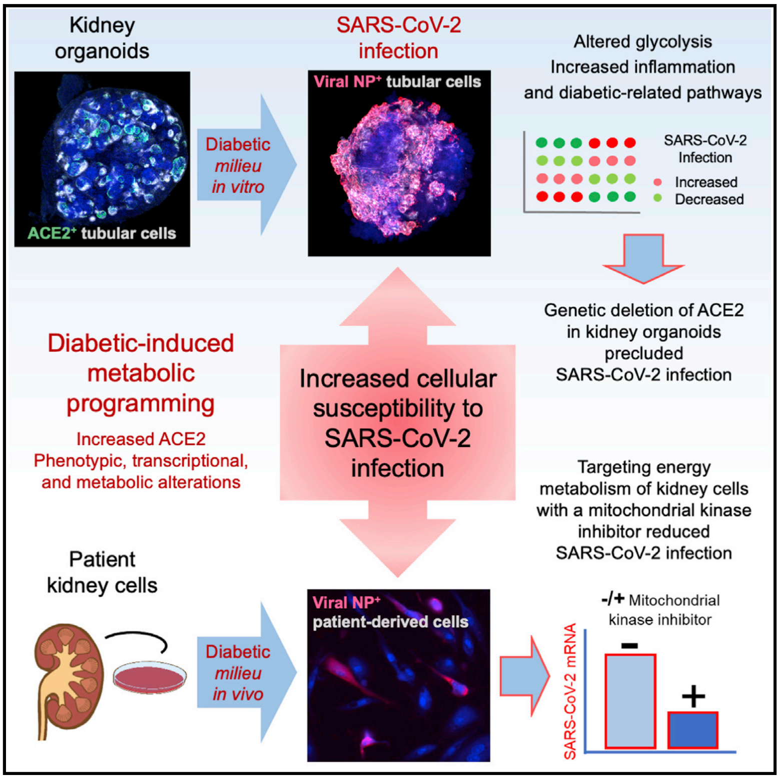

Highlights

- ACE2 is upregulated in hyperglycemia in kidney organoids and patient renal cells
- Metabolic changes and increased ACE2 boost cellular infection by SARS-CoV-2
- Targeting of energy metabolism in patient renal cells lowers SARS-CoV-2 infection

Authors

Elena Garreta, Patricia Prado, Megan L. Stanifer, ..., Steeve Boulant, Josef M. Penninger, Nuria Montserrat

Correspondence

ali.mirazimi@ki.se (A.M.),
s.boulant@ufl.edu (S.B.),
josef.penninger@ubc.ca (J.M.P.),
nmontserrat@ibecbarcelona.eu (N.M.)

In brief

Garreta et al. developed a human kidney organoid system mirroring early hallmarks of diabetic kidney disease development. This resulted in higher SARS-CoV-2 infections, which were critically dependent on ACE2. Diabetic patient renal cells also showed elevated ACE2 and altered energy metabolism that following chemical targeting resulted in a reduction of SARS-CoV-2 infection.

Garreta et al., 2022, Cell Metabolism *34*, 857–873
June 7, 2022 © 2022 The Authors. Published by Elsevier Inc.
https://doi.org/10.1016/j.cmet.2022.04.009

Cell Metabolism

CellPress
OPEN ACCESS

Article

A diabetic milieu increases ACE2 expression and cellular susceptibility to SARS-CoV-2 infections in human kidney organoids and patient cells

Elena Garreta,[1,22] Patricia Prado,[1,22] Megan L. Stanifer,[2,3,4,22] Vanessa Monteil,[5,22] Andrés Marco,[1] Asier Ullate-Agote,[6,7,8] Daniel Moya-Rull,[1] Amaia Vilas-Zornoza,[6,7,8] Carolina Tarantino,[1] Juan Pablo Romero,[6] Gustav Jonsson,[9] Roger Oria,[10] Alexandra Leopoldi,[9] Astrid Hagelkruys,[9] Maria Gallo,[1] Federico González,[1] Pere Domingo-Pedrol,[11] Aleix Gavaldà,[12] Carmen Hurtado del Pozo,[1] Omar Hasan Ali,[13,14,15] Pedro Ventura-Aguiar,[16,17] Josep María Campistol,[16,17] Felipe Prosper,[6,7,8] Ali Mirazimi,[5,18,*] Steeve Boulant,[3,4,19,*] Josef M. Penninger,[9,13,*] and Nuria Montserrat[1,20,21,23,*]

[1]Pluripotency for Organ Regeneration, Institute for Bioengineering of Catalonia (IBEC), The Barcelona Institute of Science and Technology (BIST), Barcelona, Spain
[2]Department of Infectious Diseases, Molecular Virology, Heidelberg University Hospital, Heidelberg, Germany
[3]Research Group "Cellular Polarity and Viral Infection," German Cancer Research Center (DKFZ), Heidelberg, Germany
[4]Department of Molecular Genetics and Microbiology, College of Medicine, University of Florida, Gainesville, FL, USA
[5]Karolinska Institute and Karolinska University Hospital, Unit of Clinical Microbiology, 17182 Stockholm, Sweden
[6]Área de Hemato-Oncología, Centro de Investigación Médica Aplicada, Instituto de Investigación Sanitaria de Navarra (IDISNA), Universidad de Navarra, 31008 Pamplona, Spain
[7]Centro de Investigación Biomédica en Red de Cáncer (CIBERONC), 28029 Madrid, Spain
[8]Departamento de Hematología, Clínica Universidad de Navarra, Universidad de Navarra, 31008 Pamplona, Spain
[9]IMBA, Institute of Molecular Biotechnology of the Austrian Academy of Sciences, Dr. Bohr-Gasse 3, 1030 Vienna, Austria
[10]Center for Bioengineering and Tissue Regeneration, UCSF, San Francisco, CA, USA
[11]Internal Medicine Department, Hospital Universitario de la Santa Creu i Sant Pau, Barcelona, Spain
[12]Departament de Bioquímica i Biomedicina Molecular, Institut de Biomedicina (IBUB), Universitat de Barcelona and CIBER Fisiopatología de la Obesidad y Nutrición, Barcelona, Spain
[13]Department of Medical Genetics, Life Sciences Institute, University of British Columbia, Vancouver, BC, Canada
[14]Department of Dermatology, University Hospital Zurich, University of Zurich, Zurich, Switzerland
[15]Institute of Immunobiology, Cantonal Hospital St. Gallen, St. Gallen, Switzerland
[16]Nephrology and Kidney Transplant Department, Hospital Clínic Barcelona, Barcelona, Spain
[17]Laboratori Experimental de Nefrologia I Trasplantament (LENIT), Fundació Clínic per a la Recerca Biomèdica (FCRB), Barcelona, Spain
[18]National Veterinary Institute, Uppsala, Sweden
[19]Department of Infectious Diseases, Virology, Heidelberg University Hospital, Heidelberg, Germany
[20]Catalan Institution for Research and Advanced Studies (ICREA), Barcelona, Spain
[21]Centro de Investigación Biomédica en Red en Bioingeniería, Biomateriales y Nanomedicina, Madrid, Spain
[22]These authors contributed equally
[23]Lead contact
*Correspondence: ali.mirazimi@ki.se (A.M.), s.boulant@ufl.edu (S.B.), josef.penninger@ubc.ca (J.M.P.), nmontserrat@ibecbarcelona.eu (N.M.)
https://doi.org/10.1016/j.cmet.2022.04.009

SUMMARY

It is not well understood why diabetic individuals are more prone to develop severe COVID-19. To this, we here established a human kidney organoid model promoting early hallmarks of diabetic kidney disease development. Upon SARS-CoV-2 infection, diabetic-like kidney organoids exhibited higher viral loads compared with their control counterparts. Genetic deletion of the angiotensin-converting enzyme 2 (ACE2) in kidney organoids under control or diabetic-like conditions prevented viral detection. Moreover, cells isolated from kidney biopsies from diabetic patients exhibited altered mitochondrial respiration and enhanced glycolysis, resulting in higher SARS-CoV-2 infections compared with non-diabetic cells. Conversely, the exposure of patient cells to dichloroacetate (DCA), an inhibitor of aerobic glycolysis, resulted in reduced SARS-CoV-2 infections. Our results provide insights into the identification of diabetic-induced metabolic programming in the kidney as a critical event increasing SARS-CoV-2 infection susceptibility, opening the door to the identification of new interventions in COVID-19 pathogenesis targeting energy metabolism.

This is an open access article under the CC BY license (http://creativecommons.org/licenses/by/4.0/).

INTRODUCTION

Coronavirus disease 2019 (COVID-19) is an infectious disease caused by severe acute respiratory syndrome coronavirus 2 (SARS-CoV-2). COVID-19 patients display influenza-like symptoms ranging from mild disease to severe lung injury. A high percentage of severe COVID-19 patients display symptoms in other organs, most notably the gastrointestinal tract, cardiovascular system, and the kidney. Several conditions have been linked to the risk of developing severe COVID-19, including genetic predisposition (Ellinghaus et al., 2020; van der Made et al., 2020; Zhang et al., 2020b), immune-related responses (Bastard et al., 2021; Zhang et al., 2020a), obesity, or diabetes mellitus (DM) (Lim et al., 2021). Both COVID-19 and DM are associated with acute and chronic inflammation and both disease conditions can impact each other in terms of clinical progression and disease outcome (Ugwueze et al., 2020). Notably, SARS-CoV-2 infections lead to acute kidney injury (AKI) in >20% of hospitalized patients (Nadim et al., 2020), and higher rates of mortality have been reported in patients with pre-existing DM (Khalili et al., 2021; Legrand et al., 2021).

We have previously shown that both kidney and vascular organoids derived from human pluripotent stem cells (hPSCs) support SARS-CoV-2 infections, which was blocked in the presence of clinical-grade human recombinant soluble angiotensin-converting enzyme 2 (ACE2) (Monteil et al., 2020). However, besides the great utility of organoids in SARS-CoV-2 research, organoids have not yet been developed that model human co-morbidities associated with severe COVID-19, such as DM.

Here, we show that high glucose oscillations in engineered human kidney organoids led to phenotypic, transcriptional, and metabolic alterations reminiscent to human kidney disease development in a diabetic milieu. These diabetic conditions enhanced ACE2 expression and SARS-CoV-2 infection, which was validated in human proximal tubular cells isolated from diabetic kidney biopsies. Genetic deletion of ACE2, but not BSG/CD147 or NRP1, other candidate receptors for SARS-CoV-2, completely abrogated SARS-CoV-2 infections under normal and diabetic conditions. This study provides mechanistic evidence on metabolic alterations that can increase cellular susceptibility to SARS-CoV-2 infections and unequivocally establishes ACE2 as the critical SARS-CoV-2 receptor in the human kidney, even under diabetic conditions.

RESULTS

Establishment of diabetic human kidney organoids

Diabetic patients exhibit oscillatory levels in glucose with daily episodes of hypo- and hyperglycemia, which is thought to be important for driving DM-associated pathologies (Vasquez-Muñoz et al., 2021). Indeed, the term "metabolic memory" coins the pathogenic alterations induced by hyperglycemia long after accomplishment of glycemic control (Genuth et al., 2002; Writing Team for the Diabetes Control and Complications Trial/Epidemiology of Diabetes Interventions and Complications Research Group, 2003). To emulate *in vitro* diabetic-patient-like oscillations in glucose levels, we established a procedure to generate diabetic-like kidney organoids from hPSCs by adapting our previous protocol (Monteil et al., 2020). We delineated a culture setup using continuous low glucose (5 mM, termed "control" conditions) or high glucose in an oscillatory fashion (alternating 5–25 mM every 24 h, termed "diabetic" conditions) (Figure 1A). Kidney organoids under both control and diabetic culture conditions showed the presence of glomerular-like and renal-tubular-like structures to a similar extent, as determined by immunodetection of $PODXL^+$ podocyte-like cells and *Lotus Tetraglobus* lectin proximal tubular-like cells $(LTL)^+$ (Figure S1A) and qPCR analysis for the proximal tubule marker gene *SLC3A1* and the podocyte marker genes *NPHS1*, *PODXL*, *WT1*, and *MAFB* (Figure S1B). Similarly, the mRNA expression levels of the endothelial marker genes *Endoglin*, *VGFR*, and *PDGFRα* and the stromal marker genes *vimentin* and *MEIS1/2/3* did not show significant variations between culture conditions (Figure S1B). Transmission electron microscopy (TEM) showed that tubular-like and podocyte-like cells in both control and diabetic conditions exhibited typical late-stage renal differentiation features (Figure S1C).

Changes in kidney extracellular matrix (ECM) proteins are associated with renal fibrosis and chronic kidney disease (CKD) in diabetes (Bülow and Boor, 2019; Yang et al., 2015). Importantly, high oscillatory glucose treatment resulted in increased collagen fiber deposition in kidney organoids compared with low-glucose controls (Figures 1B–1D; Data S1). We also observed an upregulation of collagen III and collagen IV mRNA expression by qPCR (Figure 1E). Similarly, fibronectin deposits were detected in the tubulointerstitial areas within kidney organoids (Figure 1F; Data S1). PAS staining and immunohistochemistry for the basement membrane proteins laminin and collagen IV in combination with the podocyte marker nephrin and proximal tubule marker LTL showed that the composition and integrity of basement membranes were largely preserved under diabetic conditions (Figures S1D and S1E; Data S1). The glycolysis-associated genes *HK2* and *LDHA* were upregulated in the diabetic kidney organoids (Figure S1F), whereas *PGC1α* (a key regulator of mitochondrial biogenesis involved in diabetic nephropathy) (Li and Susztak, 2018) was downregulated (Figure S1G).

To assess whether the diabetic milieu altered cellular metabolism, we isolated proximal tubular-like cells from kidney organoids by fluorescent-activated cell sorting of the LTL^+ cell fraction (Figures S2A and S2B). LTL^+ cells isolated from both experimental conditions were expanded in 5 mM glucose medium for 2 months until passage 5–7 in culture. After this time, PGC1α expression in LTL^+ cells was significantly decreased in cells isolated from diabetic organoids compared with control organoids as shown by qPCR (Figure S2C) and immunofluorescence (Figure S2D). The expression of the tubular markers LTL and Na-K ATPase was unchanged (Figure S2D). The reduction in *PGC1α* levels was concomitant with increases in the maximum oxygen consumption rate (OCR), a measurement of mitochondrial respiration, basal and maximal respiration, as well ATP synthesis (Figure 1G). Of note, our results are in line with other studies at early phases of diabetes in animal models (1–4 weeks after induction of diabetes) (Friederich et al., 2008; Friederich-Persson and Persson, 2020). In summary, our data show that exposure

CellPress
OPEN ACCESS

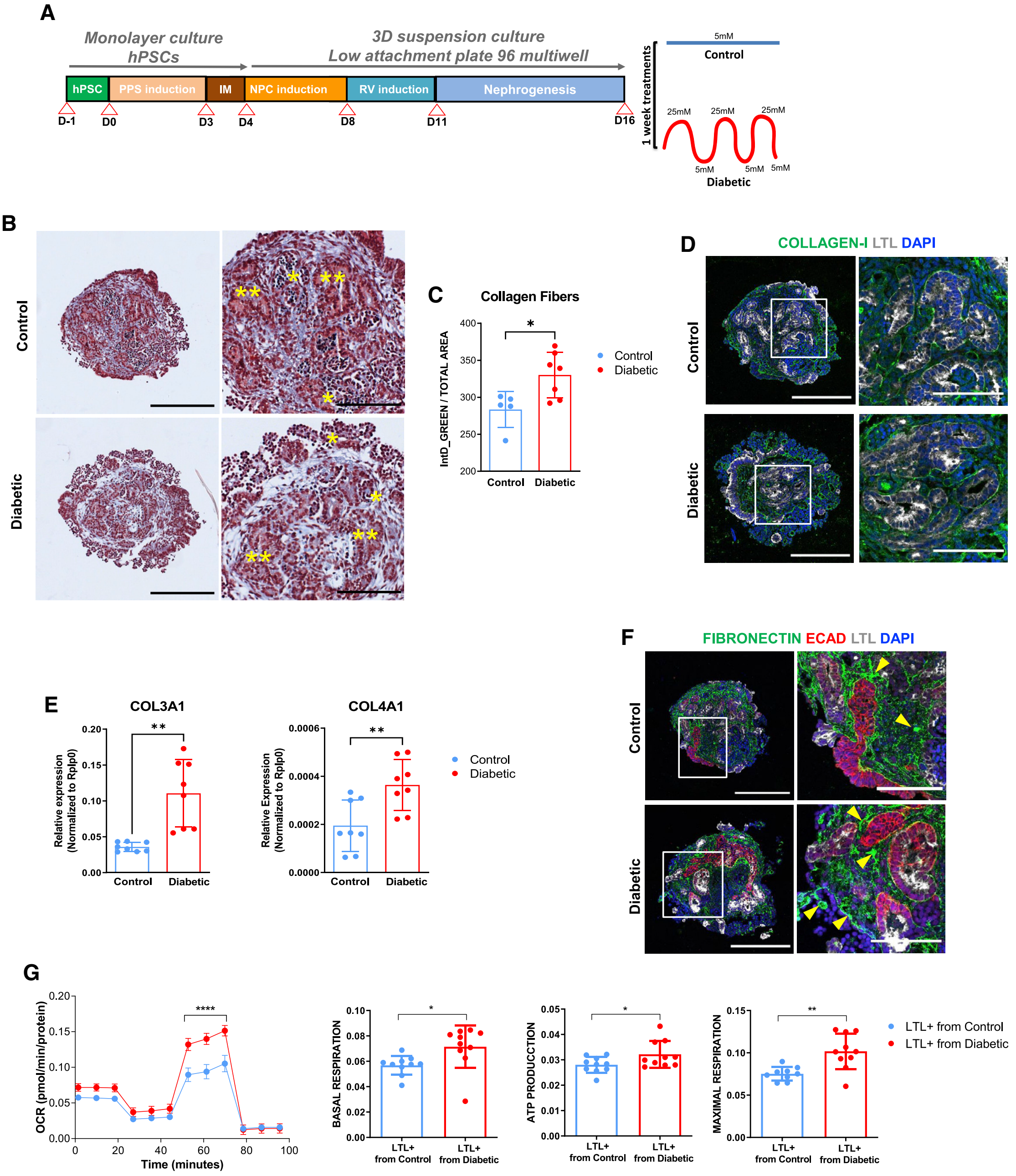

Figure 1. High oscillatory glucose conditions induce early hallmarks of the diabetic kidney disease in human kidney organoids

(A) Experimental scheme for the generation of human kidney organoids from hPSCs.

(B) Trichrome Masson staining of control or diabetic kidney organoids. Glomerular (*) and tubular (**) structures are shown. Scale bars, 250 and 100 μm (magnified views).

(C) Corresponding quantification of collagen fibers (B). y axis represents integrated intensity. Data are mean ± SD of at least n = 5 independent experimental replicates per condition. *p < 0.05, unpaired Student's t test.

(D) Representative immunofluorescence staining for COLLAGEN-I (green), LTL (gray), and DAPI (blue) in control or diabetic kidney organoids. Scale bars, 250 and 100 μm (magnified views).

(E) mRNA expression levels of *COL3A1* and *COL4A1* in control or diabetic kidney organoids. Data are mean ± SD. n = 3 independent biological replicates from a pool of 12 organoids/group with two technical replicates each. **p < 0.01, unpaired Student's t test.

(F) Representative immunofluorescence staining for FIBRONECTIN (green), E-CADHERIN (ECAD; red), LTL (gray), and DAPI (blue) in control or diabetic kidney organoids. Scale bars, 250 and 100 μm (magnified views). Yellow arrows highlight sites of fibronectin deposits.

(G) Seahorse analysis in LTL^+ cells isolated from control or diabetic kidney organoids. The oxygen consumption rate (OCR) data are normalized to total protein. Data are mean ± SD. n = 10 biological replicates/group. ****p < 0.0001, two-way ANOVA, followed by Bonferroni post-test. Basal respiration and spare respiratory capacity, cellular ATP production, and maximal respiration are shown as mean ± SD. n = 10 biological replicates/group. *p < 0.05; **p < 0.005, unpaired Student's t test. See also Figures S1 and S2 and Data S1.

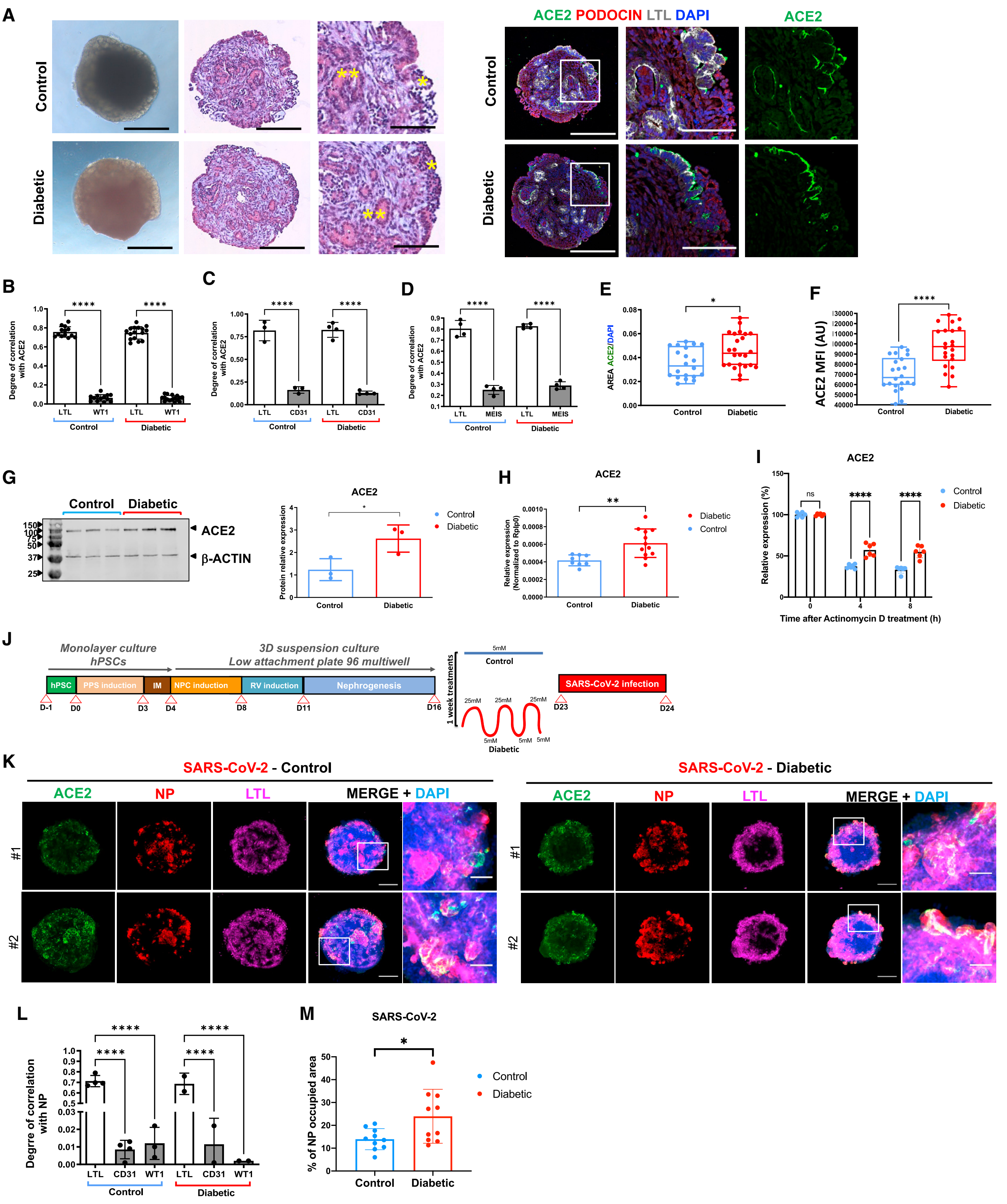

Figure 2. Diabetic conditions induce ACE2 expression in human kidney organoids and enhance SARS-CoV-2 infections

(A) Representative bright-field images and hematoxylin and eosin staining of control or diabetic kidney organoids. Asterisks highlight podocyte-like cells (*) or tubular-like structures (**). Consecutive sections were stained for ACE2 (green), PODOCIN (red), and using *Lotus Tetraglobus* lectin (LTL, tubular cell marker, gray) and DAPI (blue). Scale bars, 250 and 100 μm (magnified views).

(legend continued on next page)

of human kidney organoids to high oscillatory glucose leads to transcriptional changes, ECM alterations, and metabolic mitochondrial rewiring in tubular cells, early hallmarks of kidney disease development induced by hyperglycemia. Our culture system also allowed for the generation of proximal tubule-like cells (LTL^+) recapitulating hallmarks of an early diabetogenic-like phenotype after removal of the diabetogenic insult, indicative of metabolic memory.

Diabetic conditions induce ACE2 expression in human kidney organoids

ACE2 has been previously identified as a key host cell surface receptor sufficient for SARS-CoV-2 entry into host cells (Walls et al., 2020; Wan et al., 2020; Wrapp et al., 2020). Of note, ACE2 has also been previously shown to control the progression of CKD in multiple animal models (Maksimowski et al., 2020). Although it has been reported that glucose can induce ACE2 expression in cell lines (Härdtner et al., 2013), it is still controversial whether DM results in up- or downregulation of ACE2 (Mizuiri et al., 2008; Reich et al., 2008; Sandooja et al., 2020), making it paramount to test the effects of glucose on ACE2 expression in complex human tissue-like cultures as organoids. Our analysis indicated that in the kidney organoids, ACE2-expressing cells (ACE2+) are predominantly detected in LTL^+ (Figure 2A; Data S1). Image quantification of $ACE2^+LTL^+$ was evaluated together with markers of different renal compartments including the podocyte marker WT1, the endothelial cell marker CD31, and the stroma marker MEIS1/2/3 (Figures 2B–2D; Data S1), overall showing a similar expression pattern of ACE2 to that found in the native human and mouse kidney (Deng et al., 2020; Lin et al., 2021). Importantly, the oscillatory glucose treatment promoted a significant upregulation of ACE2 expression compared with control conditions at the protein (Figures 2E–2G and S2E–S2G) and mRNA (Figure 2H) levels. Since mRNA stability plays a major role in gene expression regulation, we hypothesized that hyperglycemia might affect the half-life of *ACE2* mRNA. To test this idea, kidney organoids were challenged with the RNA transcription inhibitor actinomycin D leading to an increase of *ACE2* mRNA stability in diabetic organoids compared with control counterparts (Figure 2I).

Increased SARS-CoV-2 infection in diabetic kidney organoids

Next, control and diabetic kidney organoids were infected with SARS-CoV-2, recovered, and analyzed at 1 day post-infection (1 dpi) (Figure 2J). Control organoids were productively infected as detected by immunostaining for the viral nuclear protein (NP) (Figure 2K). Infected cells within organoids were primarily LTL^+ (Figure 2L; Data S1). Remarkably, diabetic organoids showed significantly enhanced SARS-CoV-2 infections compared with control organoids, as quantified by the percentage of cells positive for NP expression (NP^+) by confocal microscopy (Figure 2M; Data S1). To assess potential transcriptional changes induced by SARS-CoV-2, single-cell RNA sequencing (scRNA-seq) was performed across 4 biological conditions (mock and 1 dpi, in both 5 mM glucose- and high oscillatory glucose-treated kidney organoids). Cell types in kidney organoids were assigned using unsupervised clustering after integrating control versus diabetic infected conditions and the uniform manifold approximation and projection (UMAP) algorithm to visualize the scRNA-seq data (Figure 3A). We retrieved renal endothelial-like, mesenchymal, proliferating, podocyte, and tubule cell populations in all four experimental samples, indicating that apparently neither diabetic conditions nor SARS-CoV-2 infections altered cell compositions in the kidney organoids (Figure S3A). Organoids from the all four conditions contained cells representative of a developing nephron, including ENG^+ and $PECAM1^+$ (endothelial-like cells), $NPHS1^+$ and $NPHS2^+$ (podocyte-like cells), $LRP2^+$ and $SLC3A1^+$ (tubule proximal-like cells), and $CDH1^+$ (distal-like tubular cells) (Figure S3B), also resembling second trimester human fetal kidney cell populations (Figure S3C). Diabetic kidney

(B) Quantification of ACE2 colocalization with LTL^+ and $WT1^+$ cells in control or diabetic kidney organoids. Data are mean ± SD. n = 13 (control) and n = 16 (diabetic) organoids per condition. ****$p < 0.0001$, one-way ANOVA, Tukey's multiple comparisons test.
(C) Quantification of ACE2 colocalization with LTL^+ and $CD31^+$ cells in control or diabetic kidney organoids. Data are mean ± SD. n = 3 (control) and n = 4 (diabetic) organoids per condition. ****$p < 0.0001$, one-way ANOVA, Tukey's multiple comparisons test.
(D) Quantification of ACE2 colocalization with LTL^+ and $MEIS^+$ cells in control or diabetic kidney organoids. Data are mean ± SD. n = 4 (control) and n = 4 (diabetic) organoids per condition. ****$p < 0.0001$, one-way ANOVA, Tukey's multiple comparisons test.
(E) Quantification of the area of $ACE2^+$ cells in control or diabetic kidney organoids. Data are mean ± SD. n = 20 (control) and n = 24 (diabetic) organoids. *$p < 0.05$, unpaired Student's t test.
(F) Quantification of the mean fluorescence intensity (MFI as arbitrary units [AU]) of $ACE2^+$ cells in control or diabetic kidney organoids. Data are mean ± SD. n = 6 (control) and n = 6 (diabetic) organoids. ****$p < 0.0001$, unpaired Student's t test.
(G) Protein levels of ACE2 in control or diabetic kidney organoids are shown by western blot. β-actin was used as loading control. The correspondent quantification is shown. Data are mean ± SD. n = 3 independent biological replicates from a pool of 12 organoids/group; *$p < 0.05$, unpaired Student's t test.
(H) mRNA expression levels of *ACE2* in control or diabetic kidney organoids. Data are mean ± SD. n = 4 independent experimental replicates from a pool of 12 organoids/group with two technical replicates each. **$p < 0.005$, unpaired Student's t test.
(I) mRNA expression levels of *ACE2* in control or diabetic kidney organoids upon actinomycin D treatment. Data are mean ± SD. n = 2 independent biological replicates from a pool of 12 organoids/group with three technical replicates each. ns, no statistical significance; ****$p < 0.0001$, two-way ANOVA followed by Bonferroni post-test.
(J) Experimental scheme for the infection of control or diabetic kidney organoids with SARS-CoV-2.
(K) Immunofluorescence of control and diabetic kidney organoids at 1 dpi with SARS-CoV-2 for ACE2 (green), viral nuclear protein (NP, red), LTL (magenta), and DAPI (blue). Scale bars, 250 and 50 μm (magnified views). n = 2 organoids per condition.
(L) Quantification of viral NP colocalization with LTL^+, $CD31^+$, and $WT1^+$ cells in control or diabetic kidney organoids. Data are mean ± SD. n = 3 control and n = 3 diabetic organoids. ****$p < 0.0001$, one-way ANOVA, Tukey's multiple comparisons test.
(M) Quantification of the area of NP^+ cells from images in (K). Data are mean ± SD. n = 5 control and n = 5 diabetic kidney organoids performing two technical replicates each. *$p < 0.05$, unpaired Student's t test.
See also Figure S2 and Data S1.

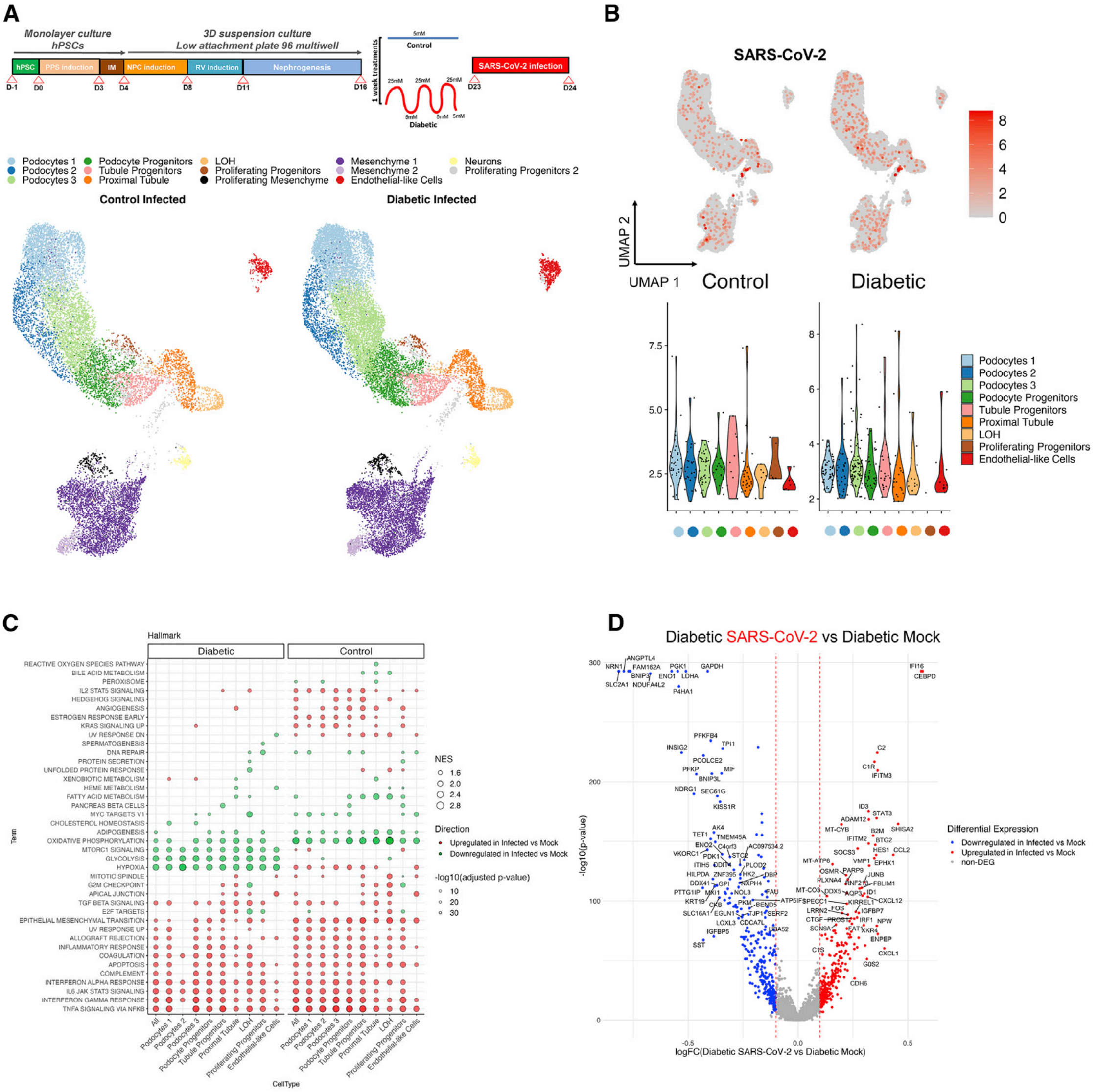

Figure 3. Diabetic-induced metabolic programming in human kidney organoids enhances SARS-CoV-2 infection

(A) Uniform manifold approximation and projection (UMAP) of control or diabetic kidney organoids at 1 dpi with SARS-CoV-2. Clusters are colored by annotated cell types.

(B) UMAPs for SARS-CoV-2 expression in control or diabetic kidney organoids at 1 dpi. For SARS-CoV-2, expression is considered as undetectable for cells expressing <5 unique molecular identifiers (UMIs). Cells are colored based on expression level. The violin plots in the bottom panels represent ≥ 5 UMI expression levels for the different cell types indicated.

(C) A hallmark GSEA was performed separately for control and diabetic conditions, comparing SARS-CoV-2-infected versus mock organoids. The ten gene sets per direction and sample with lowest adjusted p values ($p < 0.05$) are shown. Each column corresponds to one of the comparisons. Circles are coded by color (direction), size (NES), and transparency ($-\log_{10}$(p value)).

(D) Differentially expressed genes (DEGs) in the comparison of the SARS-CoV-2-infected against mock organoids in diabetic conditions considering only renal-like cell types. In the volcano plot, the x axis indicates log fold change (FC), and the y axis indicates statistical significance with the $-\log_{10}$(p value). Genes with an adjusted p value < 0.05 are considered upregulated (red) if the logFC > 0.1 and downregulated (blue) if the logFC < $-$0.1. Non-DEGs are shown in gray.

See also Figures S3 and S4.

organoids at 1 dpi again showed increased numbers of cells containing viral RNA compared with control organoids (Figure 3B). As expected from an active infection, SARS-CoV-2 infections resulted in an increase of inflammatory-related processes in kidney organoids under control and diabetic conditions (Figure 3C) in agreement with previous findings in kidney organoids showing increased profibrotic signaling, cellular injury, and inflammatory responses driven by SARS-CoV-2 infection (Jansen et al., 2021). Our results also showed that SARS-CoV-2 infections were also associated with downregulation of glycolysis-related processes in diabetic organoids (Figure 3C). In addition to altered glycolysis, differential gene expression analysis showed increased inflammation (e.g., *CXCL* family genes) and diabetic-related pathways (e.g., *CEBPD* and *STAT3*)

CellPress
OPEN ACCESS

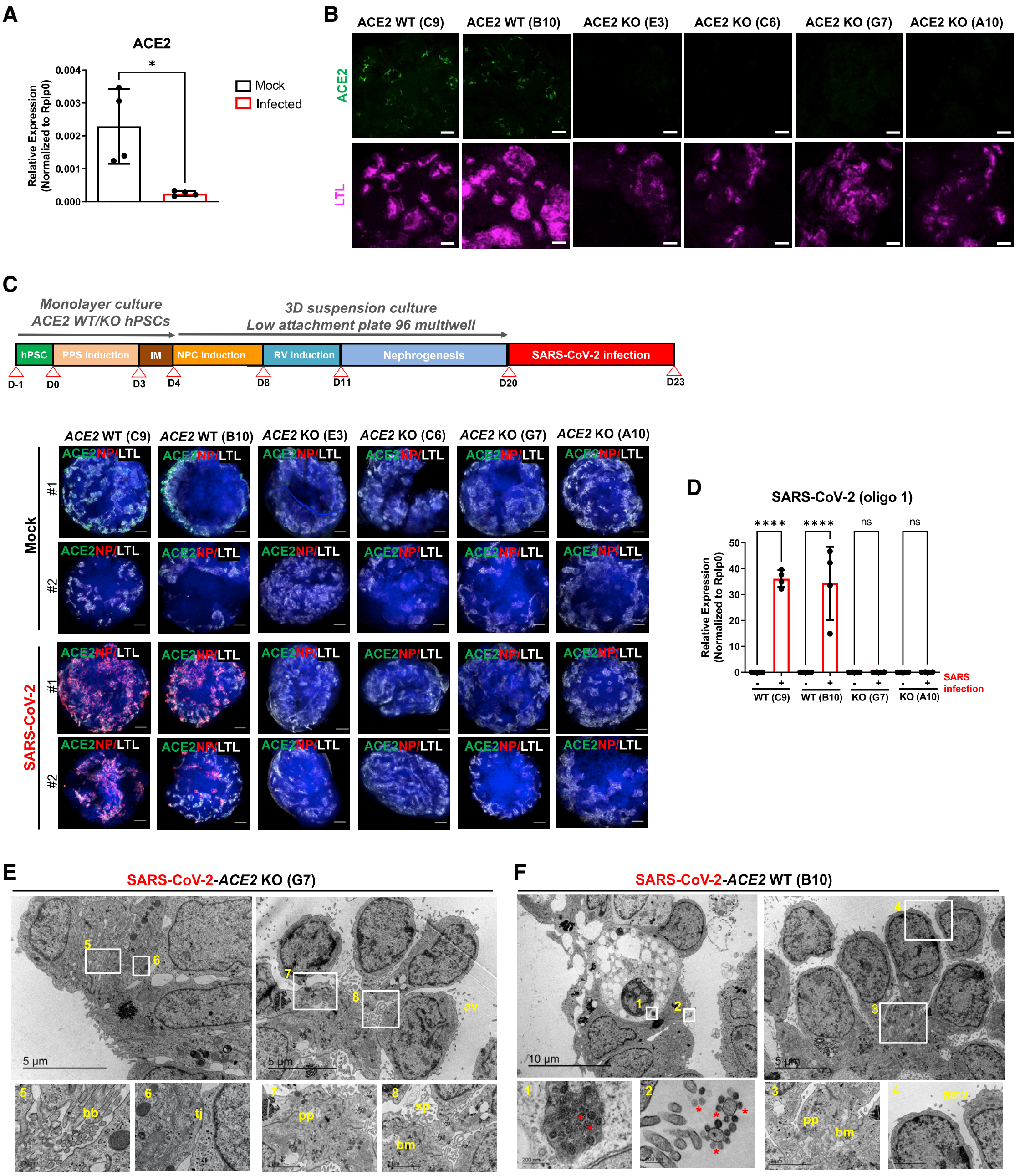

Figure 4. SARS-CoV-2 infection in human kidney organoids depends on ACE2

(A) mRNA expression levels of *ACE2* in mock or SARS-CoV-2-infected kidney organoids. Data are mean ± SD. n = 2 independent biological replicates from a pool of 12 organoids/group with two technical replicates each. $*p < 0.05$, unpaired Student's t test.

(B) Immunofluorescence staining for ACE2 (green) and LTL (magenta) in WT and *ACE2* KO kidney organoids. Scale bars, 50 μm.

(C) Experimental scheme for the infection with SARS-CoV-2 of WT and *ACE2* KO kidney organoids. Immunofluorescence of mock or SARS-CoV-2-infected WT and *ACE2* KO kidney organoids at 3 dpi for ACE2 (green), viral nuclear protein (NP, red), LTL (gray), and DAPI (blue). Scale bars, 250 μm.

(D) mRNA expression of SARS-CoV-2 and *ACE2* in mock or SARS-CoV-2-infected WT and *ACE2* KO kidney organoids at 3 dpi by qPCR. Data are mean ± SD. n = 2 independent biological replicates from a pool of 12 organoids/group with two technical replicates each. $****p < 0.0001$; ns, no statistical significance; one-way ANOVA, Tukey's multiple comparisons test.

(E) TEM analysis of WT and *ACE2* KO kidney organoids infected with SARS-CoV-2 and recovered at 3 dpi. Representative images of infected *ACE2* WT specimen show numerous viral particles (asterisks) inside a vesicle near the plasma membrane of a dying cell (1) and in the apical microvilli (amv) of a tubular-like cell (2). Details for podocyte-like cells exhibiting podocyte-related structures including primary processes (pp) (3), the deposition of a basement membrane (bm) (3),

(legend continued on next page)

in SARS-CoV-2-infected diabetic kidney organoids compared with non-diabetic counterparts (Figures 3D and S3D–S3F).

Recent findings showed that glycolysis sustains SARS-CoV-2 replication in human monocytes exposed to high glucose (11 mM) (Codo et al., 2020). Considering our findings that SARS-CoV-2 infection in diabetic kidney organoids led to a decrease in glycolysis, we also investigated the impact of SARS-CoV-2 infection in kidney organoids exposed to 11 mM glucose culture conditions. SARS-CoV-2 infections did not alter cell clustering or cell-type proportions across mock and 1 dpi kidney organoids grown in 11 mM glucose (Figures S4A and S4B). Moreover, cells presenting high expression of viral RNA were mainly located in the loop of Henle and proximal tubule cell clusters, again co-expressing ACE2 (Figure S4C). Importantly, following SARS-CoV-2 infections, we observed a switch from oxidative phosphorylation (OXPHOS) to a glycolytic-based metabolism in the kidney organoids exposed to 11 mM glucose in agreement with recent findings in human monocytes (Figure S4D) (Codo et al., 2020). Analysis for differentially expressed genes confirmed those findings together with enrichment in IFN and TNF, IL-2/6, and mTORC1 signaling (Figures S4D–S4F).

ACE2 is essential for SARS-CoV-2 infections in kidney organoids

The ACE2 receptor is sufficient for SARS-CoV-2 entry into human cells, though other receptors have been proposed (Hoffmann et al., 2020). Whether ACE2 is essential for SARS-CoV-2 infections is still not known. We and others have previously shown that ACE2 expression protects the lung from injury and that its expression is downregulated *in vivo* and *in vitro* upon SARS-CoV and SARS-CoV-2 spike protein engagement on the cell membrane (Haga et al., 2008; Imai et al., 2005; Kuba et al., 2005; Triana et al., 2021). Here, we found that *ACE2* mRNA levels were significantly downregulated in kidney organoids after SARS-CoV-2 infection compared with mock conditions (Figure 4A).

To test whether ACE2 is indeed essential for SARS-CoV-2 infections in kidney organoids, we generated *ACE2* knockout (KO) hPSC lines using CRISPR-Cas9 genome editing (Figures S5A and S5B). Upon differentiation, H&E staining showed that both wild-type (WT) and *ACE2* KO kidney organoids exhibited similar nephron-like structures containing podocyte-like and tubule proximal-like cells (Figure S5C). ACE2 deficiency was confirmed by immunofluorescence (Figure 4B) and western blotting (Figure S5D). qPCR analysis of WT and *ACE2* KO kidney organoids showed the detection of prototypic renal markers (Figure S5E) with no apparent changes in renal cell populations as revealed by scRNA-seq (Figures S6A and S6B). In addition, the expression of basigin (BSG, also known as CD147), proposed as another putative receptor for SARS-CoV-2 (Wang et al., 2020), was not affected by ACE2 deficiency as shown by immunofluorescence (Figure S6C). Interestingly, *ACE2* KO kidney organoids exhibited an upregulation in OXPHOS processes compared with WT organoids as shown by gene set enrichment analysis (GSEA) (Figure S6D). These results are in line with previous observations that genetic deletion of ACE2 or pharmacological inhibition of ACE2 induces renal oxidative stress and promotes diabetic renal injury (Soler et al., 2007; Wysocki et al., 2014).

Next, control WT and *ACE2* KO kidney organoids were infected with SARS-CoV-2 and virus infection was monitored at 3 dpi (Figure 4C). Downregulation of ACE2 expression upon infection in WT organoids was re-confirmed at the protein level (Figure S6E). SARS-CoV-2 infection in *ACE2* WT organoids was shown based in viral NP detection in LTL$^+$ cells by immunofluorescence (Figure 4C) and the expression of SARS-CoV-2 mRNA by qPCR (Figures 4D and S7). In contrast, ACE2 deletion in *ACE2* KO kidney organoids resulted in a complete absence of NP$^+$ cells and almost undetectable levels of SARS-CoV-2 mRNA (Figures 4C, 4D, and S7). TEM analysis corroborated the absence of SARS-CoV-2 viral particles in *ACE2* KO kidney organoids (Figure 4E), whereas viral particles were present in the WT background (Figure 4F). No significant changes in the levels of expression of podocyte marker genes (*WT1*, *PODXL*, *NPHS1*, *NPHS2*, and *MAFB*) or for tubular marker genes (*SLC3A1* and *SLC16A1*) were found after infection comparing WT and *ACE2* KO kidney organoids (Figure S7).

Impact of SARS-CoV-2 infections in *BSG* and *NRP1* KO kidney organoids

To further validate the unique role of ACE2 in SARS-CoV-2 infection in kidney organoids, we generated *BSG* KO hPSCs using CRISPR-Cas9 (Figures 5A and 5B). Upon differentiation, both the respective WT and *BSG* KO kidney organoids exhibited the presence of nephron-like structures containing podocyte-like cells and tubular structures (Figure 5C). BSG deletion in *BSG* KO kidney organoids was confirmed using qPCR (Figure 5D). We also generated *NRP1* KO hPSCs (Figures S8A and S8B), confirming NRP1 deletion in *NRP1* KO kidney organoids by western blotting (Figure S8C). To determine if BSG or NRP1 also plays a role in SARS-CoV-2 infection, as previously proposed (Cantuti-Castelvetri et al., 2020; Daly et al., 2020; Wang et al., 2020), WT and KO kidney organoids for *BSG* and *NRP1* were infected with SARS-CoV-2 and analyzed at 3 dpi (Figures 5E and S8D, respectively). TEM analysis revealed the presence of viral particles in both WT and *BSG* KO organoids (Figure 5F). These results were confirmed by immunofluorescence for the detection of NP$^+$ (Figure 5G). Interestingly, we found decreased levels of SARS-CoV-2 mRNA expression in the infected *BSG* KO kidney organoids compared with WT counterparts (Figure 5H), suggesting that BSG deletion might have an impact on the infection susceptibility of the organoids. In contrast, the presence of NP$^+$ and SARS-CoV-2 mRNA expression levels were comparable between WT and *NRP1* KO kidney organoids upon infection (Figures S8E and S8F). Overall, these findings do not support an indispensable role for BSG or NRP1 in SARS-CoV-2 infections in human kidney organoids.

and apical microvilli (amv) (4) are shown. Scale bars, 10 and 5 μm, 200 nm (magnified views in 1 and 2), and 2 μm (magnified views in 3 and 4). Representative images of infected *ACE2* KO specimen show epithelial tubular-like cells with brush borders (bb) (5) and tight junctions (tj) (6). Details for podocyte-like cells with podocyte-related structures including primary (pp), the deposition of a basement membrane (bm), and cell processes (sp) are shown. Scale bars, 5 μm, 500 nm (magnified views in 5 and 6), and 2 μm (magnified views in 7 and 8).
See also Figures S5–S7.

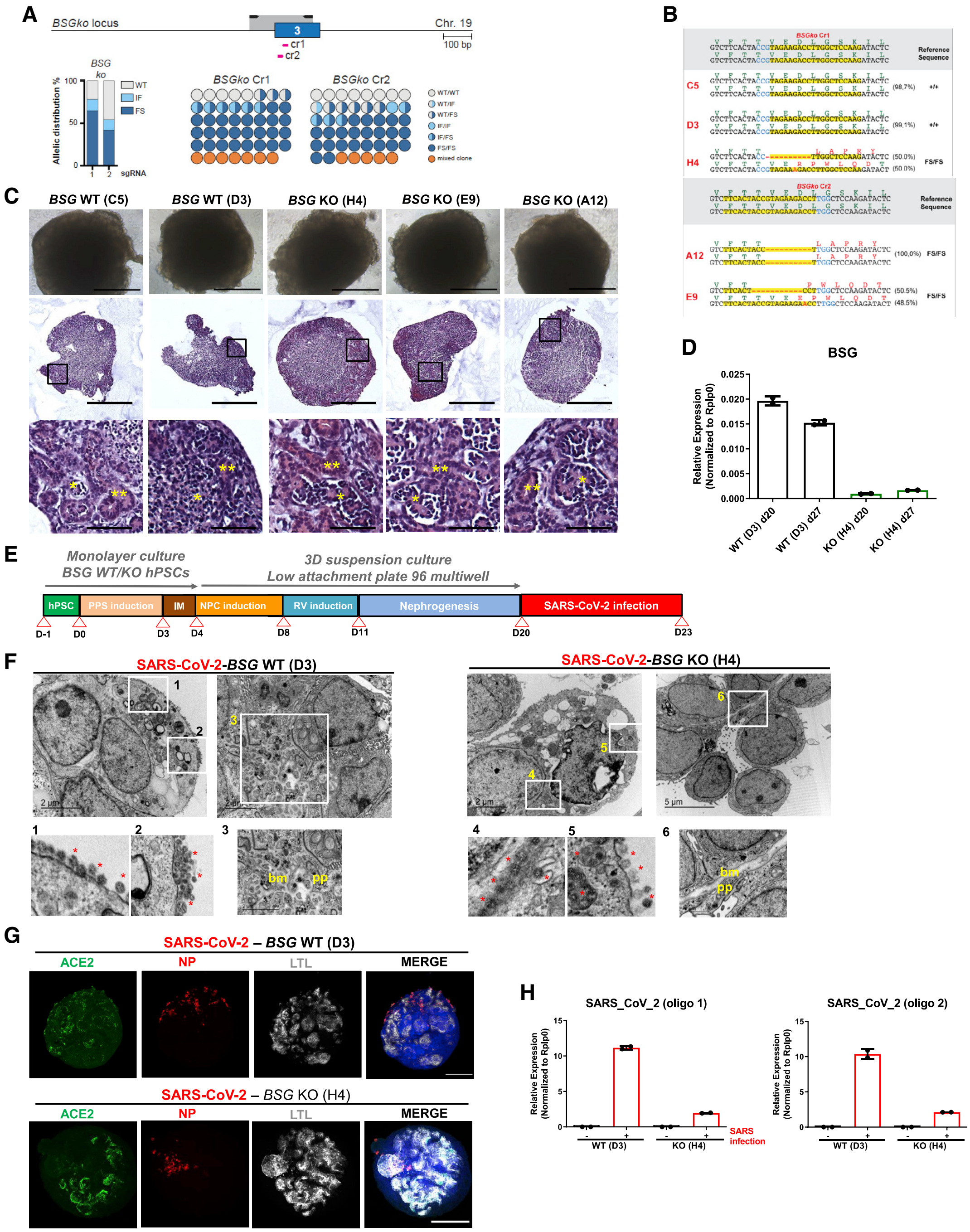

Figure 5. SARS-CoV-2 infections in *BSG* KO kidney organoids

(A) Schematic of Cas9/gRNA-targeting sites (pink arrows) in *BSG locus* showing exon structure (blue boxes) and PCR amplicons (light gray boxes in all figures in this study). Histogram shows allelic sequence distribution after the transfection of the different gRNAs in undifferentiated ES[4] cells expressing an inducible Cas9 (iCas9). WT, wild type; mut, mutation; FS, frameshift.

(B) Representative sequence of the wild type (+/+) or BSG mutant clones generated with the different gRNAs.

(C) Representative bright-field images of WT and *BSG* KO kidney organoids. Scale bars, 250 μm. Hematoxylin and eosin staining show tubular-like (**) and glomerular-like (*) structures. Scale bars, 250 and 50 μm (magnified views).

(D) mRNA expression levels of *BSG* in WT and *BSG* KO kidney organoids by qPCR. Data are mean ± SD. n = 1 independent experiment from a pool of 12 organoids/group with at least two technical replicates each.

(E) Experimental scheme for the infection with SARS-CoV-2 of WT and *BSG* KO kidney organoids.

(F) TEM analysis of WT and *BSG* KO kidney organoids infected with SARS-CoV-2 at 3 dpi. Representative images of infected *BSG* WT specimens (left) show numerous viral particles in the cell surface of a dying cell (1 and 2). Details for podocyte-like cells (3) exhibiting podocyte-related structures including primary (pp)

(legend continued on next page)

ACE2 is essential for SARS-CoV-2 infections in diabetic kidney organoids

We next assessed whether ACE2 is essential for the observed increase in SARS-CoV-2 infections under our diabetic culture conditions. To address this question, WT and *ACE2* KO kidney organoids were exposed to control and diabetic conditions and analyzed at 1 dpi (Figure 6A). Importantly, infected *ACE2* KO kidney organoids were negative for NP detection irrespective of the control or diabetic treatment. On the contrary, *ACE2* WT kidney organoids again displayed significantly enhanced viral NP expression within the LTL$^+$ structures under high oscillatory glucose conditions (Figure 6B) as confirmed by qPCR analysis (Figure 6C). Similarly, TEM analysis also confirmed the presence of viral particles in *ACE2* WT kidney organoids compared with *ACE2* KO counterparts (Data S1). As expected, ACE2 mRNA and protein levels remained undetectable in the diabetic *ACE2* KO kidney organoids (Figures 6D and 6E). To further explore the role of ACE2 in SARS-CoV-2 infections, we tested whether restoration of ACE2 expression in the *ACE2* KO kidney organoids through lentiviral transduction was sufficient to rescue SARS-CoV-2 infectivity in control and diabetic culture conditions. ACE2 transduced (ACE2t) organoids were subsequently infected with SARS-CoV-2 (Figure 6F). At 1 dpi, ACE2t kidney organoids showed similar levels of ACE2 expression (Figures 6F and 6G), and we were able to detect NP$^+$ (Figure 6F). Quantification of NP$^+$ in confocal images revealed that organoids exposed to diabetic conditions showed a significant increase of NP$^+$ compared with their control counterparts (Figure 6H). Thus, diabetic conditions sustain enhanced SARS-CoV-2 infections in kidney organoids, which is critically dependent on ACE2 expression.

Enhanced SARS-CoV-2 infection in kidney cells from diabetic patients

Although several studies could not detect direct SARS-CoV-2 infection in kidneys (Golmai et al., 2020; el Jamal et al., 2021; Santoriello et al., 2020; Volbeda et al., 2021), other recent investigations have clearly shown that SARS-CoV-2 can directly infect renal cells (Braun et al., 2020; Diao et al., 2021; Jansen et al., 2021; Puelles et al., 2020). However, whether the kidney damage observed results from direct infection of target renal cells or indirect injury responses is not yet fully understood (Braun et al., 2020; el Jamal et al., 2021; Puelles et al., 2020; Volbeda et al., 2021). We therefore isolated kidney human proximal tubular cells (HPTCs) from kidney biopsies of non-diabetic (control) and diabetic patients (Figures 7A and S9A). The mRNA levels of *PGC1*α were significantly downregulated in diabetic HPTCs compared with control counterparts (Figure S9B), paralleling our findings in diabetic kidney organoids and in diabetic kidney organoid-isolated LTL$^+$ (Figures S1G and S2C). In addition, diabetic HPTCs exhibited higher OCR, basal respiration, ATP production, and maximal respiratory capacity compared with control counterparts (Figure 7B), as well as increased *LDHA* mRNA levels (Figure S9C). Of note, diabetic HPTCs showed higher ACE2 expression levels compared with control HPTCs (Figures S9D and S9E). Upon SARS-CoV-2 infection, we detected increased numbers of NP$^+$ cells (Figures 7C and 7D) and elevated SARS-CoV-2 mRNA expression levels in diabetic HPTCs compared with non-diabetic controls (Figure 7E).

The diabetic-induced metabolic programming observed in HPTCs from diabetic patients prompted us to explore the molecular mechanisms at the interface between glycolysis and OXPHOS during SARS-CoV-2 infections. To assess the role of OXPHOS in viral infection, we treated the cells with DCA, an inhibitor of mitochondrial pyruvate dehydrogenase kinase (PDK) (Figure S9F), which results in activation of mitochondrial OXPHOS at the expense of glycolysis (Bonnet et al., 2007; Cook et al., 2021; Eleftheriadis et al., 2013). Importantly, when diabetic and non-diabetic patient-derived HPTCs were treated with DCA, we observed a significant decrease in SARS-CoV-2 mRNA expression levels (Figures 7F and 7G). Therefore, our results show that both altered energy metabolism and increased ACE2 impact SARS-CoV-2 infections in kidney patient cells.

DISCUSSION

There is an urgent need to better understand the complex relationships between pre-existing conditions in COVID-19 patients, which may exacerbate viral infection and disease outcomes. Diabetes has emerged as one of the most frequent co-morbidities associated with severity and mortality of COVID-19. Here, we developed a unique culture system to generate diabetic human kidney organoids, based on oscillation of glucose levels also observed in DM patients. Our approach preserved renal cell types while recapitulating early hallmarks of diabetic kidney disease (Sas et al., 2016).

When analyzing kidney organoids for the expression of the SARS-CoV-2 entry receptor ACE2, we consistently observed ACE2 positivity in tubular-like cells by confocal microscopy, confirming our previous observations using single-cell profiling in human kidney organoids (Monteil et al., 2020) and earlier reports using single-cell profiling (Zou et al., 2020) and immunohistochemistry in the mouse (Soler et al., 2013; Ye et al., 2006) and human kidney (Lely et al., 2004). Recently, single-cell profiling from 436 patients suggested that increases in ACE2 expression within lungs and kidneys may increase the risk of SARS-CoV-2 infections (Jiang et al., 2020). Our results now show that a high

and the deposition of a basement membrane (bm). Scale bars, 2 μm, 200 nm (magnified views in 1 and 2), and 2 μm (magnified views in 3). Representative images of infected *BSG* KO specimens (right) show numerous viral particles in the intercellular space (4) and in the cell surface (5). Details for podocyte-like cells (6) exhibiting podocyte-related structures including primary (pp) and the deposition of a basement membrane (bm). Scale bars, 2 and 5 μm, 200 nm (magnified views in 4 and 5), and 1 μm (magnified view in 6).

(G) SARS-CoV-2 mRNA expression levels in mock-treated or SARS-CoV-2-infected WT and *BSG* KO kidney organoids at 3 dpi by qPCR. Data are mean ± SD. n = 1 independent experiment from a pool of 12 organoids/group with at least two technical replicates each.

(H) Immunofluorescence of mock or SARS-CoV-2-infected WT and *BSG* KO kidney organoids at 3 dpi for ACE2 (green), viral nuclear protein (NP, red), LTL (gray), and DAPI (blue). Scale bars, 250 μm.

See also Figure S8.

CellPress
OPEN ACCESS

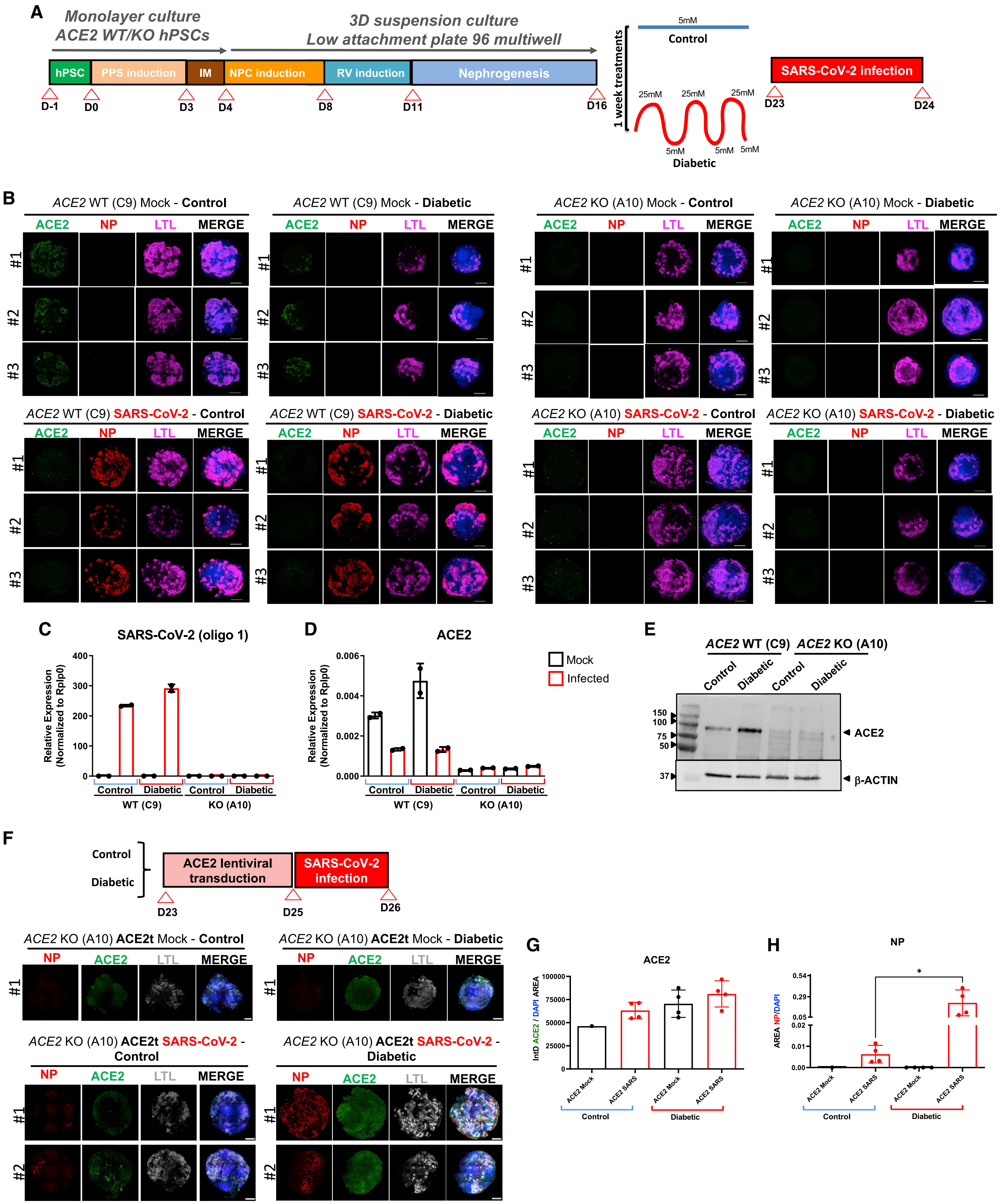

Figure 6. ACE2 expression in diabetic human kidney organoids sustains SARS-CoV-2 infections

(A) Experimental scheme for the infection with SARS-CoV-2 of WT and *ACE2* KO kidney organoids under control or diabetic conditions.

(B) Immunofluorescence of mock or SARS-CoV-2-infected specimens under control or diabetic conditions at 1 dpi for ACE2 (green), virus nuclear protein (NP, red), LTL (magenta), and DAPI (blue). Scale bars, 250 μm. n = 3 organoids per condition.

(legend continued on next page)

oscillatory glucose regime induces the expression of ACE2 at both the mRNA and protein levels, consistent with previous findings showing increased ACE2 levels in the kidney cortex from db/db mice and STZ diabetic mice (Ye et al., 2004) and proximal tubular cells in kidney biopsies from patients with diabetic kidney disease (Menon et al., 2020). Importantly, concurrent with enhanced ACE2 expression, we observed increased viral loads at the mRNA and protein levels in the diabetic kidney organoids. Following infection, we detected a marked decrease in ACE2 mRNA expression in accordance with previous results using colon- and ileum-derived human intestinal organoids (Triana et al., 2021). Single-cell profiling showed a decrease in OXPHOS and decrease in glycolytic-based metabolism as well as a hypoxic signature in response to SARS-CoV-2 infection in diabetic organoids.

Nearly two decades ago, we and others showed in *ace2* mutant mice that ACE2 is a negative regulator of the RAS system and genetically controls cardiovascular function and damage of multiple organs such as the lung, liver, and kidney (Clarke and Turner, 2012; Crackower et al., 2002). From the beginning of the pandemic, ACE2 took center stage in the COVID-19 outbreak as a receptor for the spike glycoprotein of SARS-CoV-2 (Wrapp et al., 2020; Zhou et al., 2020), which spurred the development of vaccines and therapies targeting the ACE2-SARS-CoV-2 spike interaction. Since various other candidate receptors have been reported (and considering the drug and vaccine development landscape), it is therefore paramount to establish whether ACE2 is not only sufficient for infection but is in fact essential. To evaluate the key role of ACE2 in SARS-CoV-2 infection, we therefore generated *ACE2* KO hPSCs derived kidney organoids, which showed preserved renal differentiation; though, in line with the previously reported functions of ACE2, these ACE2 mutant organoids displayed differences with regard to OXPHOS, lipid metabolism, as well as angiogenesis/endothelium (Peña-Silva et al., 2012). To further demonstrate the unique role of ACE2 for viral entry, we also generated *BSG/CD147* KO and *NRP1* KO hPSC lines as others have highlighted its role in SARS-CoV-2 infection (Cantuti-Castelvetri et al., 2020; Daly et al., 2020; Shilts et al., 2021; Wang et al., 2020). However, *BSG* KO and *NPR1* KO kidney organoids clearly supported viral infection as demonstrated by confocal microscopy and TEM analysis, thus excluding an essential role of BSG or NRP1 in renal SARS-CoV-2 infections.

For many of the systemic manifestations of COVID-19, it is unclear whether the pathology is a secondary "side effect" of the SARS-CoV-2 infection, such as immune activation or altered coagulation, or whether it is also due to a direct SARS-CoV-2 infection of specific organs. We first described that kidney organoids can shed progeny SARS-CoV-2 viruses (Monteil et al., 2020, 2021), and later investigations confirmed SARS-CoV-2 kidney tropism (Braun et al., 2020; Diao et al., 2021; Jansen et al., 2021; Puelles et al., 2020), including the ability to replicate in human kidney cells, thereby establishing an association of kidney infection by SARS-CoV-2 with shorter survival time and increased incidence of AKI in COVID-19 patients (Braun et al., 2020; Puelles et al., 2020). However, it should be noted that other studies failed to detect SARS-CoV-2 in kidneys (Golmai et al., 2020; el Jamal et al., 2021; Santoriello et al., 2020; Volbeda et al., 2021). Our results now indicate that imbalances in cellular metabolism and ACE2 expression in kidney organoids due to elevated glucose levels directly lead to higher viral loads upon infection, potentially leading to a switch from an OXPHOS to an aerobic glycolytic state that could further contribute to higher susceptibility to SARS-CoV-2 infection. Similar results were observed in kidney proximal tubular cells isolated from diabetic patients, which exhibited increased altered mitochondrial respiration and enhanced glycolysis that correlated with higher SARS-CoV-2 infections compared with non-diabetic samples. Interestingly, the exposure of kidney patient cells to a metabolic modulator that boosts mitochondrial OXPHOS at the expense of glycolysis resulted in decreased SARS-CoV-2 infection, suggesting that both ACE2 expression and altered metabolism affect the outcome of SARS-CoV-2 infection in human kidney cells, which is in line with findings in our ACE2 re-expression experiments.

Overall, our results provide insights into the identification of diabetic-induced metabolic programming as a critical event that increases the susceptibility of kidney cells to SARS-CoV-2 infection. We hope that our observation will open the door to the identification of new interventions in COVID-19 pathogenesis targeting energy metabolism.

Limitations of study

Future work will be needed to address limitations of our study. (1) Utilizing more complex culture systems, including the assembly of a vascular component to kidney organoids, would help to further explore the impact of a high oscillatory glucose regimen in a more physiologically relevant context while also allowing us to better mirror diabetic endothelial cell dysfunction in the renal context. (2) The design of our studies focused on the early

(C) SARS-CoV-2 mRNA expression levels of mock or SARS-CoV-2-infected WT and *ACE2* KO kidney organoids in control or diabetic conditions by qPCR. Data are mean ± SD. n = 1 independent experiment from a pool of 12 organoids/group with two technical replicates each.

(D) *ACE2* mRNA expression levels of mock or SARS-CoV-2-infected WT and *ACE2* KO kidney organoids in control or diabetic conditions by qPCR. Data are mean ± SD. n = 1 independent experiment from a pool of 12 organoids/group with two technical replicates each.

(E) Protein levels of ACE2 in WT and *ACE2* KO kidney organoids under control or diabetic conditions by western blot analysis. β-actin was used as loading control. Data from a pool of 12 organoids/group are shown.

(F) Lentiviral transduction of ACE2 in *ACE2* KO kidney organoids (ACE2t) under control or diabetic conditions. Immunofluorescence of mock or SARS-CoV-2-infected specimens at 1 dpi for the viral nuclear protein (NP, red), ACE2 (green), LTL (white), and DAPI (blue). Scale bars, 250 and 50 μm (magnified views). n = 1 organoid (mock) and n = 2 organoids (SARS-CoV-2 infected).

(G) Quantification of ACE2 expression (shown as integrated density-IntD) in (F). Data are mean ± SD. n = 2 organoid per condition performing two technical replicates. No statistically significant differences were observed. One-way ANOVA, Tukey's multiple comparisons test.

(H) Quantification of the area of NP^+ cells in (F). Data are mean ± SD. n = 2 organoid per condition performing two technical replicates. *$p < 0.05$, one-way ANOVA, Tukey's multiple comparisons test.

See also Data S1.

CellPress
OPEN ACCESS

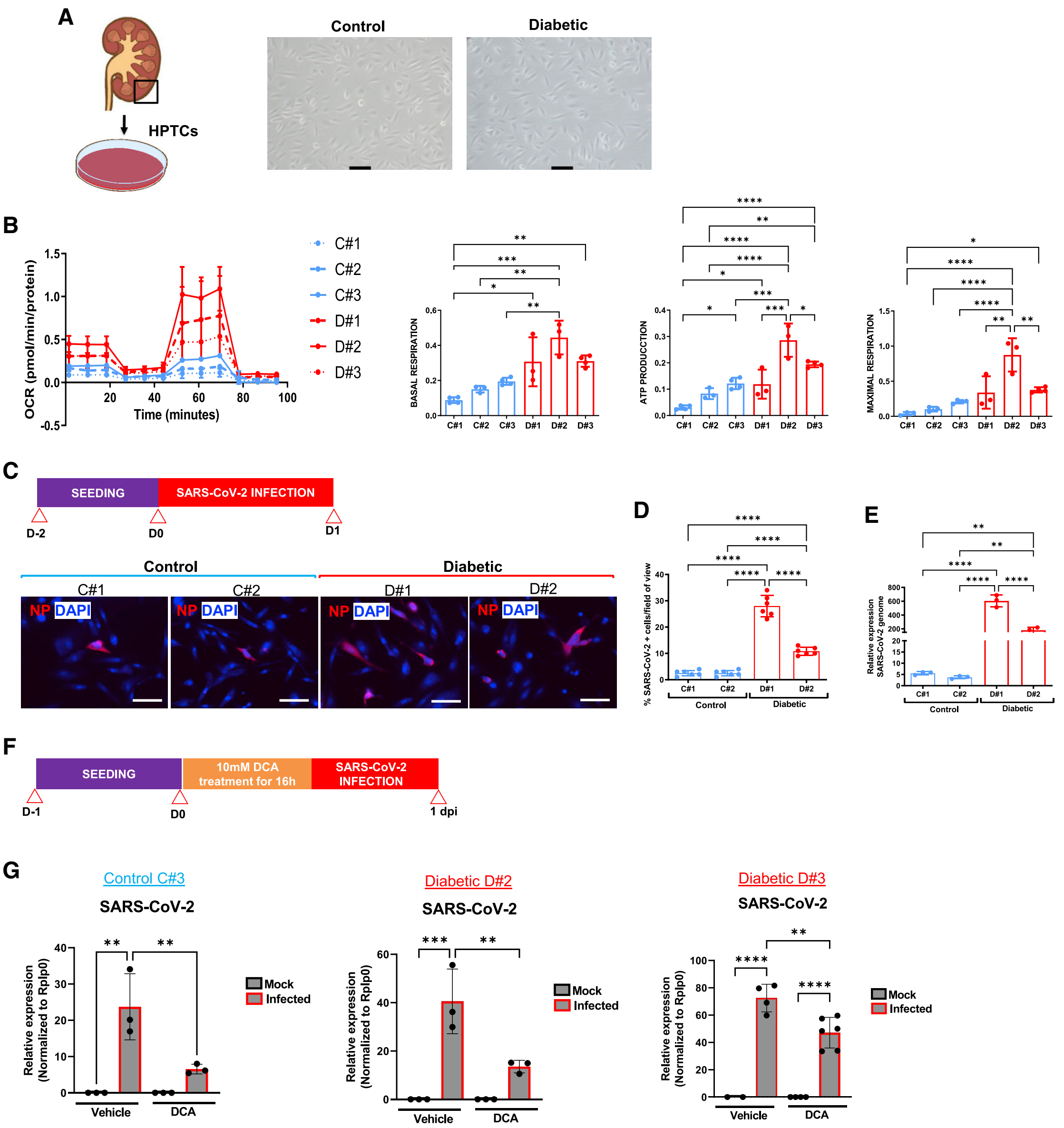

Figure 7. SARS-CoV-2 infection in tubular epithelial cells derived from diabetic human kidney biopsies

(A) Representative bright-field images of HPTCs from non-diabetic (control) or diabetic patient kidney biopsies. Scale bars, 100 μm.

(B) Seahorse analysis of control and diabetic HPTCs. The oxygen consumption rate (OCR) data are normalized to total protein. Basal respiration, cellular ATP production, and maximal respiration are shown. Data are mean ± SD from at least n = 3 biological replicates/group. *p < 0.05; **p < 0.005; ***p < 0.0005; ****p < 0.0001, one-way ANOVA, Tukey's multiple comparisons test.

(C) Experimental scheme for SARS-CoV-2 infection in control or diabetic HPTCs. Immunofluorescence of infected control or diabetic HPTCs at 1 dpi for the viral nuclear protein (NP, red) and DAPI (blue). Scale bars, 100 μm.

(D) Quantification of NP^+ cells in (C). Data are mean ± SD. n = 2 independent biological replicates per condition performing at least six technical replicates. ****p < 0.0001, one-way ANOVA, Tukey's multiple comparisons test.

(E) qPCR analysis of SARS-CoV-2-infected control or diabetic HPTCs at 1 dpi for the detection of SARS-CoV-2 mRNA. Data are mean ± SD. n = 2 independent biological replicates per condition with at least three technical replicates. **p < 0.01; ****p < 0.0001, one-way ANOVA, Tukey's multiple comparisons test.

(F) Experimental scheme for SARS-CoV-2 infection in control or diabetic HPTCs treated with DCA or vehicle for 16 h prior infection.

(G) qPCR analysis of mock or SARS-CoV-2-infected control or diabetic HPTCs exposed to DCA or vehicle at 1 dpi. Data are mean ± SD. n = 1 independent experiment with at least two technical replicates. **p < 0.005; ***p < 0.001; ****p < 0.0001, one-way ANOVA, Tukey's multiple comparisons test.

See also Figure S9.

stages of infection. As such, we cannot make any predictions with respect to the observed changes at later stages of the disease process. In this regard, inflammation represents a complex network of pathways that are influenced by external processes, which currently cannot be simulated in our model. (3) We did not study pancreatic organoids, and the pancreas is one of the major metabolic organs that could explain later complications of COVID-19.

STAR★METHODS

Detailed methods are provided in the online version of this paper and include the following:

- KEY RESOURCES TABLE
- RESOURCE AVAILABILITY
  - Lead contact
  - Materials availability
  - Data and code availability
- EXPERIMENTAL MODEL AND SUBJECT DETAILS
  - Cells
  - SARS-CoV-2 isolate
- METHOD DETAILS
  - Generation of genome edited hPSC lines
  - Kidney organoid differentiation
  - Glucose challenge in kidney organoids
  - Isolation of proximal tubular-like cells from kidney organoids
  - ACE2 mRNA half-life in kidney organoids exposed to 5mM and 5-25 mM glucose conditions
  - ACE2 lentiviral production and transduction of kidney organoids
  - SARS-CoV-2 infections of kidney organoids and proximal tubular kidney cells
  - SARS-CoV-2 infections of proximal tubular kidney cells exposed to DCA
  - Single-cell RNA sequencing
  - Protein extraction and western blot analysis in kidney organoids
  - Histology and Immunocytochemistry
  - Electron microscopy
  - qRT-PCR
  - Oxygen consumption rate (OCR)
- QUANTIFICATION AND STATISTICAL ANALYSIS
  - scRNA-Seq data analysis
  - Image analysis
  - Data representation and statistical analysis

SUPPLEMENTAL INFORMATION

Supplemental information can be found online at https://doi.org/10.1016/j.cmet.2022.04.009.

ACKNOWLEDGMENTS

We thank all members from our laboratories for helpful discussions and technical support. This work has received funding from the European Research Council (ERC) under the European Union's Horizon 2020 Research and Innovation Programme (StG-2014-640525_REGMAMKID to P.P. and N.M. and CoG-2020_101002478_ENGINORG to N.M.). This research has been supported by EIT Health under grant ID 20366 (R2U-Tox-Assay) to E.G. and N.M. N.M. is also supported by the MCIN/AEI/ 10.13039/501100011033 (PID2020-119929RB-I00) and MCIN/European Union—"Next Generation EU/PRTR" (PLEC2021-008127) and the Generalitat de Catalunya and CERCA Programme (2017 SGR 1306), and the IBEC Faster Future program (A por la COVID-19). This work was supported in part by the ISCIII and co-financed by FEDER (TERCEL RETIC RD16/0011/0005, RD16/0011/0027, PTC20/00013, and PTC20/00130 to N.M.), CIBERONC (CB16/12/00489 to F.P., A.V.-Z., and A.U.-A.), Gobierno de Navarra Departamento de Desarrollo Económico y Empresarial AGATA (0011-1411-2020-000011), and DIANA (0011-1411-2017-000029) to F.P., A.V.-Z., and A.U.-A. F.G. is supported by the Ramon y Cajal Grant—Biomedicine (RYC-2014-16751) from the Ministry of Economy and Competitivity (MINECO), Spain. A.M. is supported by the IBEC International PhD Programme "La Caixa" Severo Ochoa fellowships (LCF/BQ/SO16/52270019). This project has received funding from the European Union's Horizon 2020 research and innovation program under grant agreement no. 874827 BRAV3 to N.M. and C.T. M.L.S. and S.B. are supported by start-up funds from the College of Medicine at the University of Florida, Gainesville. J.M.P. and the research leading to these results have received funding from the T. von Zastrow Foundation, the FWF Wittgenstein award (Z 271-B19), the Austrian Academy of Sciences and the Canada 150 Research Chairs Program F18-01336, as well as the Canadian Institutes of Health Research COVID-19 grants F20-02343 and F20-02015. O.H.A. is supported by a Swiss National Science Foundation fellowship (P400PM_194473/1). A.M. is supported by Swedish Research Council (2018-05766). This project has received funding from the Innovative Medicines Initiative 2 Joint Undertaking (JU) under grant agreement no. 101005026. The JU receives support from the European Union's Horizon 2020 research and innovation program and EFPIA (A.M., J.M.P., and N.M.). This research has been supported by the project Ayudas SARS-CoV-2/COVID-19 (ACE2-ORG-COV20/00278) from Instituto de Salud Carlos III/FEDER to A.U.-A., F.P., and N.M. and from Instituto de Salud Carlos III and European Union—Next Generation EU, Plan de Recuperación Transformación y Resiliencia (TERAV/ISCIII RD21/0017/0018) to N.M. This project has received funding from Ayudas Fundación BBVA a Equipos de Investigación Científica SARS-CoV-2 y COVID-19 through the project "Identifying SARS-CoV-2-host cell interactions exploiting CRISPR-Cas9-engineered human organoids: through the development of specific therapies against COVID19" (A.M., J.M.P., and N.M.) and Fundació la Marató de TV3 (201910-31 and 202125-3) to N.M.

AUTHOR CONTRIBUTIONS

This study was conceived and designed by N.M. N.M. and J.M.P. wrote the paper. E.G. and P.P. performed all cell culture experiments in wild-type cell lines. E.G. and P.P. performed all cell culture experiments in genome-edited cell lines. E.G. and P.P. performed all histological analysis. E.G. performed TEM analysis. E.G., P.P., A.M., G.J., A.L., A.H., R.O., D.M.-R., C.T., P.D.-P., O.H.A., J.M.P., and N.M. performed organoid data analysis including qPCR, western blot, and image quantification. M.L.S., V.M., S.B., and A.M. performed all the experiments involving SARS-CoV-2, including isolation, and helped with manuscript editing. F.G. and A.M. performed CRISPR-Cas9 lines. M.L.S., S.B., and A.V.-Z. prepared scRNA-seq samples. A.U.-A., J.P.R., and F.P. performed all the single-cell data analysis on genome-edited derived organoids. A.G. contributed to Seahorse analysis in LTL^+ cells and HPTCs. C.H.P. performed one cell culture experiment to isolate LTL+ cells from kidney organoids for seahorse analysis and performed experiments to detect *COL3A1*, *COL4A1*, and *PGC1α* by qPCR in kidney organoids and western blot to detect ACE2 and NPR1. C.H.P. performed inmunofluorescence analysis to detect PODXL and LTL in kidney organoids in Figure S1A and PGC1α, LTL, and NaK (sodium potassium ATPase) in LTL cells in Figure S2D. D.M.-R., P.-V.A., and J.M.C. performed patient cell isolation and characterization.

DECLARATION OF INTERESTS

A patent has been submitted to use human organoids to study SARS-CoV-2 infections and possibly develop new therapies. J.M.P. is a shareholder of Apeiron Biologics, which is developing ACE2 decoys for COVID-19 therapy.

CellPress
OPEN ACCESS

Received: September 2, 2021
Revised: February 2, 2022
Accepted: April 19, 2022
Published: May 12, 2022

REFERENCES

Baer, P.C., and Geiger, H. (2008). Human renal cells from the thick ascending limb and early distal tubule: characterization of primary isolated and cultured cells by reverse transcription polymerase chain reaction. Nephrology (Carlton) *13*, 316–321. https://doi.org/10.1111/j.1440-1797.2008.00927.x.

Baer, P.C., Nockher, W.A., Haase, W., and Scherberich, J.E. (1997). Isolation of proximal and distal tubule cells from human kidney by immunomagnetic separation. Technical note. Kidney Int. *52*, 1321–1331. https://doi.org/10.1038/ki.1997.457.

Bastard, P., Michailidis, E., Hoffmann, H.H., Chbihi, M., Le Voyer, T., Rosain, J., Philippot, Q., Seeleuthner, Y., Gervais, A., Materna, M., et al. (2021). Auto-antibodies to type I IFNs can underlie adverse reactions to yellow fever live attenuated vaccine. J. Exp. Med. *218*, e20202486. https://doi.org/10.1084/jem.20202486.

Becker, M.M., Graham, R.L., Donaldson, E.F., Rockx, B., Sims, A.C., Sheahan, T., Pickles, R.J., Corti, D., Johnston, R.E., Baric, R.S., and Denison, M.R. (2008). Synthetic recombinant bat SARS-like coronavirus is infectious in cultured cells and in mice. Proc. Natl. Acad. Sci. USA *105*, 19944–19949. https://doi.org/10.1073/pnas.0808116105.

Bonnet, S., Archer, S.L., Allalunis-Turner, J., Haromy, A., Beaulieu, C., Thompson, R., Lee, C.T., Lopaschuk, G.D., Puttagunta, L., Bonnet, S., et al. (2007). A mitochondria-K+ channel axis is suppressed in cancer and its normalization promotes apoptosis and inhibits cancer growth. Cancer Cell *11*, 37–51. https://doi.org/10.1016/j.ccr.2006.10.020.

Braun, F., Lütgehetmann, M., Pfefferle, S., Wong, M.N., Carsten, A., Lindenmeyer, M.T., Nörz, D., Heinrich, F., Meißner, K., Wichmann, D., et al. (2020). SARS-CoV-2 renal tropism associates with acute kidney injury. Lancet *396*, 597–598. https://doi.org/10.1016/S0140-6736(20)31759-1.

Bülow, R.D., and Boor, P. (2019). Extracellular matrix in kidney fibrosis: more than just a scaffold. J. Histochem. Cytochem. *67*, 643–661. https://doi.org/10.1369/0022155419849388.

Cantuti-Castelvetri, L., Ojha, R., Pedro, L.D., Djannatian, M., Franz, J., Kuivanen, S., van der Meer, F., Kallio, K., Kaya, T., Anastasina, M., et al. (2020). Neuropilin-1 facilitates SARS-CoV-2 cell entry and infectivity. Science *370*, 856–860. https://doi.org/10.1126/science.abd2985.

Chen, Y., Yu, Q., and Xu, C.-B. (2017). A convenient method for quantifying collagen fibers in atherosclerotic lesions by ImageJ software. Int. J. Clin. Exp. Med. *10*, 14927–14935.

Clarke, N.E., and Turner, A.J. (2012). Angiotensin-converting enzyme 2: the first decade. Int. J. Hypertens. *2012*, 307315. https://doi.org/10.1155/2012/307315.

Clement, K., Rees, H., Canver, M.C., Gehrke, J.M., Farouni, R., Hsu, J.Y., Cole, M.A., Liu, D.R., Joung, J.K., Bauer, D.E., and Pinello, L. (2019). CRISPResso2 provides accurate and rapid genome editing sequence analysis. Nat. Biotechnol. *37*, 224–226. https://doi.org/10.1038/s41587-019-0032-3.

Codo, A.C., Davanzo, G.G., Monteiro, L.B., de Souza, G.F., Muraro, S.P., Virgilio-da-Silva, J.V., Prodonoff, J.S., Carregari, V.C., de Biagi Junior, C.A.O., Crunfli, F., et al. (2020). Elevated glucose levels favor SARS-CoV-2 infection and monocyte response through a HIF-1α/glycolysis-dependent axis. Cell Metab *32*, 437–446.e5. https://doi.org/10.1016/j.cmet.2020.07.007.

Cook, K.M., Shen, H., McKelvey, K.J., Gee, H.E., and Hau, E. (2021). Targeting glucose metabolism of cancer cells with dichloroacetate to radiosensitize high-grade gliomas. Int. J. Mol. Sci. *22*, 7265. https://doi.org/10.3390/ijms22147265.

Crackower, M.A., Sarao, R., Oudit, G.Y., Yagil, C., Kozieradzki, I., Scanga, S.E., Oliveira-Dos-Santos, A.J., da Costa, J., Zhang, L., Pei, Y., et al. (2002). Angiotensin-converting enzyme 2 is an essential regulator of heart function. Nature *417*, 822–828. https://doi.org/10.1038/nature00786.

Daly, J.L., Simonetti, B., Klein, K., Chen, K.E., Williamson, M.K., Antón-Plágaro, C., Shoemark, D.K., Simón-Gracia, L., Bauer, M., Hollandi, R., et al. (2020). Neuropilin-1 is a host factor for SARS-CoV-2 infection. Science *370*, 861–865. https://doi.org/10.1126/science.abd3072.

Deng, Y.Y., Zheng, Y., Cai, G.Y., Chen, X.M., and Hong, Q. (2020). Single-cell RNA sequencing data suggest a role for angiotensin-converting enzyme 2 in kidney impairment in patients infected with 2019-novel coronavirus. Chin. Med. J. (Engl) *133*, 1129–1131. https://doi.org/10.1097/CM9.0000000000000783.

Dhillon, P., Park, J., Hurtado Del Pozo, C., Li, L., Doke, T., Huang, S., Zhao, J., Kang, H.M., Shrestra, R., Balzer, M.S., et al. (2021). The nuclear receptor ESRRA protects from kidney disease by coupling metabolism and differentiation. Cell Metab *33*, 379–394.e8. https://doi.org/10.1016/j.cmet.2020.11.011.

Diao, B., Wang, C., Wang, R., Feng, Z., Zhang, J., Yang, H., Tan, Y., Wang, H., Wang, C., Liu, L., et al. (2021). Human kidney is a target for novel severe acute respiratory syndrome coronavirus 2 infection. Nat. Commun. *12*, 2506. https://doi.org/10.1038/s41467-021-22781-1.

El Jamal, S.M., Pujadas, E., Ramos, I., Bryce, C., Grimes, Z.M., Amanat, F., Tsankova, N.M., Mussa, Z., Olson, S., Salem, F., et al. (2021). Tissue-based SARS-CoV-2 detection in fatal COVID-19 infections: sustained direct viral-induced damage is not necessary to drive disease progression. Hum. Pathol. *114*, 110–119. https://doi.org/10.1016/j.humpath.2021.04.012.

Eleftheriadis, T., Pissas, G., Karioti, A., Antoniadi, G., Antoniadis, N., Liakopoulos, V., and Stefanidis, I. (2013). Dichloroacetate at therapeutic concentration alters glucose metabolism and induces regulatory T-cell differentiation in alloreactive human lymphocytes. J. Basic Clin. Physiol. Pharmacol. *24*, 271–276. https://doi.org/10.1515/jbcpp-2013-0001.

Ellinghaus, D., Degenhardt, F., Bujanda, L., Buti, M., Albillos, A., Invernizzi, P., Fernández, J., Prati, D., Baselli, G., Asselta, R., et al. (2020). The ABO blood group locus and a chromosome 3 gene cluster associate with SARS-CoV-2 respiratory failure in an Italian-Spanish genome-wide association analysis. Preprint at MedRxiv. https://doi.org/10.1101/2020.05.31.20114991.

Friederich-Persson, M., and Persson, P. (2020). Mitochondrial angiotensin II receptors regulate oxygen consumption in kidney mitochondria from healthy and type 1 diabetic rats. Am. J. Physiol. Renal Physiol. *318*, F683–F688. https://doi.org/10.1152/ajprenal.00417.2019.

Friederich, M., Fasching, A., Hansell, P., Nordquist, L., and Palm, F. (2008). Diabetes-induced up-regulation of uncoupling protein-2 results in increased mitochondrial uncoupling in kidney proximal tubular cells. Biochim. Biophys. Acta *1777*, 935–940. https://doi.org/10.1016/j.bbabio.2008.03.030.

Garreta, E., Prado, P., Tarantino, C., Oria, R., Fanlo, L., Martí, E., Zalvidea, D., Trepat, X., Roca-Cusachs, P., Gavaldà-Navarro, A., et al. (2019). Fine tuning the extracellular environment accelerates the derivation of kidney organoids from human pluripotent stem cells. Nat. Mater. *18*, 397–405. https://doi.org/10.1038/s41563-019-0287-6.

Genuth, S., Lipps, J., Lorenzi, G., Nathan, D.M., Davis, M.D., Lachin, J.M., and Cleary, P.A.; Writing Team for the Diabetes Control and Complications Trial/Epidemiology of Diabetes Interventions and Complications Research Group (2002). Effect of intensive therapy on the microvascular complications of type 1 diabetes mellitus. JAMA *287*, 2563–2569. https://doi.org/10.1001/jama.287.19.2563.

Golmai, P., Larsen, C.P., DeVita, M.V., Wahl, S.J., Weins, A., Rennke, H.G., Bijol, V., and Rosenstock, J.L. (2020). Histopathologic and ultrastructural findings in postmortem kidney biopsy material in 12 patients with AKI and COVID-19. J. Am. Soc. Nephrol. *31*, 1944–1947. https://doi.org/10.1681/ASN.2020050683.

Haga, S., Yamamoto, N., Nakai-Murakami, C., Osawa, Y., Tokunaga, K., Sata, T., Yamamoto, N., Sasazuki, T., and Ishizaka, Y. (2008). Modulation of TNF-alpha-converting enzyme by the spike protein of SARS-CoV and ACE2 induces TNF-alpha production and facilitates viral entry. Proc. Natl. Acad. Sci. USA *105*, 7809–7814. https://doi.org/10.1073/pnas.0711241105.

Härdtner, C., Mörke, C., Walther, R., Wolke, C., and Lendeckel, U. (2013). High glucose activates the alternative ACE2/Ang-(1–7)/Mas and APN/Ang IV/IRAP RAS axes in pancreatic β-cells. Int. J. Mol. Med. *32*, 795–804. https://doi.org/10.3892/ijmm.2013.1469.

Hoffmann, M., Kleine-Weber, H., Schroeder, S., Krüger, N., Herrler, T., Erichsen, S., Schiergens, T.S., Herrler, G., Wu, N.H., Nitsche, A., et al. (2020). SARS-CoV-2 cell entry depends on ACE2 and TMPRSS2 and is blocked by a clinically proven protease inhibitor. Cell *181*, 271–280.e8. https://doi.org/10.1016/j.cell.2020.02.052.

Imai, Y., Kuba, K., Rao, S., Huan, Y., Guo, F., Guan, B., Yang, P., Sarao, R., Wada, T., Leong-Poi, H., et al. (2005). Angiotensin-converting enzyme 2 protects from severe acute lung failure. Nature *436*, 112–116. https://doi.org/10.1038/nature03712.

Jansen, J., Reimer, K.C., Nagai, J.S., Varghese, F.S., Overheul, G.J., de Beer, M., Roverts, R., Daviran, D., Fermin Las, W.B., Beukenboom, M., et al. (2021). SARS-CoV-2 infects the human kidney and drives fibrosis in kidney organoids. Cell Stem Cell *29*, 217–231.e8. https://doi.org/10.1016/j.stem.2021.12.010.

Jiang, X., Eales, J.M., Scannali, D., Nazgiewicz, A., Prestes, P., Maier, M., Denniff, M., Xu, X., Saluja, S., Cano-Gamez, E., et al. (2020). Hypertension and renin-angiotensin system blockers are not associated with expression of angiotensin-converting enzyme 2 (ACE2) in the kidney. Eur. Heart J. *41*, 4580–4588. https://doi.org/10.1093/eurheartj/ehaa794.

Khalili, S., Sabaghian, T., Sedaghat, M., Soroureddin, Z., Askari, E., and Khalili, N. (2021). Prevalence, risk factors and outcomes associated with acute kidney injury in patients hospitalized for COVID-19: a comparative study between diabetic and nondiabetic patients. J. Diabetes Res. *2021*, 6666086. https://doi.org/10.1155/2021/6666086.

Korotkevich, G., Sukhov, V., Budin, N., Shpak, B., Artyomov, M.N., and Sergushichev, A. (2021). Fast gene set enrichment analysis. Preprint at bioRxiv. https://doi.org/10.1101/060012.

Kuba, K., Imai, Y., Rao, S., Gao, H., Guo, F., Guan, B., Huan, Y., Yang, P., Zhang, Y., Deng, W., et al. (2005). A crucial role of angiotensin converting enzyme 2 (ACE2) in SARS coronavirus-induced lung injury. Nat. Med. *11*, 875–879. https://doi.org/10.1038/nm1267.

Kuleshov, M.V., Jones, M.R., Rouillard, A.D., Fernandez, N.F., Duan, Q., Wang, Z., Koplev, S., Jenkins, S.L., Jagodnik, K.M., Lachmann, A., et al. (2016). Enrichr: a comprehensive gene set enrichment analysis web server 2016 update. Nucleic Acids Res. *44*, W90–W97. https://doi.org/10.1093/nar/gkw377.

Legrand, M., Bell, S., Forni, L., Joannidis, M., Koyner, J.L., Liu, K., and Cantaluppi, V. (2021). Pathophysiology of COVID-19-associated acute kidney injury. Nat. Rev. Nephrol. *17*, 751–764. https://doi.org/10.1038/s41581-021-00452-0.

Lely, A.T., Hamming, I., van Goor, H., and Navis, G.J. (2004). Renal ACE2 expression in human kidney disease. J. Pathol. *204*, 587–593. https://doi.org/10.1002/path.1670.

Li, S.Y., and Susztak, K. (2018). The role of peroxisome proliferator-activated receptor γ coactivator 1α (PGC-1α) in kidney disease. Semin. Nephrol. *38*, 121–126. https://doi.org/10.1016/j.semnephrol.2018.01.003.

Lim, S., Bae, J.H., Kwon, H.S., and Nauck, M.A. (2021). COVID-19 and diabetes mellitus: from pathophysiology to clinical management. Nat. Rev. Endocrinol. *17*, 11–30. https://doi.org/10.1038/s41574-020-00435-4.

Lin, W., Fan, J., Hu, L.F., Zhang, Y., Ooi, J.D., Meng, T., Jin, P., Ding, X., Peng, L.K., Song, L., et al. (2021). Single-cell analysis of angiotensin-converting enzyme II expression in human kidneys and bladders reveals a potential route of 2019 novel coronavirus infection. Chin. Med. J. (Engl) *134*, 935–943. https://doi.org/10.1097/CM9.0000000000001439.

Lindström, N.O., De Sena Brandine, G., Tran, T., Ransick, A., Suh, G., Guo, J., Kim, A.D., Parvez, R.K., Ruffins, S.W., Rutledge, E.A., et al. (2018a). Progressive recruitment of mesenchymal progenitors reveals a time-dependent process of cell fate acquisition in mouse and human nephrogenesis. Dev. Cell *45*, 651–660.e4. https://doi.org/10.1016/j.devcel.2018.05.010.

Lindström, N.O., McMahon, J.A., Guo, J., Tran, T., Guo, Q., Rutledge, E., Parvez, R.K., Saribekyan, G., Schuler, R.E., Liao, C., et al. (2018b). Conserved and divergent features of human and mouse kidney organogenesis. J. Am. Soc. Nephrol. *29*, 785–805. https://doi.org/10.1681/ASN.2017080887.

Maksimowski, N., Williams, V.R., and Scholey, J.W. (2020). Kidney ACE2 expression: implications for chronic kidney disease. PLoS One *15*, e0241534. https://doi.org/10.1371/journal.pone.0241534.

Marco, A., Selfa, I.L., Tarantino, C., González, F., and Montserrat, N. (2019). Engineering human pluripotent stem cells (hPSCs) lines with CRISPR/Cas9 for inducible knock out in kidney organoids. In The International Conference on Stem Cell Engineering (AIChE).

Marco, A., Selfa, I.L., Tarantino, C., González, F., and Montserrat, N. (2020). Engineering human pluripotent stem cells (hPSCs) lines with CRISPR/Cas9 for inducible knock out in kidney organoids. In EMBL·IBEC Winter Conference (IBEC).

Menon, R., Otto, E.A., Sealfon, R., Nair, V., Wong, A.K., Theesfeld, C.L., Chen, X., Wang, Y., Boppana, A.S., Luo, J., et al. (2020). SARS-CoV-2 receptor networks in diabetic and COVID-19-associated kidney disease. Kidney Int. *98*, 1502–1518. https://doi.org/10.1016/j.kint.2020.09.015.

Mizuiri, S., Hemmi, H., Arita, M., Ohashi, Y., Tanaka, Y., Miyagi, M., Sakai, K., Ishikawa, Y., Shibuya, K., Hase, H., and Aikawa, A. (2008). Expression of ACE and ACE2 in individuals with diabetic kidney disease and healthy controls. Am. J. Kidney Dis. *51*, 613–623. https://doi.org/10.1053/j.ajkd.2007.11.022.

Monteil, V., Kwon, H., Prado, P., Hagelkrüys, A., Wimmer, R.A., Stahl, M., Leopoldi, A., Garreta, E., Hurtado Del Pozo, C., Prosper, F., et al. (2020). Inhibition of SARS-CoV-2 infections in engineered human tissues using clinical-grade soluble human ACE2. Cell *181*, 905–913.e7. https://doi.org/10.1016/j.cell.2020.04.004.

Monteil, V., Dyczynski, M., Lauschke, V.M., Kwon, H., Wirnsberger, G., Youhanna, S., Zhang, H., Slutsky, A.S., Hurtado Del Pozo, C., Horn, M., et al. (2021). Human soluble ACE2 improves the effect of remdesivir in SARS-CoV-2 infection. EMBO Mol. Med. *13*, e13426. https://doi.org/10.15252/emmm.202013426.

Nadim, M.K., Forni, L.G., Mehta, R.L., Connor, M.J., Jr., Liu, K.D., Ostermann, M., Rimmelé, T., Zarbock, A., Bell, S., Bihorac, A., et al. (2020). COVID-19-associated acute kidney injury: consensus report of the 25th Acute Disease Quality Initiative (ADQI) Workgroup. Nat. Rev. Nephrol. *16*, 747–764. https://doi.org/10.1038/s41581-020-00356-5.

Peña Silva, R.A., Chu, Y., Miller, J.D., Mitchell, I.J., Penninger, J.M., Faraci, F.M., and Heistad, D.D. (2012). Impact of ACE2 deficiency and oxidative stress on cerebrovascular function with aging. Stroke *43*, 3358–3363. https://doi.org/10.1161/STROKEAHA.112.667063.

Puelles, V.G., Lütgehetmann, M., Lindenmeyer, M.T., Sperhake, J.P., Wong, M.N., Allweiss, L., Chilla, S., Heinemann, A., Wanner, N., Liu, S., et al. (2020). Multiorgan and renal tropism of SARS-CoV-2. N. Engl. J. Med. *383*, 590–592. https://doi.org/10.1056/NEJMc2011400.

Reich, H.N., Oudit, G.Y., Penninger, J.M., Scholey, J.W., and Herzenberg, A.M. (2008). Decreased glomerular and tubular expression of ACE2 in patients with type 2 diabetes and kidney disease. Kidney Int. *74*, 1610–1616. https://doi.org/10.1038/ki.2008.497.

Sandooja, R., Vura, N.V.R.K., and Morocco, M. (2020). Heightened ACE activity and unfavorable consequences in COVID-19 diabetic subjects. Int. J. Endocrinol. *2020*, 7847526. https://doi.org/10.1155/2020/7847526.

Santoriello, D., Khairallah, P., Bomback, A.S., Xu, K., Kudose, S., Batal, I., Barasch, J., Radhakrishnan, J., D'Agati, V., and Markowitz, G. (2020). Postmortem kidney pathology findings in patients with COVID-19. J. Am. Soc. Nephrol. *31*, 2158–2167. https://doi.org/10.1681/ASN.2020050744.

Sas, K.M., Kayampilly, P., Byun, J., Nair, V., Hinder, L.M., Hur, J., Zhang, H., Lin, C., Qi, N.R., Michailidis, G., et al. (2016). Tissue-specific metabolic reprogramming drives nutrient flux in diabetic complications. JCI Insight *1*, e86976. https://doi.org/10.1172/jci.insight.86976.

Shilts, J., Crozier, T.W.M., Greenwood, E.J.D., Lehner, P.J., and Wright, G.J. (2021). No evidence for basigin/CD147 as a direct SARS-CoV-2 spike binding receptor. Sci. Rep. *11*, 413. https://doi.org/10.1038/s41598-020-80464-1.

Soler, M.J., Wysocki, J., Ye, M., Lloveras, J., Kanwar, Y., and Batlle, D. (2007). ACE2 inhibition worsens glomerular injury in association with increased ACE expression in streptozotocin-induced diabetic mice. Kidney Int. *72*, 614–623. https://doi.org/10.1038/sj.ki.5002373.

Soler, M.J., Wysocki, J., and Batlle, D. (2013). ACE2 alterations in kidney disease. Nephrol. Dial. Transplant. *28*, 2687–2697. https://doi.org/10.1093/ndt/gft320.

Stuart, T., Butler, A., Hoffman, P., Hafemeister, C., Papalexi, E., Mauck, W.M., 3rd, Hao, Y., Stoeckius, M., Smibert, P., and Satija, R. (2019). Comprehensive integration of single-cell data. Cell *177*, 1888–1902.e21. https://doi.org/10.1016/j.cell.2019.05.031.

Triana, S., Metz-Zumaran, C., Ramirez, C., Kee, C., Doldan, P., Shahraz, M., Schraivogel, D., Gschwind, A.R., Sharma, A.K., Steinmetz, L.M., et al. (2021). Single-cell analyses reveal SARS-CoV-2 interference with intrinsic immune response in the human gut. Mol. Syst. Biol. *17*, e10232. https://doi.org/10.15252/msb.202110232.

Ugwueze, C.V., Ezeokpo, B.C., Nnolim, B.I., Agim, E.A., Anikpo, N.C., and Onyekachi, K.E. (2020). COVID-19 and diabetes mellitus: the link and clinical implications. Dubai Diabetes Endocrinol. J. *26*, 69–77. https://doi.org/10.1159/000511354.

van der Made, C.I., Simons, A., Schuurs-Hoeijmakers, J., van den Heuvel, G., Mantere, T., Kersten, S., van Deuren, R.C., Steehouwer, M., van Reijmersdal, S.V., Jaeger, M., et al. (2020). Presence of genetic variants among young men with severe COVID-19. JAMA *324*, 663–673. https://doi.org/10.1001/jama.2020.13719.

Vasquez-Muñoz, M., Arce-Alvarez, A., von Igel, M., Veliz, C., Ruiz-Esquide, G., Ramirez-Campillo, R., Alvarez, C., Ramirez-Velez, R., Crespo, F.A., Izquierdo, M., et al. (2021). Oscillatory pattern of glycemic control in patients with diabetes mellitus. Sci Rep. *11*, 5789. https://doi.org/10.1038/s41598-021-84822-5.

Volbeda, M., Jou-Valencia, D., van den Heuvel, M.C., Knoester, M., Zwiers, P.J., Pillay, J., Berger, S.P., van der Voort, P.H.J., Zijlstra, J.G., van Meurs, M., and Moser, J. (2021). Comparison of renal histopathology and gene expression profiles between severe COVID-19 and bacterial sepsis in critically ill patients. Crit. Care *25*, 202. https://doi.org/10.1186/s13054-021-03631-4.

Walls, A.C., Park, Y.J., Tortorici, M.A., Wall, A., McGuire, A.T., and Veesler, D. (2020). Structure, function, and antigenicity of the SARS-CoV-2 Spike glycoprotein. Cell *181*, 281–292.e6. https://doi.org/10.1016/j.cell.2020.02.058.

Wan, Y., Shang, J., Graham, R., Baric, R.S., and Li, F. (2020). Receptor recognition by the novel coronavirus from Wuhan: an analysis based on decade-long structural studies of SARS coronavirus. J. Virol. *94*. e00127-20. https://doi.org/10.1128/JVI.00127-20.

Wang, K., Chen, W., Zhang, Z., Deng, Y., Lian, J.Q., Du, P., Wei, D., Zhang, Y., Sun, X.X., Gong, L., et al. (2020). CD147-spike protein is a novel route for SARS-CoV-2 infection to host cells. Signal Transduct. Target. Ther. *5*, 283. https://doi.org/10.1038/s41392-020-00426-x.

Wrapp, D., Wang, N., Corbett, K.S., Goldsmith, J.A., Hsieh, C.L., Abiona, O., Graham, B.S., and McLellan, J.S. (2020). Cryo-EM structure of the 2019-nCoV spike in the prefusion conformation. Science *367*, 1260–1263. https://doi.org/10.1126/science.abb2507.

Writing Team for the Diabetes Control and Complications Trial/Epidemiology of Diabetes Interventions and Complications Research Group (2003). Sustained effect of intensive treatment of type 1 diabetes mellitus on development and progression of diabetic nephropathy: the Epidemiology of Diabetes Interventions and Complications (EDIC) study. JAMA *290*, 2159–2167. https://doi.org/10.1001/jama.290.16.2159.

Wysocki, J., Ortiz-Melo, D.I., Mattocks, N.K., Xu, K., Prescott, J., Evora, K., Ye, M., Sparks, M.A., Haque, S.K., Batlle, D., and Gurley, S.B. (2014). ACE2 deficiency increases NADPH-mediated oxidative stress in the kidney. Physiol. Rep. *2*, e00264. https://doi.org/10.1002/phy2.264.

Yang, Y.F., Li, T.C., Li, C.I., Liu, C.S., Lin, W.Y., Yang, S.Y., Chiang, J.H., Huang, C.C., Sung, F.C., and Lin, C.C. (2015). Visit-to-visit glucose variability predicts the development of end-stage renal disease in type 2 diabetes: 10-year follow-up of Taiwan diabetes study. Med. (Baltim.) *94*, e1804. https://doi.org/10.1097/MD.0000000000001804.

Ye, M., Wysocki, J., Naaz, P., Salabat, M.R., LaPointe, M.S., and Batlle, D. (2004). Increased ACE 2 and decreased ACE protein in renal tubules from diabetic mice: a renoprotective combination? Hypertension *43*, 1120–1125. https://doi.org/10.1161/01.HYP.0000126192.27644.76.

Ye, M., Wysocki, J., William, J., Soler, M.J., Cokic, I., and Batlle, D. (2006). Glomerular localization and expression of angiotensin-converting enzyme 2 and angiotensin-converting enzyme: implications for albuminuria in diabetes. J. Am. Soc. Nephrol. *17*, 3067–3075. https://doi.org/10.1681/ASN.2006050423.

Zhang, J.Y., Wang, X.M., Xing, X., Xu, Z., Zhang, C., Song, J.W., Fan, X., Xia, P., Fu, J.L., Wang, S.Y., et al. (2020a). Single-cell landscape of immunological responses in patients with COVID-19. Nat. Immunol. *21*, 1107–1118. https://doi.org/10.1038/s41590-020-0762-x.

Zhang, Q., Bastard, P., Liu, Z., Le Pen, J., Moncada-Velez, M., Chen, J., Ogishi, M., Sabli, I.K.D., Hodeib, S., Korol, C., et al. (2020b). Inborn errors of type I IFN immunity in patients with life-threatening COVID-19. Science *370*, eabd4570. https://doi.org/10.1126/science.abd4570.

Zhou, P., Yang, X.L., Wang, X.G., Hu, B., Zhang, L., Zhang, W., Si, H.R., Zhu, Y., Li, B., Huang, C.L., et al. (2020). A pneumonia outbreak associated with a new coronavirus of probable bat origin. Nature *579*, 270–273. https://doi.org/10.1038/s41586-020-2012-7.

Zou, X., Chen, K., Zou, J., Han, P., Hao, J., and Han, Z. (2020). Single-cell RNA-seq data analysis on the receptor ACE2 expression reveals the potential risk of different human organs vulnerable to 2019-nCoV infection. Front. Med. *14*, 185–192. https://doi.org/10.1007/s11684-020-0754-0.

STAR★METHODS

KEY RESOURCES TABLE

REAGENT or RESOURCE	SOURCE	IDENTIFIER
**Antibodies**		
Human ACE-2	Bio-Techne R&D Systems S.L	Cat# AF933-SP; RRID: AB_355722
Recombinant Anti-Neuropilin 1 antibody	Abcam	Cat# ab81321; RRID: AB_1640739
α-Tubulin	Abcam	Cat# ab4074; RRID: AB_2288001
β-Actin	Sigma Aldrich	Cat# A5316; RRID: AB_476743
IRDye 800CW donkey anti-Goat IgG Secondary Antibody	Licor	Cat# 925-32214; RRID: AB_2687553
IRDye 800CW Goat anti-Rabbit IgG Secondary Antibody	Licor	Cat# 925-32211; RRID: AB_2651127
Human ACE2	Bio-Techne R&D Systems	Cat# AF933; RRID: AB_355722
Laminin	Merck	Cat# L9393; RRID: AB_477163
Collagen I	Abcam	Cat# ab34710; RRID: AB_731684
Collagen IV	Merck	Cat# AB769; RRID: AB_92262
Fibronectin	Abcam	Cat# ab2413; RRID: AB_2262874
Lotus Tetragonolobus Lectin (LTL), Biotinylated	Vector laboratories	Cat# B-1325; RRID: AB_2336558
Podocin	Merck	Cat# P0372; RRID: AB_261982
E-Cadherin	BD Bioscience	Cat# 610181; RRID: AB_397580
Human Podocalyxin Biotinylated	R&D Systems	Cat# BAF1658; RRID: AB_356080
SARS-CoV/SARS-CoV-2 Nucleocapsid	Abyntek Biopharma	Cat# 40143-MM05; RRID: AB_2827977
CD147	Abcam	Cat# ab666; RRID: AB_305632
Human Nephrin	R&D Systems	Cat# AF4269 RRID: AB_2154851
Recombinant PE Anti-Sodium Potassium ATPase	Abcam	Cat# ab209299
PGC1α	R&D Systems	Cat# NBP1-04676; RRID: AB_1522118
CD31	Abcam	Cat#ab28364; RRID: AB_726362
MEIS1/2/3	Thermo Fisher	Cat#39795; RRID: AB_2750570
Anti-Goat Alexa Fluor 488-conjugated	Jackson ImmunoResearch	Cat# 705-545-147; RRID: AB_2336933
Anti-Goat IgG Alexa Fluor 555-conjugated	Fisher Scientific	Cat# A-21432; RRID: AB_2535853
Anti-rabbit IgG Alexa fluor 488-conjugated	Fisher Scientific	Cat# A21206; RRID: AB_2535792
Anti-rabbit IgG Alexa fluor 555-conjugated	Fisher Scientific	Cat# A-31572; RRID: AB_162543
Anti-Mouse IgG CyTM3-conjugated	Jackson ImmunoResearch	Cat# 715-165-151; RRID: AB_2315777
Anti-Sheep IgG Alexa Fluor 555-conjugated	Fisher Scientific	Cat# A-21436; RRID: AB_2535857
Dylight 649 Streptavidin	Vector Labs	Cat# SA-5649; RRID: AB_2336421
Fluorescein-conjugated LTL	Vector Labs	Cat# FL-1321; RRID: AB_2336559
Anti- human ACE2-APC	LS Bio	Cat# LS-C275129
Normal Rabbit IgG APC-conjugated Control	R&D systems	Cat#. IC105A; RRID: AB_10174791
Recombinant Anti-Wilms Tumor Protein antibody [CAN-R9(IHC)-56-2]	N/A	Cat# ab89901; RRID: AB_2043201
**Biological samples**		
Human kidney tubular cells	N/A	N/A
**Chemicals, peptides, and recombinant proteins**		
Essential 8 medium	ThermoFisher	Cat# A1517001
Vitronectin	ThermoFisher	Cat# A14700
Trypan blue solution	Sigma	Cat# T8154
EDTA solution	ThermoFisher	Cat# 15575-038
Accumax	Stem Cell Technologies	Cat# 07921

(*Continued on next page*)

Continued

REAGENT or RESOURCE	SOURCE	IDENTIFIER
RPMI 1640	ThermoFisher	Cat#21875-034
Advanced RPMI 1640	ThermoFisher	Cat#12633020
GlutaMAX (200 mM)	ThermoFisher	Cat# 35050–038
Penicillin/Streptomicin	ThermoFisher	Cat# 15140122
Dulbecco's Modified Eagle Medium (DMEM) glucose-free	ThermoFisher	Cat# 11966025
D-Glucose	Merck	Cat# G7021
D-Manitol	Merck	Cat# M9647
ITS	Merck	Cat#I3146
Fetal Bovine Serum (FBS)	Gibco	Cat# 10270-106
Human EGF	R&D systems	Cat# 236-EG-01M
CHIR99021	Merck	Cat#SML1046; CAS: 2 52917-06-9
Recombinant human FGF9	PeproTech	Cat# 100-23
Heparin	Merck	Cat# H3149; CAS: 9041-08-1
Activin A	R&D systems	Cat# 338-AC-050
Cell culture grade distilled water	ThermoFisher	Cat# 15230-089
Protease inhibitor cocktail	Roche	Cat#11836153001
Phosphate buffered saline (PBS) pH 7.4 (1x)	ThermoFisher	Cat#1001–015
RIPA buffer	Cell signaling	Cat#9806
4–15% Mini-PROTEAN TGX Precast Protein Gels	BioRad	Cat# 4561083
SYBR Green PCR Master Mix	Applied Biosystem	Cat# KK4605
Fluoromount-G	Southern Biotech	Cat# 0100-01
Triton X-100	Sigma	Cat# T8787
4′,6-Diamidino-2-Phenylindole, Dihydrochloride (DAPI)	ThermoFisher	Cat# D1306
DNA dye Draq5	Abcam	Cat# ab108410
Dimethyl Sulfoxide (DMSO)	Merck	Cat# D2650; CAS: 67-68-5
Paraformaldehyde	Aname	Cat# sc-281692
Donkey serum	Millipore	Cat# S30
Glutaraldehyde	Sigma-Aldrich	Cat# G7776
Sodium phosphate dibasic dihydrate ≥99.0%	Sigma-Aldrich	Cat# 71643
Sodium phosphate monobasic ≥98%	Sigma-Aldrich	Cat# S3139
Trizol	ThermoFisher	Cat# 15596018
Chloroform	Sigma Merck	Cat# 1.02445 CAS:67-66-3
2-Propanol	Panreac	Cat# 131090 CAS: 67-63-0
Ethanol	VWR	Cat# 100983.2500; CAS: 64-17-5
Actinomycin D	Sigma	Cat# A9415-5MG
Nuclease-Free Water	Ambion	Cat# AM9937
Lipofectamine RNAiMAX Transfection Reagent	ThermoFisher	Cat# 13778150
Dichloroacetic acid	Merck	Cat# 347795-10G
Renal Epithelial Cell Growth Medium	Lonza	Cat# CC-3190
Intercept (TBS) Blocking Buffer	LICOR	Cat# 927-60001
**Critical commercial assays**		
BCA Protein Assay Kit	ThermoFisher	Cat# 23225
cDNA Reverse Transcription Kit	Applied Biosystems	Cat# 4368813
Seahorse XFe96 FluxPak mini	Agilent Technologies	Cat# 102601-100
Chromium Single Cell 3' Library & Gel Bead Kit V3	10X Genomics (USA)	Cat# PN-1000075
NSQ 500/550 Hi Output KT v2.5 (75 CYS)	Illumina (San Diego, CA 92122 USA)	Cat# 20024906
Streptavidin/Biotin blocking kit	Vector laboratories	Cat# SP-2002

(*Continued on next page*)

Continued		
REAGENT or RESOURCE	SOURCE	IDENTIFIER
Deposited data		
scRNA seq data kidney organoids	This study	GEO: GSE181002
Data S1 Source Data	This study	https://doi.org/10.17632/3m6gs9hfsd.1
CRISPResso2parser.py	open source	https://doi.org/10.5281/zenodo.6457752; https://github.com/amarcog/CRISPResso2parser.py
Experimental models: Cell lines		
ES[4] Human Embryonic Stem Cell line	The National Bank of Stem Cells (ISCIII,Madrid)	https://www.isciii.es/
CBiPS1sv-4F-40	The National Bank of Stem Cells (ISCIII,Madrid)	https://www.isciii.es/
Vero E6	ATCC CRL 1586	N/A
Oligonucleotides		
crRNA sequences	This paper	N/A
ACE2ko Cr1 (5'-3') TAGACTACAATGAGAGGCTC	N/A	N/A
ACE2ko Cr2 (5'-3') GCCATTATATGAAGAGTATG	N/A	N/A
BSGko Cr1 (5'-3') CTTGGAGCCAAGGTCTTCTA	N/A	N/A
BSGko Cr2 (5'-3') TTCACTACCGTAGAAGACCT	N/A	N/A
NRP1ko_Cr1 (5'-3') GGAATTTGAAAGCTTTGACC	N/A	N/A
NRP1ko_Cr2 (5'-3') GGGGACTTTATCACTCCACT	N/A	N/A
MiSeq oligos	This paper	N/A
MiSeq_ACE2ko_F (5'-3') ACACTCTTTCCCTACACGA CGCTCTTCCGATCTTGTGTGCTTTGGGATAACAGGT	N/A	N/A
MiSeq_ACE2ko_R (5'-3') GACTGGAGTTCAGACGTG TGCTCTTCCGATCTGCCACACAGAGAGCTTCAGG	N/A	N/A
MiSeq_BSGko_F (5'-3') ACACTCTTTCCCTACACGA CGCTCTTCCGATCTGGGGAGGAGCCGCAGGTTC	N/A	N/A
MiSeq_BSGko_R (5'-3') GACTGGAGTTCAGACGTG TGCTCTTCCGATCTCGTCCTCCTTCAGCACCACG	N/A	N/A
MiSeq_NRP1ko_F (5'-3') TGGGGAAGTTGTT TAAGTGGGA	N/A	N/A
MiSeq_NRP1ko_R (5'-3') ATCCATCCCAGAT TTCTAGCCG	N/A	N/A
qPCR oligos	This paper	N/A
SARS-CoV-2 (oligo1)_Forward GCCTCTTCTC GTTCCTCATCAC	Eurofins	N/A
SARS-CoV-2 (oligo1)_Reverse AGCAGCATCA CCGCCATTG	Eurofins	N/A
SARS-CoV-2 (oligo2)_Forward AGCCTCTTCT CGTTCCTCATCAC	Eurofins	N/A
SARS-CoV-2 (oligo2)_Reverse CCGCCATTG CCAGCCATTC	Eurofins	N/A
TMPRSS2_Forward GTCCCCACTGTCTACGAGGT	Thermo Fisher	N/A
TMPRSS2_Reverse CAGACGACGGGGTTGGAAG	Thermo Fisher	N/A
NRP1_Forward GGCGCTTTTCGCAACGATAAA	Thermo Fisher	N/A
NRP1_Reverse TCGCATTTTTCACTTGGGTGAT	Thermo Fisher	N/A
BSG_Forward CCGCAACCACCTTACTCG	Thermo Fisher	N/A
BSG_Reverse GGACAGAGGTTTGGATGGTG	Thermo Fisher	N/A
ACE2_Forward CGAAGCCGAAGACCTGTTCTA	Thermo Fisher	N/A
ACE2_Reverse GGGCAAGTGTGGACTGTTCC	Thermo Fisher	N/A
HK2_Forward AGCCCTTTCTCCATCTCCTT	Thermo Fisher	N/A
HK2_Reverse AACCATGACCAAGTGCAGAA	Thermo Fisher	N/A

(*Continued on next page*)

CellPress
OPEN ACCESS

Continued

REAGENT or RESOURCE	SOURCE	IDENTIFIER
LDHA_Forward GGAGATCCATCATCTCTCCC	Thermo Fisher	N/A
LDHA_Reverse GGCCTGTGCCATCAGTATCT	Thermo Fisher	N/A
PGC1α _Forward CTGCTAGCAAGTTTGCCTCA	Thermo Fisher	N/A
PGC1α _Reverse AGTGGTGCAGTGACCAATCA	Thermo Fisher	N/A
COL3A1_Forward AGGACTGACCAAGATGGGAA	Thermo Fisher	N/A
COL3A1_Reverse AGGGGAGCTGGCTACTTCTC	Thermo Fisher	N/A
COL4A1_Forward CCTTTTGTCCCTTCACTCCA	Thermo Fisher	N/A
COL4A1_Reverse CTCCACGAGGAGCACAGC	Thermo Fisher	N/A
RPLP0 _Forward CCATTCTATCATCAACGGGTACAA	Thermo Fisher	N/A
RPLP0 _Reverse AGCAAGTGGGAAGGTGTAATCC	Thermo Fisher	N/A
SLC16A1_Forward GGCTGTCATGTATGGTGGAG	Thermo Fisher	N/A
SLC16A1_Reverse GACAAGCAGCCACCAACAATC	Thermo Fisher	N/A
PODXL_Forward GATAAGTGCGGCATACGGCT	Thermo Fisher	N/A
PODXL_Reverse GCTCGTACACATCCTTGGCA	Thermo Fisher	N/A
WT1_Forward GCCAGGATGTTTCCTAACGC	Thermo Fisher	N/A
WT1_Reverse CGAAGGTGACCGTGCTGTAA	Thermo Fisher	N/A
NPHS1_Forward GGCTCCCAGCAGAAACTCTT	Thermo Fisher	N/A
NPHS1_Reverse CACAGACCAGCAACTGCCTA	Thermo Fisher	N/A
SLC3A1_Forward CACCAATGCAGTGGGACAAT	Thermo Fisher	N/A
SLC3A1_Reverse CTGGGCTGAGTCTTTTGGAC	Thermo Fisher	N/A
MAFB_Forward GACGCAGCTCATTCAGCAG	Thermo Fisher	N/A
MAFB_Reverse CTCGCACTTGACCTTGTAGGC	Thermo Fisher	N/A
Endoglin_Forward CCTACGTGTCCTGGCTCATC	Thermo Fisher	N/A
Endoglin_Reverse GGTGTGTCTGGGAGCTTGAA	Thermo Fisher	N/A
Meis1_Forward GGGCATGGATGGAGTAGGC	Thermo Fisher	N/A
Meis1_Reverse GGGTACTGATGCGAGTGCAG	Thermo Fisher	N/A
Vimentin_Forward GTTGACAATGCGTCTCTGG	Thermo Fisher	N/A
Vimentin_Reverse TGTTCCTGAATCTGAGCCTG	Thermo Fisher	N/A
VEGFR_Forward CACATTGGCCACCATCTGAAC	Thermo Fisher	N/A
VEGFR_Reverse CCATCAGAGGCCCTCCTTG	Thermo Fisher	N/A
PDFGRα_Forward GAGCGCTGACAGTGGCTACAT	Thermo Fisher	N/A
PDFGRα_Reverse TCGTCCTCTCTCTTGATGAAGGT	Thermo Fisher	N/A
PDK1_Forward GGATTGCCCATATCACGTCTTT	Thermo Fisher	N/A
PDK1_Reverse TCCCGTAACCCTCTAGGGAATA	Thermo Fisher	N/A
PDK2_Forward ATGAAAGAG ATCAACCTGCTTCC	Thermo Fisher	N/A
PDK2_Reverse GGCTCTGGACATACCAGCTC	Thermo Fisher	N/A
**Plasmids**		
psPAX2	Addgene	Cat#12260; https://www.addgene.org/12260/
pMD2.G	Addgene	Cat#12259; https://www.addgene.org/12259/
pLENTI_hACE2_PURO	Addgene	Cat#155295; https://www.addgene.org/155295/
**Software and algorithms**		
Fiji ImageJ2 version 2.3.0	NIH	https://imagej.net/software/fiji/
Prism 5	Graphpad Software	https://www.graphpad.com/scientific-software/prism
Image Studio Lite Version 5.2 software	LICOR	https://www.licor.com/bio/image-studio-lite/
FlowJo Software	FlowJo	N/A
Cell Ranger 4.0.0	10x Genomics	https://support.10xgenomics.com/single-cell-gene-expression/software/downloads/latest

(*Continued on next page*)

Continued

REAGENT or RESOURCE	SOURCE	IDENTIFIER
Seurat R package 3.2.1	open source	https://satijalab.org/seurat/
fgsea R package 1.16.0	open source	http://bioconductor.org/packages/release/bioc/html/fgsea.html
enrichR R package v3.0	open source	https://CRAN.R-project.org/package=enrichR

RESOURCE AVAILABILITY

Lead contact

Further information and requests for resources and reagents should be directed to and will be fulfilled by the Lead Contact, Nuria Montserrat (nmontserrat@ibecbarcelona.eu).

Materials availability

All unique organoids generated in this study are available from the Lead Contact with a completed Materials Transfer Agreement.

Data and code availability

- Raw sequencing data for the single cell kidney organoid reported in this paper were deposited in Gene Expression Omnibus (GEO) under the accession number GEO: GSE181002.
- Original code has been deposited at Zenodo and is publicly available at https://doi.org/10.5281/zenodo.6457752.
- All values used to generate the graphs of the paper, original western blot images and additional microscopy images can be found in the file Data S1 that is also available from Mendeley Data at https://doi.org/10.17632/3m6gs9hfsd.1. Any additional information required to reanalyze the data reported in this paper is available from the lead contact upon request.

EXPERIMENTAL MODEL AND SUBJECT DETAILS

Cells

hPSC lines were obtained after the approval of the Ethics Committee from the Clinical Translational Program for Regenerative Medicine in Catalonia (P-CMR [C]) and the Comisión de Seguimiento y Control de la Donación de Células y Tejidos Humanos del Instituto de Salud Carlos III (project numbers: 0336E/1517/2015; 0336E/4166/2020; 0336E/4168/2020; 0336E/1124/2021/ 0336E/2723/2021). hESC ES[4] line and CBiPS1sv-4F-40 iPSC line were obtained from The National Bank of Stem Cells (ISCIII, Madrid). Both hPSC lines were maintained and grown in Essential 8 medium (A1517001, ThermoFisher) in cell culture plates coated with 5 μg/mL vitronectin (A14700, ThermoFisher) with 5% CO2 at 37 °C. Cells were passaged every 4–6 days by disaggregating hPSC colonies into small cell clusters using 0.5 mM EDTA (15575-038, Thermofisher). Vero E6 (ATCC CRL 1586) were maintained in Dulbecco's modified Eagle's medium (DMEM) (Gibco) supplemented with 10% fetal bovine serum (FBS; 10270-106, Gibco) and 1% penicillin/streptomycin (15140122, ThermoFisher). Primary renal proximal tubular epithelial cells were isolated as previously described (Baer and Geiger, 2008; Baer et al., 1997) upon ethics committee of Hospital Clinic de Barcelona approved the procedure (project number: HCB/2021/0119).

Renal proximal tubular epithelial cells were isolated from healthy renal tissues after nephrectomy of renal cell carcinomas. Briefly, prior to cell isolation, the fibrous capsule and the inner medulla were removed and the pieces of tissue (1-mm^2) were digested for 1 h with agitation at 37 °C in Iscove's Modified Dulbecco's Medium containing 1% collagenase IV (Invitrogen). The digested tissue fragments were filtered in 300-, 100-, and 70-μm cell strainers (BD Biosciences), and cell suspensions were overlaid on a pre-cooled Percoll density gradient solution (starting density 1.07 g/ml, Amersham Biosciences) and centrifuged for 40 min at 4 °C at 16,000 rpm. This procedure established a gradient with densities between 1.019 and 1.139 g/ml. The fraction between 1.05 and 1.076 g/ml was collected and washed three times in three volumes of cold Hanks' buffered saline solution (Invitrogen). Finally, renal proximal tubular epithelial cells were plated on plastic plates in proximal tubular cell medium consisting of DMEM without glucose (11966025, Thermofisher) supplemented with 5% FBS (Gibco), 5 mM glucose (Merck), penicillin/streptomycin (15140122, ThermoFisher), ITS 1X (I3146, Merck), and 40 ng/mL human EGF (236-EG-01M, R&D systems) for expansion. Cells were also cultured in REGM medium (CC-3190, Lonza) when indicated.

SARS-CoV-2 isolate

SARS-CoV-2 were isolated on Vero-E6 cells from nasopharyngeal sample of patient diagnosed with COVID-19 in Sweden as described before in Monteil et al. (2020). Alternatively, SARS-CoV-2 (strain BavPat1) was obtained via the European Virology Archive. Virus were amplified in Vero E6 cells and tittered using a plaque assay as previously described (Becker et al., 2008) with fixation of cells 72 hours post infection. Virus was used at a passage 3.

METHOD DETAILS

Generation of genome edited hPSC lines

ACE2 and *BSG* knockout lines were generated using engineered ES[4] and CB[40] PSC lines, in which a doxycycline-inducible Cas9 cassette was introduced at the AAVS1 safe harbor locus (Marco, 2019, 2020). Two crRNAs per gene were designed using the Alt-R Custom Cas9 crRNA Design Tool (IDT) (key resources table). IDT crRNA/tracrRNA duplexes were annealed following manufacturer's guidelines and transfected using Lipofectamine RNAiMAX Transfection Reagent (13778150, Thermofisher) at 10 nM final concentration in 0.25 million doxycycline-treated ES[4] or CB[40] PSCs/mL. Cells were grown 3-5 days after transfection and single-cell seeded at 10, 20, 50, 100 cells/cm^2. Leftover cells were used for DNA purification and further pool editing analysis. Clonal lines were established by manual colony picking (48 clones/crRNA) and further replica-plated. One replica was stored in nitrogen, while the other was used for DNA purification and MiSeq analysis of a locus-specific pooled PCR library generated through two rounds of PCR introducing Illumina adapters and specific indexes labeling each clone (key resources table). Indel frequency in edited pools and allelic composition of clones was obtained by analyzing trimmed Fastq reads using CRIPResso2 webtool (Clement et al., 2019). Analyzed clones were automatically genotyped based on CRISPResso2 results by our in-house CRISPResso2parser.py scripts (available at https://doi.org/10.5281/zenodo.6457752). For each crRNA edit, at least one non-edited clone (+/+), one heterozygous clone (+/frameshift, or +/fs), and one homozygous or trans-heterozygous clone (fs/fs) were amplified, stocked, and used for further experiments.

Kidney organoid differentiation

Prior differentiation (day -1), undifferentiated hPSCs colonies were dissociated into cell clumps using 0.5 mM EDTA (15575-038Thermofisher) at 37°C for 3 minutes. To obtain single cell suspensions samples were then incubated in Accumax (07921, Stem Cell Technologies) at 37°C for 3 additional minutes. Cells were counted using the Countess Automated Cell Counter (Invitrogen) and seeded at 100,000 cells/well on 24 multi-well plates coated with 5 μg/mL vitronectin in the presence of Essential 8 medium (A1517001, Life Technologies) at 37°C overnight. The next day (day 0), the differentiation was initiated by treating monolayer cultures with 8 μM CHIR99021 (CHIR; SML1046, Merck) in Advanced RPMI medium consisting of Advanced RPMI 1640 basal medium (12633020, ThermoFisher) supplemented with 1% Penicillin-Streptomycin and 1% of GlutaMAX (35050061, ThermoFisher) for 3 days with daily medium changes. On day 3, monolayer cultures were treated with 200 ng/mL FGF9 (100-23, Peprotech), 1 μg/mL heparin (H3149, Merck) and 10 ng/mL activin A (Act A) (338-AC-050, Vitro) in Advanced RPMI 1640 basal medium supplemented with 1% Penicillin-Streptomycin and 1% of GlutaMAX for 1 day. On day 4, cell monolayers were treated with 5 μM CHIR, 200ng/mL FGF9 and 1 μg/mL Heparin Advanced RPMI 1640 basal medium (12633020, ThermoFisher) supplemented with 1% Penicillin-Streptomycin and 1% of GlutaMAX for 1 hour. After this 1 hour-treatment cell monolayers were dissociated using TrypLE Express Enzyme (1260402, ThermoFisher) for 1 min collected, and counted. Next, 100,000 cells/well were dispensed on a V-shape 96 multi-well plate (249935, ThermoFisher) and centrifuged at 300g for 5min. Cell spheroids were cultured in Advanced RPMI 1640 basal medium supplemented with 1% Penicillin-Streptomycin and 1% of GlutaMAX, 200ng/mL FGF9 and 1 μg/mL heparin for 7 days with medium changes every other day. From day 11, organoids were maintained in Advanced RPMI 1640 basal medium supplemented with 1% Penicillin-Streptomycin and 1% of GlutaMAX. From that stage medium was changed every other day.

Glucose challenge in kidney organoids

Normoglycemic conditions were emulated exposing kidney organoids to DMEM glucose-free (11966025, ThermoFisher) supplemented with 5 mM D-Glucose (G7021, Merck) changing medium every day until day 23. High oscillatory glucose conditions involved the exposure of day 16 kidney organoids to DMEM glucose-free (11966025, ThermoFisher) supplemented with 5mM D-Glucose (5 mM glucose medium) on even days or with 25mM of D-Glucose (25 mM glucose medium) on odd days until day 23 (5-25mM oscillatory glucose regime). 5 mM glucose medium was additionally supplemented with 20 mM D-Mannitol (M9647, Merck) to obtain the same molarity as in the 25 mM glucose medium.

Isolation of proximal tubular-like cells from kidney organoids

Kidney organoids were stained with fluorescein-conjugated LTL (FL-1321, Vector Laboratories) as described elsewhere (Garreta et al., 2019). Kidney organoids were then dissociated to single cells using Accumax (07921, Stem Cell Technologies) for 15 min followed by 0.25% (wt/vol) trypsin (25300–054, Life Technologies) for 15 min at 37 °C. SA3800 software version 2.0.4 (SONY) was used to acquire flow cytometry samples in the Sony SA3800 spectral cell analyzer (SONY). FACSDiva software version 8.0.1 (BD Biosciences) was used in the FACS Aria Fusion instrument (BD Biosciences) for cell sorting experiments. FlowJo software version 10 was used to analyze the data.

ACE2 mRNA half-life in kidney organoids exposed to 5mM and 5-25 mM glucose conditions

Kidney organoids were treated with 50 μg/ml actinomycin D (Sigma) to inhibit transcription. At the indicated times (0, 4, and 8 hours) total RNA was isolated from the kidney organoids (two pools of 12 kidney organoids per time point) and relative ACE2 mRNA levels were determined by RT-qPCR.

ACE2 lentiviral production and transduction of kidney organoids

Lentiviruses containing full length human ACE2 were produced by polyethylenimine (PEI) transfection of HEK 293T cells (4μg pMDG2, 4μg sPAX, and 8ug lentivirus of interest per well). Corresponding plasmids were purchased from Addgene (see key resources table). Three days post-transfection the lentivirus containing supernatants were collected, filtered with 0.45μm filters and pelleted at 20,000 rpm for 3 hours. The virus pellet was resuspended in 200μL of Optimem, aliquoted and stored at -80 °C. 10^6 lentivirus particles were added to each kidney organoid 3 day prior to infection. Lentiviruses were removed at the time of infection and media containing SARS-CoV-2 or fresh media was added to organoids for 1 hour. Following virus infection, media was removed, organoids were washed 2x and fresh media was added. 24h post-infection organoids were harvested for RNA, protein or fixed in 4% PFA for immunofluorescence.

SARS-CoV-2 infections of kidney organoids and proximal tubular kidney cells

Kidney organoids were infected with 10^6 SARS-CoV-2 infectious particles (as determined in Vero cells) in Advanced RPMI medium (Thermofisher) in a volume of 50μl per well of a 96-well ultra-low attachment plate for 1 hour. Proximal tubular cells were seeded in 24-well plates in REGM medium. One day post-seeding, cells were infected with 10^6 MOI 1 (as determined in Vero cells) SARS-CoV-2 infectious particles in a final volume of 200μl per well in proximal tubular cell medium at 37°C for 1 hour followed by washing with PBS and adding 500μl of new proximal tubular cell medium. At the different days post-infection, samples (organoids or cells) were washed 3 times with PBS and then lysed using Trizol (Thermofisher), RIPA buffer (Thermofisher) or 4% paraformaldehyde (153799, Aname) for ulterior analysis.

SARS-CoV-2 infections of proximal tubular kidney cells exposed to DCA

Proximal tubular cells were seeded in 24-well plates in proximal tubular cell REGM medium. One day post-seeding cells were exposed to 10 mM DCA for 16 hours. After treatments cells were washed with PBS and subsequently infected with SARS-CoV-2 infectious particles (MOI 1) in a final volume of 200μl of REGM medium at 37°C for 1 hour. Cells were washed with PBS and 500μl of new REGM medium was added. At 1 day post-infection, cells were washed 3 times with PBS and then lysed using Trizol (Thermofisher) for further analysis.

Single-cell RNA sequencing

Single cell RNA sequencing was performed as described in our previous study (Monteil et al., 2020). Briefly, kidney organoids were collected and washed twice with PBS, and further dissociated into a single cell suspension by treating them with Accumax (07921, Stem Cell Technologies) for 15 min at 37°C followed by Trypsin-EDTA 0.25% (wt/vol) trypsin (25300-054, Life Technologies) for additional 15 min at 37°C. The reaction was deactivated by adding 10% FBS. The cell suspension was then passed through a 40μm cell strainer. After centrifugation at 1,000 RPM for 5 minutes cell numbers and viability were analyzed using Countess Automated Cell Counter (Invitrogen). Then cell suspensions were loaded onto a well of a 10x Chromium Single Cell instrument (10x Genomics). Barcoding and cDNA synthesis were performed according to the manufacturer's instructions. Qualitative analysis was performed using the Agilent Bioanalyzer High Sensitivity assay. The cDNA libraries were constructed using the 10x Chromium Single cell 3' Library Kit v3.1 according to the manufacturer's original protocol. Libraries were sequenced on either Illumina NovaSeq 6000 or NextSeq 500 2x150 paired-end kits using the following read length: 28bp Read1 for cell barcode and UMI, 8bp I7 index for sample index and either 94bp Read2 in the 5 mM, 11 mM and 5-25 mM oscillatory glucose samples (diabetic samples). For ACE2 WT and ACE 2KO samples (ACE2 samples) 55bp read length was used for Read2. Libraries were pre-processed using Cell Ranger (4.0.0) from 10X Genomics (http://10xgenomics.com). Reads from the ACE2 samples were aligned to the reference human genome (GRch38), whilst diabetic samples were aligned to a custom reference built for this work including the SARS-CoV-2 genome (isolate Wuhan-Hu-1, NC_045512.2). Genome annotation corresponded to Ensembl v93, adding a single gene with a single exon spanning the entire SARS-CoV-2 sequence. The median number of unique molecular identifiers (UMIs) per cell was between 2,300 and 4,600, with a median of 1,300-2,200 genes detected per condition.

Protein extraction and western blot analysis in kidney organoids

Protein was extracted from kidney organoids cultured in 5 mM glucose, 5-25 mM oscillatory glucose or Advance RPMI with or w/o infection using RIPA buffer (ThermoFisher) supplemented with complete protease inhibitor cocktail (ThermoFisher). Samples were centrifuged at 13,000 g for 15 mins at 4°C. Supernatant were collected and protein concentration was measured using a bicinchoninic acid (BCA) protein quantification kit (Thermo Scientific). For western blot analyses, 25 μg of protein were separated in 4-15% Mini-PROTEAN TGX precast gel (BioRad) and blotted onto nitrocellulose membranes. Membranes were blocked at room temperature for 1 hour with TBS 1X-5% BSA. Membranes were then incubated in primary antibody (see key resources table) overnight at 4°C. The membranes were then washed with PBST (PBS 1X + 0.05% Tween20; Merck) for three times 5 minutes and incubated with the secondary antibody (see key resources table). After washing with PBST for 5 minutes twice and with PBS for 5 minutes once, membrane-bound antibodies were detected by fluorescence with the Odyssey Fc Imaging System. Beta-actin (1:5000, Sigma) or Alpha-tubulin (1:5000; Abcam) was used as a loading control for normalization and quantification. Images were analyzed with Image Studio Lite Version 5.2 software.

Histology and Immunocytochemistry

Specimens were fixed at the indicated time points with 4% paraformaldehyde (153799, Aname) overnight at 4°C. Samples were then washed twice with PBS, embedded in paraffin and sectioned into 5 to 10 μm samples. Sections were stained for Hematoxylin and Eosin, Masson's trichrome and Periodic acid–Schiff. Images were captured using the AF7000 Leica microscope.

Immunofluorescence samples were fixed with 4% paraformaldehyde (153799, Aname) for 20 minutes at room temperature, washed trice with PBS and blocked using Tris-buffered saline (TBS) containing 6% donkey serum (S30, Millipore) and 1% Triton X-100 (T8787, Sigma) for 1h at room temperature. Samples were treated overnight at 4°C with the primary antibodies indicated in key resources table diluted in antibody dilution buffer (TBS solution with 6% donkey serum and 0.5% Triton X-100). Samples were then washed three times with antibody dilution buffer and further incubated during 4h at room temperature with fluorescent conjugated secondary antibodies indicated in key resources table. For detection of LTL positive cells (LTL+), samples were stained with biotinylated LTL (B-1325, Vector Labs) using a streptavidin/biotin blocking kit (SP-2002, Vector Labs). LTL+ cells were detected using Alexa Fluor 488 conjugated with streptavidin (SA5488, Vector Labs). Nuclei were stained using 4,6-diamidino-2- phenylindole (DAPI; 1:5000, D1306, Life Technologies) for 30min. Samples were mounted in Fluoromount-G (0100-01, Southern Biotech) and visualized using a Zeiss LSM780 or LSM 880-Airyscan Elyra confocal microscope.

Electron microscopy

Specimens were fixed with 2.5% glutaraldehyde containing 1% tannic acid in 0.1 M phosphate buffer (PB, pH 7.4). Then samples were post-fixed for 1 hour at 4°C with 1% OsO4 in 0.1 M PB. Epoxy resin embedding was performed after graded ethanol series. Then toluidine blue staining was performed in semithin sections using a light microscope. Finally, ultrathin sections were prepared using an EM UC7 ultramicrotome (Leica Microsystems, Mannheim, Germany) and collected on copper grids. Then 4% uranyl acetate and lead citrate were used for staining. Samples were subsequently analyzed with a JEM 1230 electron microscope (JEOL, UK, Ltd).

qRT-PCR

RNA was isolated from cells and kidney organoids using Trizol (Invitrogen). 2 μg RNA was reverse transcribed using the cDNA archival kit (Life Technology), and qRT-PCR was run in the ViiA 7 System (Life Technology) machine using SYBRGreen Master Mix (Applied Biosystem) and gene-specific primers. The data were normalized and analyzed using the ΔΔCt method. The primers sequences used are shown in key resources table.

Oxygen consumption rate (OCR)

The measurement of OCR in kidney organoid-derived cells and proximal tubular cells was performed using an XFe24 extracellular flux analyzer (Seahorse Bioscience) as previously described (Dhillon et al., 2021; Garreta et al., 2019). Cells were plated at density of 20,000 cells/well in a Seahorse cell culture microplate in warm Seahorse XF Assay Medium supplemented with glucose 5mM, pyruvate 0.5mM and glutamine 2mM (Seahorse Bioscience). After 1 h of incubation at 37 °C, plates were loaded into an XF24 respirometry machine (Seahorse Bioscience). Uncoupled and maximum OCR were assayed with oligomycin (10 μM) and FCCP (2 μM). To inhibit complex I- and III-dependent respiration, rotenone (5 μM) and antimycin A (15 μM) were used, respectively. OCR represents the oxygen tension and acidification of the medium as a function of time (pmol min^{-1}). OCR was normalized to protein quantity in each well.

QUANTIFICATION AND STATISTICAL ANALYSIS

scRNA-Seq data analysis

The computational analysis of the resulting UMI count matrices was performed using the R package Seurat (3.2.1) (Stuart et al., 2019). Poor quality cells were removed, such as those in the lower and upper 2.5% quantiles for the number of UMIs and detected genes per cell in diabetic samples and those with < 1,000 detected genes per cell in ACE2 samples. For the latter, the upper thresholds were obtained from visual inspection of the UMI and detected genes distribution. In all cases cells with more than 10% of UMIs assigned to mitochondrial genes were discarded and genes expressed in less than 3 cells were removed from the analyses. Each dataset was subjected to normalization, identification of highly variable features and scaling using the *SCTransform* function, regressing out for the number of detected genes, the % of mitochondrial UMIs and cell cycle. All samples were integrated independently to remove batch effects among them and enable downstream comparisons between different conditions. The infection samples at differentiating conditions (11mM) were processed and integrated in the same way as 5mM and high oscillatory glucose conditions.

Principal component analysis was performed, and the top 20 components were kept for further analysis in the integrated ACE2 dataset. In the diabetic data set the top 40 component were kept for analysis. Clustering was performed by setting the resolution parameter to 0.6 in both cases Dimensional reduction for data visualization was done using the RunUMAP function.

Cell markers in each cluster were identified using the *FindConservedMarkers* and *FindAllMarkers* functions in the non-integrated counts by using the Wilcoxon Rank Sum test. Genes with p value < 0.05 (adjusted by Bonferroni's correction) were retained. Clusters were labelled by comparing the expression of the identified markers with publicly available databases located in KIT (Kidney Interactive Transcriptomics) webpage [http://humphreyslab.com/SingleCell/] and with markers from previous publications (Lindström et al., 2018a, 2018b). Cell type annotations from the diabetic samples were transferred to the 11 mM analysis using the

TransferData function in Seurat. Differential expression analysis to identify changes across conditions were also performed using the Wilcoxon test, keeping as up-regulated genes those present in a fraction of 0.1 of either population, with a log fold-change greater than 0.1 and with an adjusted p value < 0.05. To perform over-representation analysis the R package enrichR (Kuleshov et al., 2016) was used with the database MSigDB_Hallmark_2020''. Gene set enrichment analysis (GSEA) was done with the fgsea R package (Korotkevich et al., 2021), considering the hallmark gene sets from MSigDB.

Image analysis

Quantification of collagen fibers in Masson's trichrome staining of kidney organoids was performed as described elsewhere (Chen et al., 2017) using Fiji ImageJ2 version 2.3.0 software. Briefly, the color deconvolution plugin was used to generate monochromatic images from Masson's trichrome staining images. The integrated intensity of the green component corresponding to the collagen fibers was then calculated using ImageJ.

All raw confocal microscope images were processed with the Fiji ImageJ2 version 2.3.0 software. For optimal image visualization, Z-stacks were projected onto a tiff image where the background signal was removed, and the brightness contrast was enhanced. For fluorescent signal quantification, Z-stacks were projected as a sum of all image pixel grey values across the stack and then selection masks were created at each channel by the SetThreshold tool to measure area, intensity and integrated density of background-free signal. Colocalization was analyzed in Z-stacks using JACoP plugin under Fiji ImageJ2 version 2.3.0 software and calculated as changes of Manders' overlap coefficient.

Data representation and statistical analysis

Student's t test was used to analyze differences between two groups, and One-way or Two-way ANOVA was used to analyze inter-group differences (Tukey's multiple comparisons test). p values less than 0.05 were considered statistically significant. The analysis was performed using GraphPad Prism 5 (GraphPad software). Densitometry results of Western Blots were quantified using the Image Studio Lite Version 5.2 software. All data are presented as mean ± SD and other details such as the number of replicates and the level of significance is mentioned in figure legends and supplementary tables.

Cell Metabolism, Volume *34*

Supplemental information

A diabetic milieu increases ACE2 expression and cellular susceptibility to SARS-CoV-2 infections in human kidney organoids and patient cells

Elena Garreta, Patricia Prado, Megan L. Stanifer, Vanessa Monteil, Andrés Marco, Asier Ullate-Agote, Daniel Moya-Rull, Amaia Vilas-Zornoza, Carolina Tarantino, Juan Pablo Romero, Gustav Jonsson, Roger Oria, Alexandra Leopoldi, Astrid Hagelkruys, Maria Gallo, Federico González, Pere Domingo-Pedrol, Aleix Gavaldà, Carmen Hurtado del Pozo, Omar Hasan Ali, Pedro Ventura-Aguiar, Josep María Campistol, Felipe Prosper, Ali Mirazimi, Steeve Boulant, Josef M. Penninger, and Nuria Montserrat

Figure S1

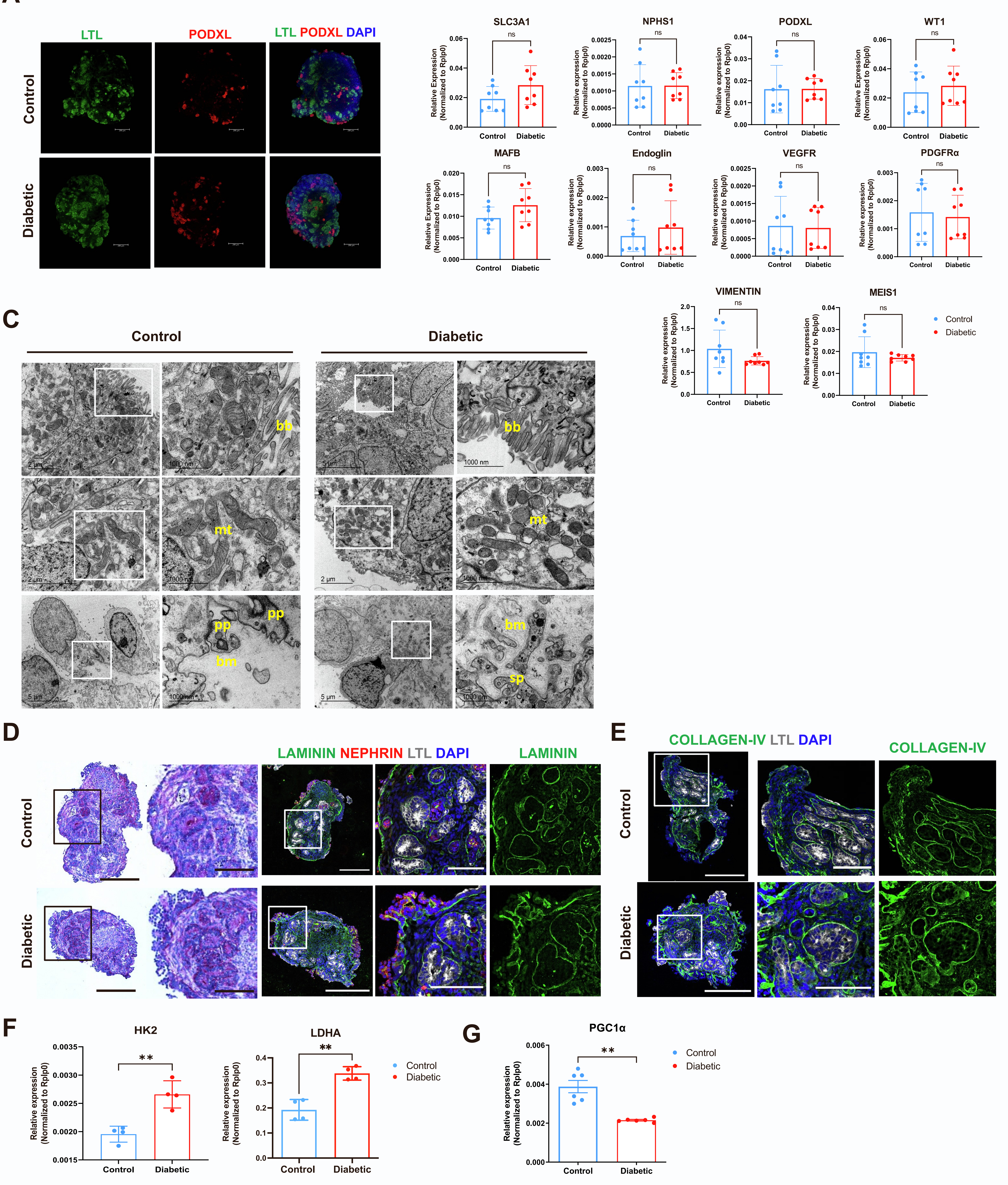

A
LTL
PODXL
LTL PODXL DAPI
Control
Diabetic
B
SLC3A1
NPHS1
PODXL
WT1
MAFB
Endoglin
VEGFR
PDGFRα
VIMENTIN
MEIS1
Relative Expression (Normalized to Rplp0)
ns
Control
Diabetic
C
Control
Diabetic
bb
mt
pp
bm
sp
D
LAMININ NEPHRIN LTL DAPI
LAMININ
E
COLLAGEN-IV LTL DAPI
COLLAGEN-IV
F
HK2
LDHA
G
PGC1α
**

Figure S1. High Oscillatory Glucose Regime Promotes Diabetogenic-like Responses in Human Kidney Organoids, Related to Figure 1.

A) Representative immunofluorescence staining of LTL (green), Podocalyxin (PODXL; red) and DAPI (blue) in kidney organoids cultured under Control or Diabetic conditions for 7 days. Scale bars, 250 μm.

B) mRNA expression level of proximal tubule markers (*SLC3A1*, podocyte markers (*NPHS1*, *PODXL*, *WT1*, *MAFB*), endothelial-like markers (*Endoglin*, *VEGFR*, *PDGFR*α) and stromal-like markers (*MEIS* and *VIMENTIN*) in Control and Diabetic kidney organoids. The data are represented as mean ± s.d. n = 4 independent biological replicates from a pool of 12 organoids/group with at least two technical replicates each; ns, no statistical significance, unpaired Student's *t*-test.

C) TEM in kidney organoids cultured in Control or Diabetic conditions for 7 days. Magnified views of the boxed regions show details of tubular-like epithelial cell-related structures including brush borders (bb) and high mitochondrial (mit) content (upper and middle panels). Bottom panels show podocyte-related structures, including the deposition of a basement membrane (bm), and primary (pp) and secondary cell processes (sp). Scale bars, 2 μm and 5 μm; 1000 nm (magnified views).

D) Representative Periodic Acid-Schiff (PAS) staining in Control and Diabetic kidney organoids. Scale bars, 250 μm, 100 μm (magnified views). Consecutive sections were stained for LAMININ (green), NEPHRIN (red), LTL (grey) and DAPI (blue). Scale bars, 250 μm, 100 μm (magnified views).

E) Representative immunofluorescence staining of COLLAGEN-IV (green) LTL (grey) and DAPI (blue) in Control and Diabetic kidney organoids. Scale bars, 250 μm, 100 μm (magnified views).

F) mRNA expression level of glycolytic enzymes (*HK2* and *LDHA*) in Control and Diabetic kidney organoids. The data are represented as mean ± s.d. n = 2 independent biological replicates per condition with at least two technical replicates each. $^{**}P < 0.01$, unpaired Student's *t*-test.

G) mRNA expression level of *PGC1*α in Control and Diabetic kidney organoids. The data are represented as mean ± s.d. n = 3 independent biological replicates per condition with at least two technical replicates each. ns, no statistical significance, $^{**}P < 0.01$, unpaired Student's *t*-test.

Figure S2

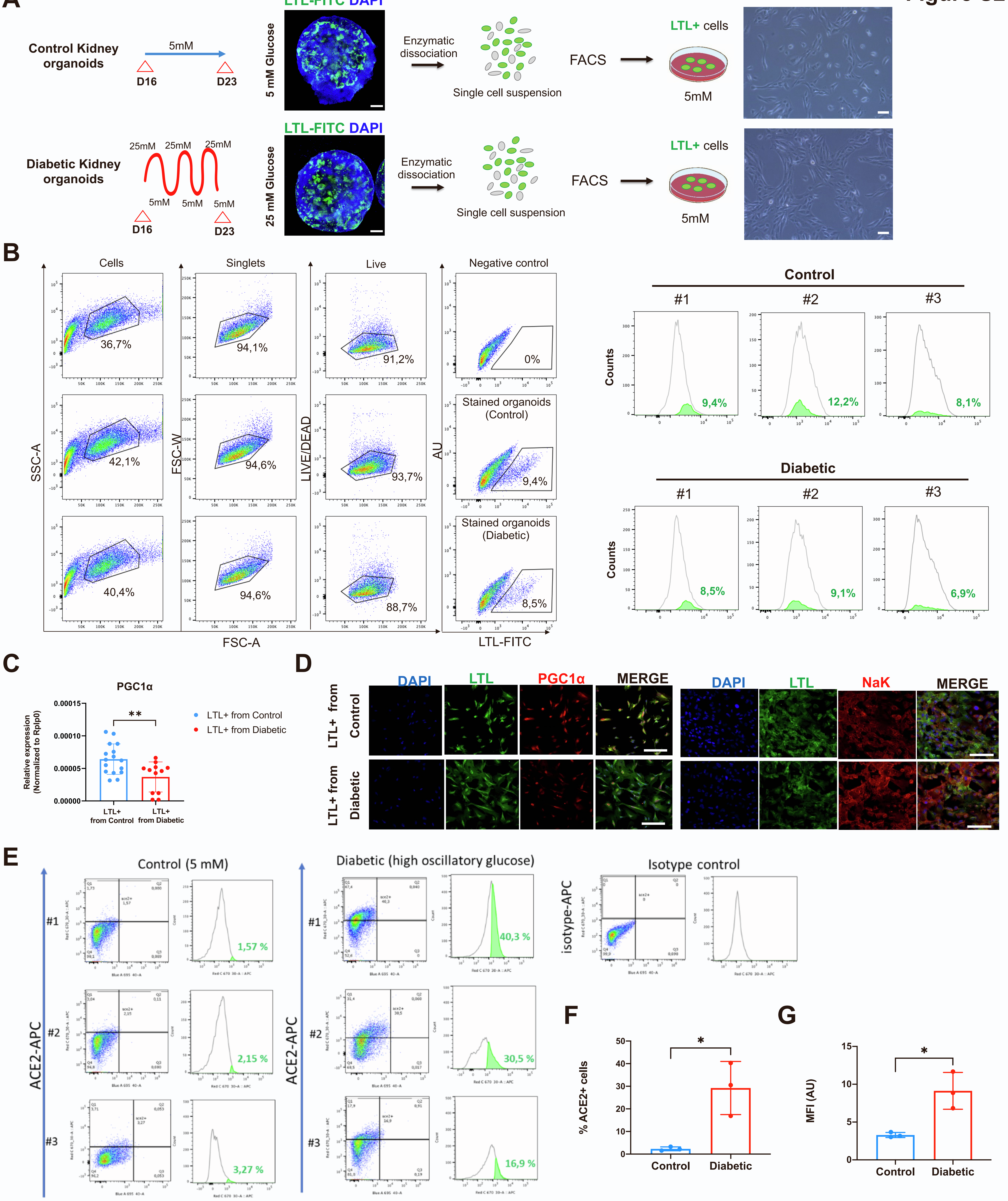

A
Control Kidney organoids
5mM
D16
D23
Diabetic Kidney organoids
25mM 25mM 25mM
5mM 5mM 5mM
LTL-FITC DAPI
5 mM Glucose
25 mM Glucose
Enzymatic dissociation
Single cell suspension
FACS
LTL+ cells
5mM
B
Cells
Singlets
Live
Negative control
Stained organoids (Control)
Stained organoids (Diabetic)
SSC-A
FSC-W
LIVE/DEAD
AU
FSC-A
LTL-FITC
36,7%
94,1%
91,2%
0%
42,1%
94,6%
93,7%
9,4%
40,4%
94,6%
88,7%
8,5%
Control
#1
#2
#3
Counts
9,4%
12,2%
8,1%
Diabetic
8,5%
9,1%
6,9%
C
PGC1α
Relative expression (Normalized to Rplp0)
LTL+ from Control
LTL+ from Diabetic
D
DAPI
LTL
PGC1α
MERGE
NaK
LTL+ from Control
LTL+ from Diabetic
E
Control (5 mM)
Diabetic (high oscillatory glucose)
Isotype control
ACE2-APC
isotype-APC
1,57 %
2,15 %
3,27 %
40,3 %
30,5 %
16,9 %
F
% ACE2+ cells
Control
Diabetic
G
MFI (AU)

Figure S2. Characterization of Tubular Epithelial Cells Derived from Diabetic Human Kidney Organoids Reveals Alterations in Cellular Metabolism, Related to Figure 1 and Figure 2.

A) Kidney organoids exposed to Control or Diabetic conditions were stained with LTL-FITC and dissociated to single cells. Proximal-like epithelial cells (LTL+ cells) were isolated by cell sorting for LTL marker expression and further cultured in 5 mM glucose culture medium. Right panels show representative bright field images. Scale bars, 100 µm.

B) Gating strategy for cell sorting of LTL+ cells from kidney organoids exposed to Control or Diabetic conditions. Numbers in outlined areas indicate percent cells. AU, autofluorescence. Histograms representing the overlay of live cells and LTL+ cells isolated from Control and Diabetic kidney organoids from 3 independent cell sorting experiments.

C) mRNA expression levels of *PGC1*α in LTL+ cells isolated from kidney organoids exposed to Control or Diabetic conditions. The data are presented as mean ± s.d. of at least n = 6 independent biological replicates per condition with two technical replicates each. $^{**}P < 0.01$, unpaired Student's *t*-test.

D) Representative immunofluorescence staining in LTL+ cells isolated from Control or Diabetic kidney organoids and subsequently expanded in 5 mM glucose culture medium for the expression of proximal tubular cell markers including LTL (green), PGC1α (red) and Na-K ATPase (NaK; red). Nuclei were counterstained with DAPI (blue). Scale bars, 100 µm.

E) ACE2 expression in kidney organoids cultured under control or diabetic-like conditions.

F) Representative FACS plots of kidney organoid cells after exposure to control or diabetic-like conditions. Data from 3 independent experiments.

G) Correspondent quantification of the percentage of $ACE2^{+}$ cells in Control and Diabetic organoids in F). Data are mean ± SD of n = 3 independent experiments, unpaired Student's *t*-test.

H) Mean fluorescence intensity of ACE2+ cells in Control or Diabetic conditions. Data are mean ± SD of n = 3 independent experiments, unpaired Student's *t*-test.

Figure S3

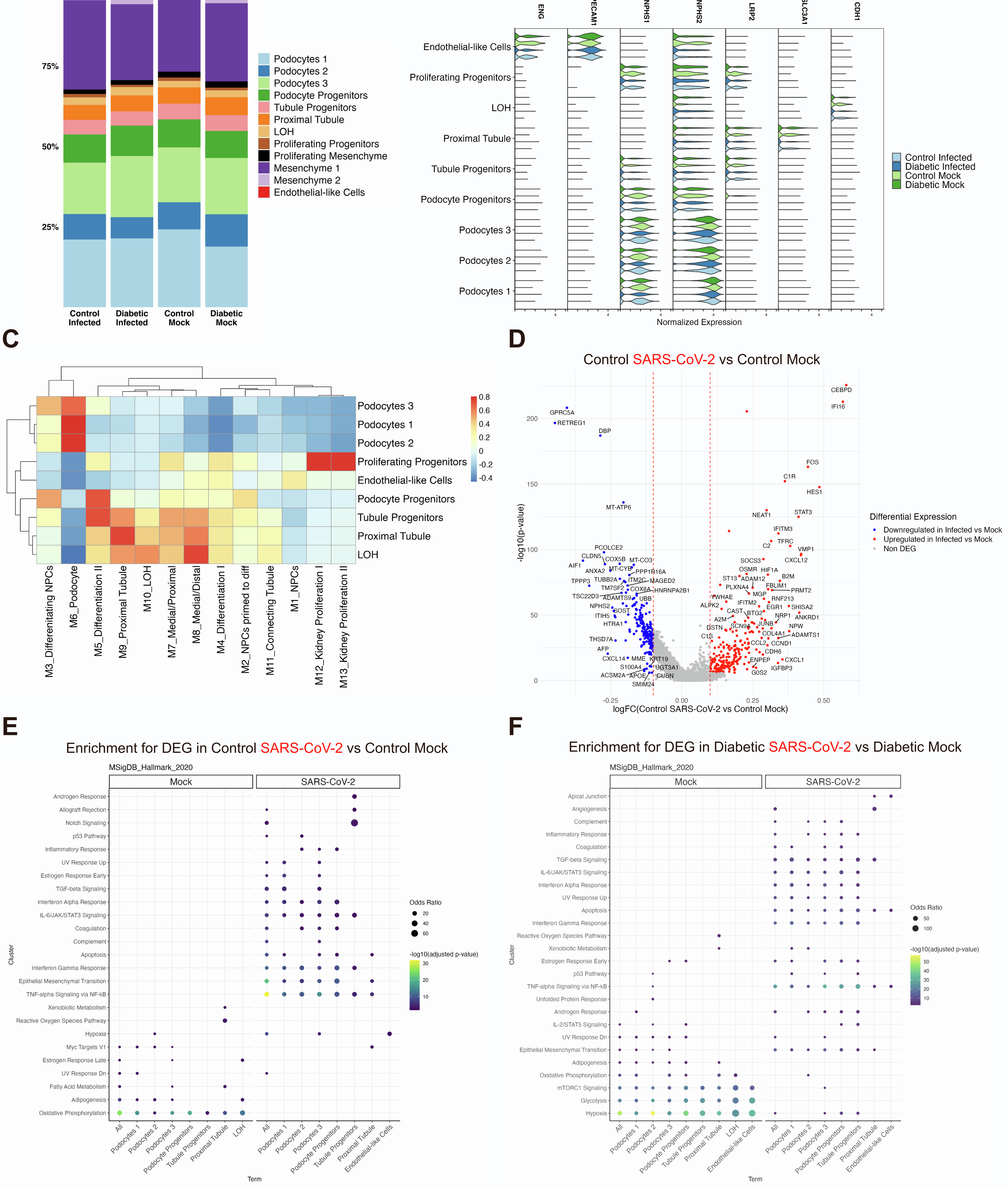

A
B
C
D
E
F
75%
50%
25%
Control Infected
Diabetic Infected
Control Mock
Diabetic Mock
Podocytes 1
Podocytes 2
Podocytes 3
Podocyte Progenitors
Tubule Progenitors
Proximal Tubule
LOH
Proliferating Progenitors
Proliferating Mesenchyme
Mesenchyme 1
Mesenchyme 2
Endothelial-like Cells
ENG
PECAM1
NPHS1
NPHS2
LRP2
SLC3A1
CDH1
Normalized Expression
Control Infected
Diabetic Infected
Control Mock
Diabetic Mock
M3_Differenitating NPCs
M6_Podocyte
M5_Differentiation II
M9_Proximal Tubule
M10_LOH
M7_Medial/Proximal
M8_Medial/Distal
M4_Differentiation I
M2_NPCs primed to diff
M11_Connecting Tubule
M1_NPCs
M12_Kidney Proliferation I
M13_Kidney Proliferation II
Control SARS-CoV-2 vs Control Mock
-log10(p-value)
logFC(Control SARS-CoV-2 vs Control Mock)
Differential Expression
Downregulated in Infected vs Mock
Upregulated in Infected vs Mock
Non DEG
Enrichment for DEG in Control SARS-CoV-2 vs Control Mock
Enrichment for DEG in Diabetic SARS-CoV-2 vs Diabetic Mock
MSigDB_Hallmark_2020
Mock
SARS-CoV-2
Cluster
Term
Odds Ratio
-log10(adjusted p-value)

Figure S3. Phenotypic and Transcriptional Changes in Diabetic Human Kidney Organoids after SARS-CoV-2 Infection, Related to Figure 3.

A) Cell type proportions in Mock or SARS-CoV-2 infected (10^6 virus particles/organoid as determined in Vero cells) kidney organoids exposed to Control or Diabetic conditions after discarding non-renal cells and Proliferating Progenitors 2 clusters.

B) Violin plots for the indicated markers in Mock or SARS-CoV-2 infected (10^6 virus particles/organoid as determined in Vero cells) kidney organoids exposed to Control or Diabetic conditions.

C) Correlation analysis between scores for gene modules from second semester fetal-kidney cell types [1] and scores for gene signatures from the annotated renal-like cell types in Mock and SARS-CoV-2 infected kidney organoids. Scores were obtained with the *AddModuleScore* function from Seurat. Color scale denotes Pearson correlation coefficients.

D) Differentially expressed genes (DEGs) in the comparison of SARS-CoV-2 infected (10^6 virus particles/organoid as determined in Vero cells) against Mock kidney organoids in Control conditions considering only renal-like cell types (Podocytes 1/2/3, PT, LOH, Tubule Progenitors, Podocyte Progenitors, Proliferating Progenitors and Endothelial-like Cells). In the volcano plot, the x-axis indicates log fold change (FC) and the y-axis indicates statistical significance with the –log10(p-value). Genes with an adjusted p-value < 0.05 are considered upregulated (red) if the logFC > 0.1 and downregulated (blue) if the logFC < -0.1. Non DEG are shown in grey.

E) Gene over-representation analysis using the Hallmark database considering DEGs (adjusted p-value < 0.05 and logFC > 0.1) to compare Mock and SARS-CoV-2 infected (10^6 virus particles/organoid as determined in Vero cells) kidney organoids cultured in Control conditions. Each column corresponds to the analysis of DEG upregulated in each condition. Only the ten gene sets with lowest adjusted p-value in each comparison are shown. Circles are coded by color (p-value) and size (Odds ratio).

F) Gene over-representation analysis using the Hallmark database considering DEGs (adjusted p-value < 0.05 and logFC > 0.1) to compare Mock and SARS-CoV-2 infected (10^6 virus particles/organoid as determined in Vero cells) kidney organoids cultured in Diabetic conditions. Each column corresponds to the analysis of DEG upregulated in each condition. Only the ten gene sets with lowest adjusted p-value in each comparison are shown. Circles are coded by color (p-value) and size (Odds ratio).

Figure S4

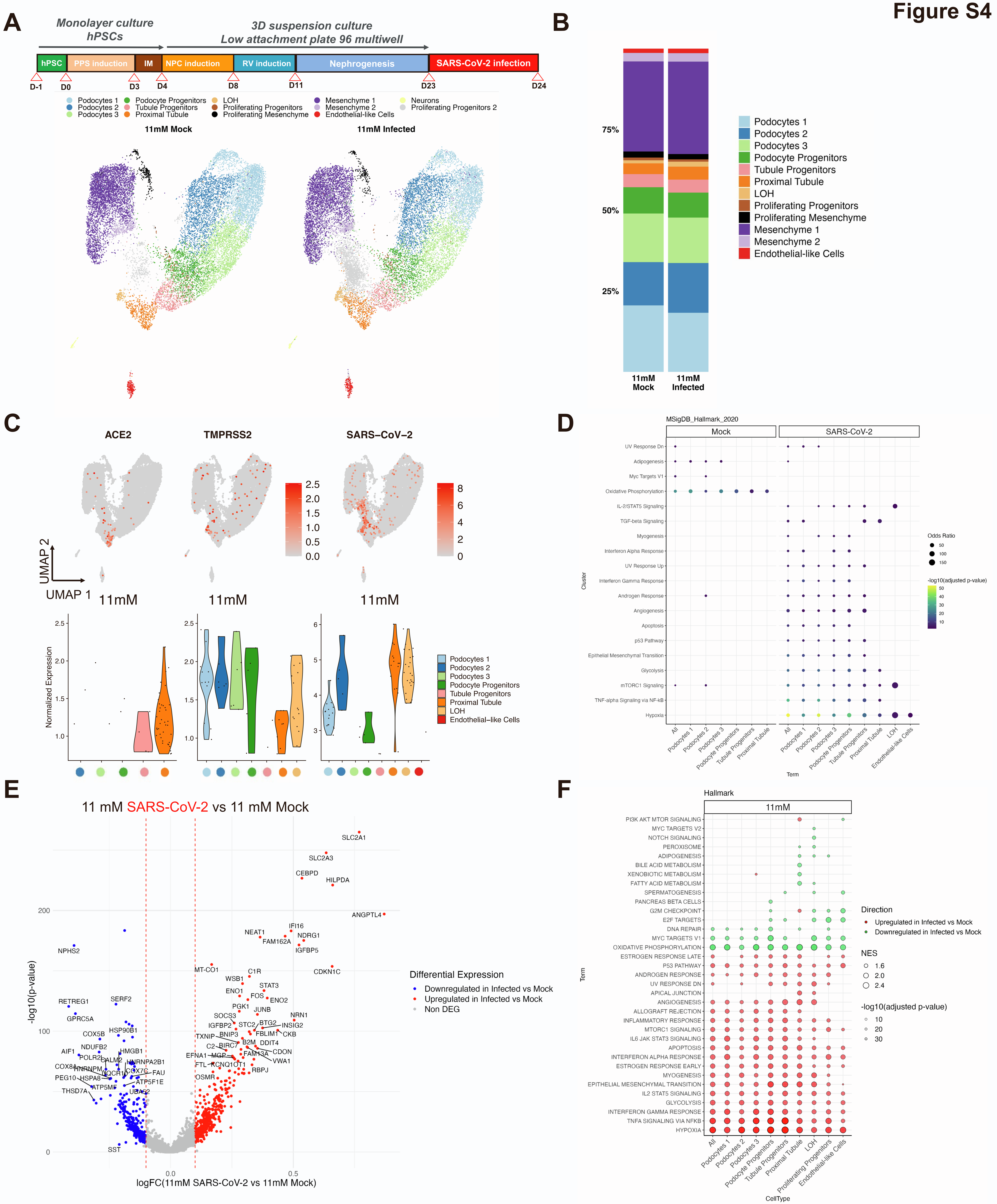

A
Monolayer culture hPSCs
3D suspension culture Low attachment plate 96 multiwell
hPSC
PPS induction
IM
NPC induction
RV induction
Nephrogenesis
SARS-CoV-2 infection
D-1
D0
D3
D4
D8
D11
D23
D24
11mM Mock
11mM Infected
B
11mM Mock
11mM Infected
Podocytes 1
Podocytes 2
Podocytes 3
Podocyte Progenitors
Tubule Progenitors
Proximal Tubule
LOH
Proliferating Progenitors
Proliferating Mesenchyme
Mesenchyme 1
Mesenchyme 2
Endothelial-like Cells
C
ACE2
TMPRSS2
SARS–CoV–2
UMAP 1
UMAP 2
11mM
Normalized Expression
D
MSigDB_Hallmark_2020
Mock
SARS-CoV-2
Odds Ratio
-log10(adjusted p-value)
Cluster
Term
E
11 mM SARS-CoV-2 vs 11 mM Mock
-log10(p-value)
logFC(11mM SARS-CoV-2 vs 11mM Mock)
Differential Expression
Downregulated in Infected vs Mock
Upregulated in Infected vs Mock
Non DEG
F
Hallmark
11mM
Direction
Upregulated in Infected vs Mock
Downregulated in Infected vs Mock
NES
-log10(adjusted p-value)
Term
CellType

Figure S4. Single Cell RNA Sequencing Analysis of SARS-CoV-2 Infected Human Kidney Organoids exposed to 11 mM Glucose Conditions, Related to Figure 3.

A) Schematics for the infection of kidney organoids under 11 mM glucose conditions. Uniform manifold approximation and projection (UMAP) of kidney organoids at 1 dpi with SARS-CoV-2 (10^6 virus particles/organoid as determined in Vero cells). Clusters are colored by cell type annotation.

B) Cell type proportions in the indicated kidney organoid samples after discarding non-renal cells and Proliferating Progenitors 2 clusters.

C) UMAPs for *ACE2, TMPRSS2* and SARS-CoV-2 mRNA expression in kidney organoids exposed to 11 mM glucose at 1dpi. For SARS-CoV-2, expression is considered as undetectable for cells expressing < 5 UMIs. Cells are color-coded based on the expression levels. The violin plots in the bottom panels represent expression level in cells expressing the gene of interest (*ACE2* or *TMPRSS2*) and in cells expressing ≥ 5 UMIs for SARS-CoV-2 mRNA for the indicated cell types.

D) Gene over-representation analysis using the Hallmark database considering DEGs (adjusted p-value < 0.05 and logFC > 0.1) to compare Mock and SARS-CoV-2 infected kidney organoids (10^6 virus particles/organoid as determined in Vero cells) cultured in 11 mM glucose. Each column corresponds to the analysis of DEG upregulated in each condition. Only the ten gene sets with lowest adjusted p-value in each comparison are shown. Circles are coded by color (p-value) and size (Odds ratio).

E) Differentially expressed genes (DEGs) in SARS-CoV-2 infected (10^6 virus particles/organoid as determined in Vero cells) against Mock samples in 11 mM glucose considering only renal-like cell types (Podocytes 1/2/3, PT, LOH, Tubule Progenitors, Podocyte Progenitors, Proliferating Progenitors y Endothelial-like cells). In the volcano plot, the x-axis indicates log fold change (FC) and the y-axis indicates statistical significance with the –log10(p-value Genes with an adjusted p-value < 0.05 are considered upregulated (red) if the logFC > 0.1 and downregulated (blue) if the logFC < -0.1. Non DEG are shown in grey.

F) A hallmark gene set enrichment analysis (GSEA) was performed for kidney organoids cultured in 11 mM glucose comparing SARS-CoV-2 infected (10^6 virus particles/organoid as determined in Vero cells) versus Mock samples. The ten gene sets per direction and sample with lowest adjusted p-value are shown. Each column corresponds to one of the comparisons. Circles are coded by color (direction), size (NES) and transparency (p-value).

Figure S5

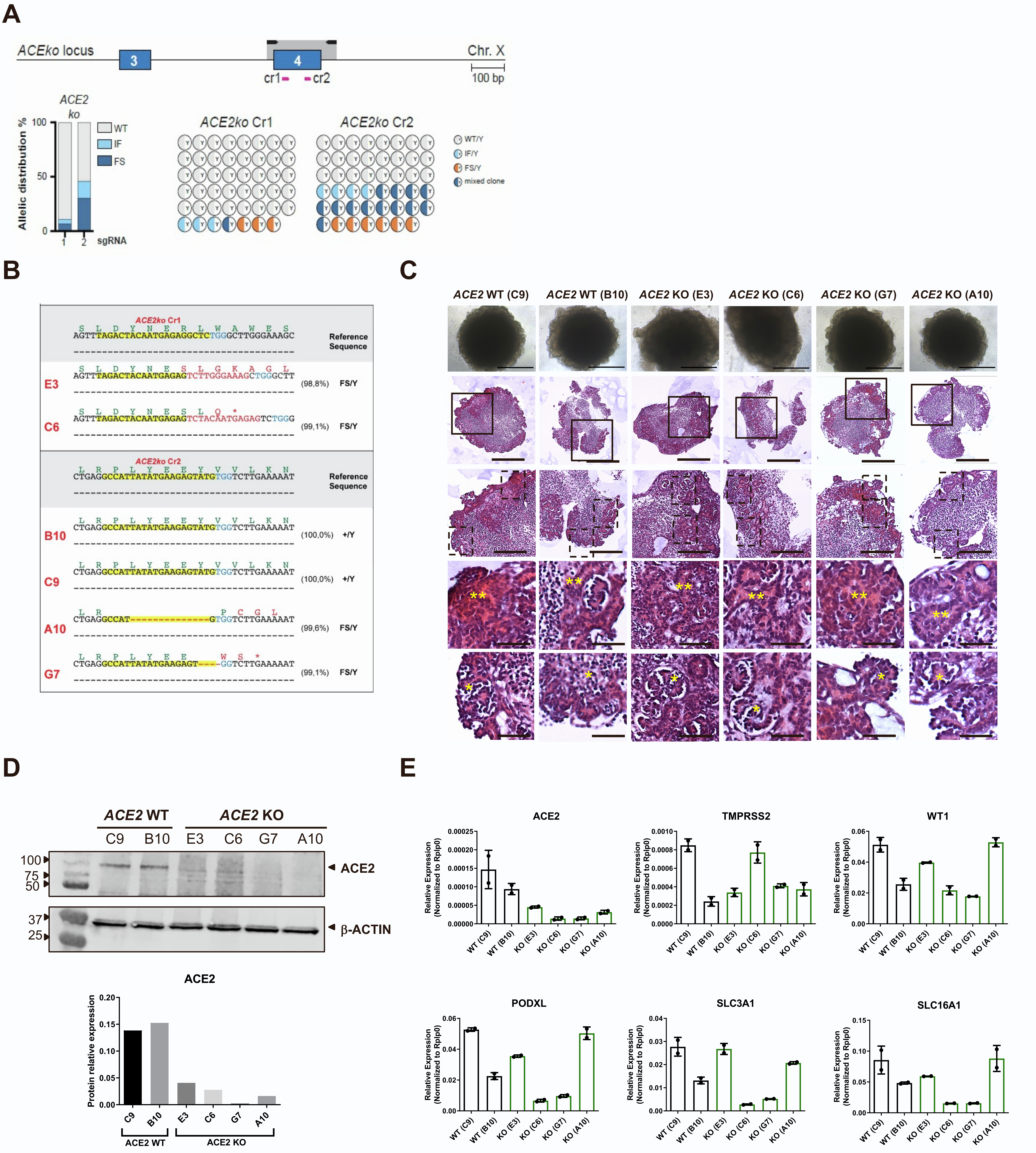
A
ACEko locus
3
4
cr1
cr2
Chr. X
100 bp
ACE2 ko
Allelic distribution %
100
50
0
1
2
sgRNA
WT
IF
FS
ACE2ko Cr1
ACE2ko Cr2
WT/Y
IF/Y
FS/Y
mixed clone
B
ACE2ko Cr1
Reference Sequence
E3
(98,8%) FS/Y
C6
(99,1%) FS/Y
ACE2ko Cr2
Reference Sequence
B10
(100,0%) +/Y
C9
(100,0%) +/Y
A10
(99,6%) FS/Y
G7
(99,1%) FS/Y
C
ACE2 WT (C9)
ACE2 WT (B10)
ACE2 KO (E3)
ACE2 KO (C6)
ACE2 KO (G7)
ACE2 KO (A10)
D
ACE2 WT
ACE2 KO
C9
B10
E3
C6
G7
A10
100
75
50
37
25
ACE2
β-ACTIN
ACE2
Protein relative expression
0.20
0.15
0.10
0.05
0.00
ACE2 WT
ACE2 KO
E
ACE2
TMPRSS2
WT1
PODXL
SLC3A1
SLC16A1
Relative Expression (Normalized to Rplp0)
WT (C9)
WT (B10)
KO (E3)
KO (C6)
KO (G7)
KO (A10)

Figure S5. Generation and Infection of *ACE2* KO Human Kidney Organoids, Related to Figure 4.

A) Schematic of Cas9/gRNA-targeting sites (pink arrows) in *ACE2 locus* showing exon structure (blue boxes) and PCR amplicons (light gray boxes in all figures in this study). Histogram shows allelic sequence distribution after the transfection of the different gRNAs in undifferentiated human pluripotent stem cells expressing an inducible Cas9 (iCas9). wt, wild-type; mut, mutation; FS, frameshift.

B) Representative sequence of the wild type (+Y) or *ACE2* mutant clones generated with the different gRNAs.

C) Representative phase contrast images of *ACE2* WT and *ACE2* KO kidney organoids. Scale bars, 250 µm. Same specimens were analyzed by Hematoxylin and Eosin staining showing tubular-like (**) and glomerular-like (*) structures. Scale bars, Scale bars, 250 µm, 100 µm (magnified views); 50 µm (magnified views from dashed line boxes).

D) Protein levels of ACE2 in the generated clones as determined by Western Blot. β-ACTIN was used as loading control. Quantification of changes in ACE2 protein expression is shown. The data are represented as mean ± s.d. *n* = 1 independent experiment from a pool of 12 organoids/group.

E) mRNA expression level of *ACE2* and *TMPRSS2*, podocyte markers (*WT1* and *PODXL*) and proximal tubular markers (*SLC3A1* and *SLC16A1*) in *ACE2* WT and *ACE2* KO kidney organoids. The data are represented as mean ± s.d. *n* = 1 independent experiment from a pool of 12 organoids/group with at least two technical replicates each.

Figure S6

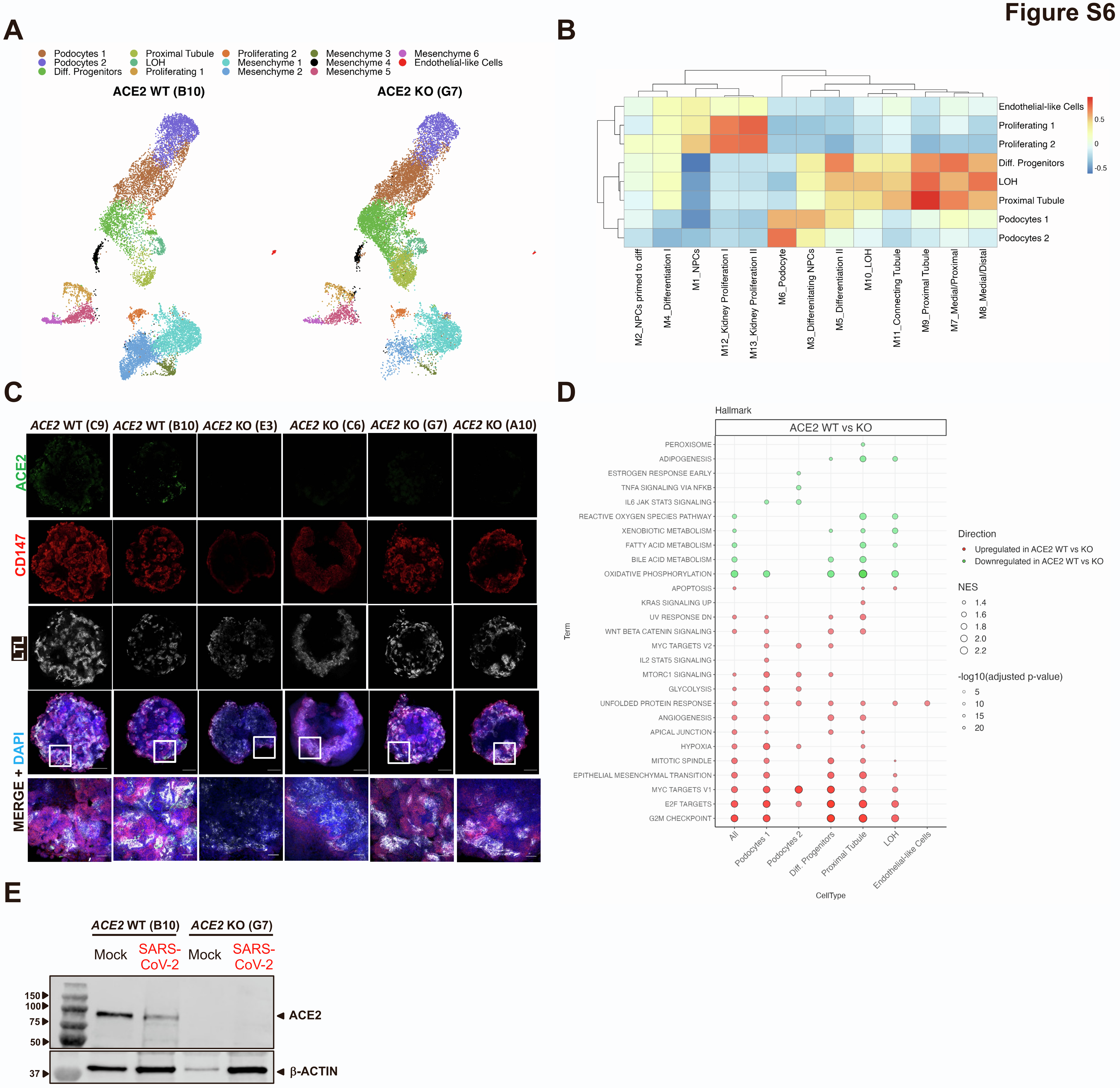
A
Podocytes 1
Podocytes 2
Diff. Progenitors
Proximal Tubule
LOH
Proliferating 1
Proliferating 2
Mesenchyme 1
Mesenchyme 2
Mesenchyme 3
Mesenchyme 4
Mesenchyme 5
Mesenchyme 6
Endothelial-like Cells
ACE2 WT (B10)
ACE2 KO (G7)
B
Endothelial-like Cells
Proliferating 1
Proliferating 2
Diff. Progenitors
LOH
Proximal Tubule
Podocytes 1
Podocytes 2
0.5
0
-0.5
M2_NPCs primed to diff
M4_Differentiation I
M1_NPCs
M12_Kidney Proliferation I
M13_Kidney Proliferation II
M6_Podocyte
M3_Differenitating NPCs
M5_Differentiation II
M10_LOH
M11_Connecting Tubule
M9_Proximal Tubule
M7_Medial/Proximal
M8_Medial/Distal
C
ACE2 WT (C9)
ACE2 WT (B10)
ACE2 KO (E3)
ACE2 KO (C6)
ACE2 KO (G7)
ACE2 KO (A10)
ACE2
CD147
LTL
MERGE + DAPI
D
Hallmark
ACE2 WT vs KO
PEROXISOME
ADIPOGENESIS
ESTROGEN RESPONSE EARLY
TNFA SIGNALING VIA NFKB
IL6 JAK STAT3 SIGNALING
REACTIVE OXYGEN SPECIES PATHWAY
XENOBIOTIC METABOLISM
FATTY ACID METABOLISM
BILE ACID METABOLISM
OXIDATIVE PHOSPHORYLATION
APOPTOSIS
KRAS SIGNALING UP
UV RESPONSE DN
WNT BETA CATENIN SIGNALING
MYC TARGETS V2
IL2 STAT5 SIGNALING
MTORC1 SIGNALING
GLYCOLYSIS
UNFOLDED PROTEIN RESPONSE
ANGIOGENESIS
APICAL JUNCTION
HYPOXIA
MITOTIC SPINDLE
EPITHELIAL MESENCHYMAL TRANSITION
MYC TARGETS V1
E2F TARGETS
G2M CHECKPOINT
Term
All
Podocytes 1
Podocytes 2
Diff. Progenitors
Proximal Tubule
LOH
Endothelial-like Cells
CellType
Direction
Upregulated in ACE2 WT vs KO
Downregulated in ACE2 WT vs KO
NES
1.4
1.6
1.8
2.0
2.2
-log10(adjusted p-value)
5
10
15
20
E
ACE2 WT (B10)
ACE2 KO (G7)
Mock
SARS-CoV-2
Mock
SARS-CoV-2
150
100
75
50
37
ACE2
β-ACTIN

Figure S6. SARS-CoV-2 Infection in *ACE2* KO Human Kidney Organoids, Related to Figure 4.

A) UMAP of *ACE2* WT kidney organoids (generated from clone B10) and *ACE2* KO kidney organoids (generated from *ACE2* KO clone G7) colored by annotated cell types.

B) Correlation analysis between scores for gene modules from second semester fetal-kidney cell types and scores for gene signatures from the annotated renal-like cell types in *ACE2* WT and *ACE2* KO kidney organoids. Scores were obtained with the *AddModuleScore* function from Seurat. Color scale denotes Pearson correlation coefficients.

C) Immunofluorescence staining for ACE2 (green), CD147 (red), LTL (grey) and DAPI (blue) in *ACE2* WT and *ACE2* KO kidney organoids. Scale bars, 250 μm, 50 μm (magnified views).

D) Hallmark gene set enrichment analysis (GSEA) was performed in kidney organoids generated from *ACE2* WT and *ACE2* KO lines. The ten gene sets per direction and sample with lowest adjusted p-value are shown. Circles are coded by color (direction), size (NES) and transparency (p-value).

E) Protein levels of ACE2 in Mock treated and SARS-CoV-2 infected (10^6 virus particles/organoid as determined in Vero cells) *ACE2* WT and *ACE2* KO kidney organoids are shown by Western Blot analysis. β-ACTIN was used as loading control. Data from a pool of 12 organoids/group is shown.

Figure S7

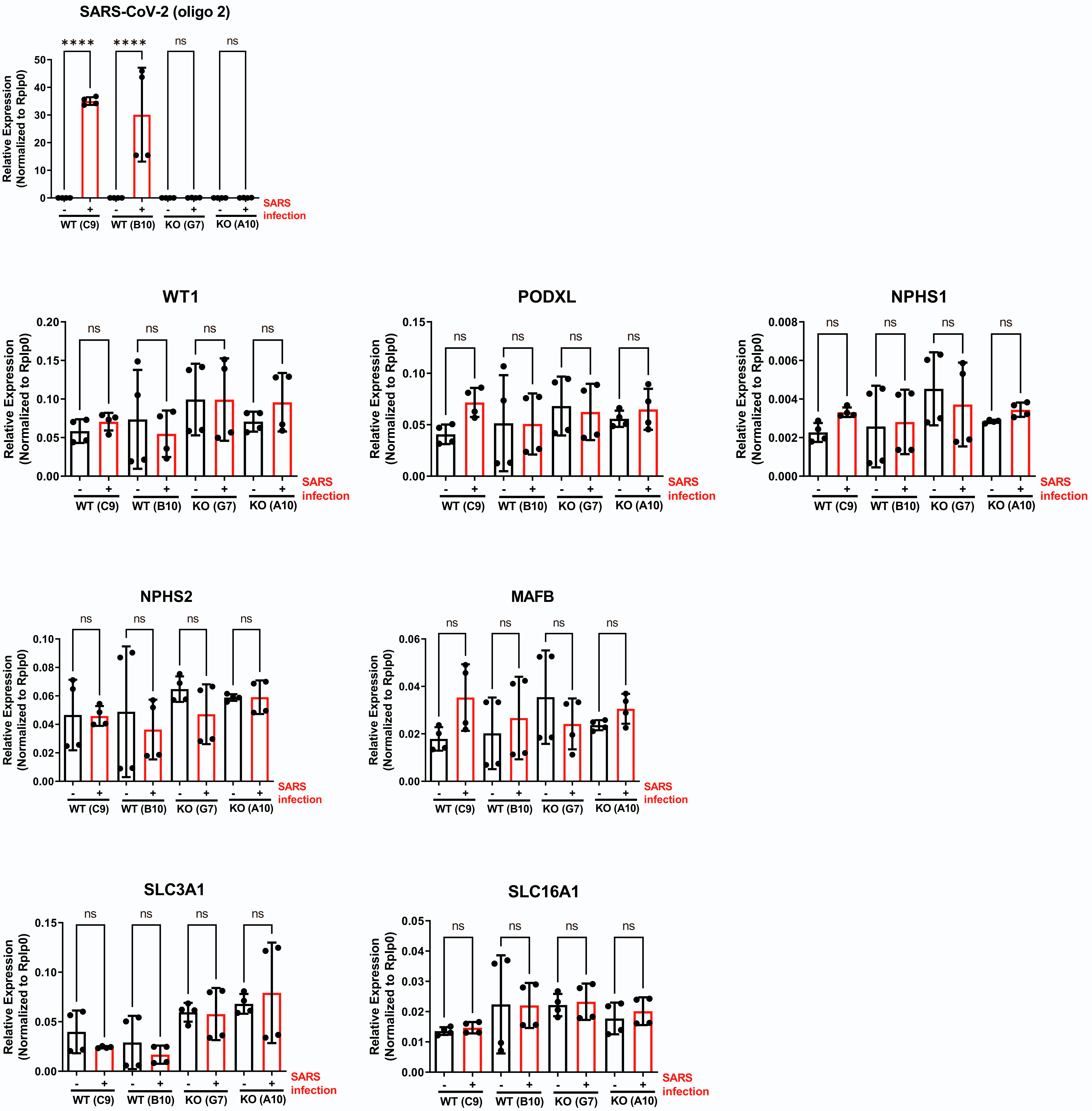
SARS-CoV-2 (oligo 2)
****
****
ns
ns
Relative Expression (Normalized to Rplp0)
0
10
20
30
40
50
SARS infection
- +
WT (C9)
WT (B10)
KO (G7)
KO (A10)
WT1
0.00
0.05
0.10
0.15
0.20
PODXL
0.00
0.05
0.10
0.15
NPHS1
0.000
0.002
0.004
0.006
0.008
NPHS2
0.00
0.02
0.04
0.06
0.08
0.10
MAFB
0.00
0.02
0.04
0.06
SLC3A1
0.00
0.05
0.10
0.15
SLC16A1
0.00
0.01
0.02
0.03
0.04
0.05

Figure S7. SARS-CoV-2 Infection in *ACE2* KO Human Kidney Organoids, Related to Figure 4.

Quantitative real time analysis was performed at 3 dpi in Mock or SARS-CoV-2 infected (10^6 virus particles/organoid as determined in Vero cells) *ACE2* WT and *ACE2* KO kidney organoids to quantify the mRNA expression levels of SARS-CoV-2, podocyte markers (*WT1*, *PODXL*, *NPHS1*, *NPHS2*, *MAFB*), and proximal tubular markers (*SLC3A1* and *SLC16A1*). Data from a pool of 12 organoids/group is shown. The data are represented as mean ± s.d. $n = 2$ independent experimental replicates from a pool of 12 organoids/group with at least two technical replicates each. ****$P < 0.0001$, ns, no statistical significance, One-way ANOVA, Tukey's multiple comparisons test.

Figure S8

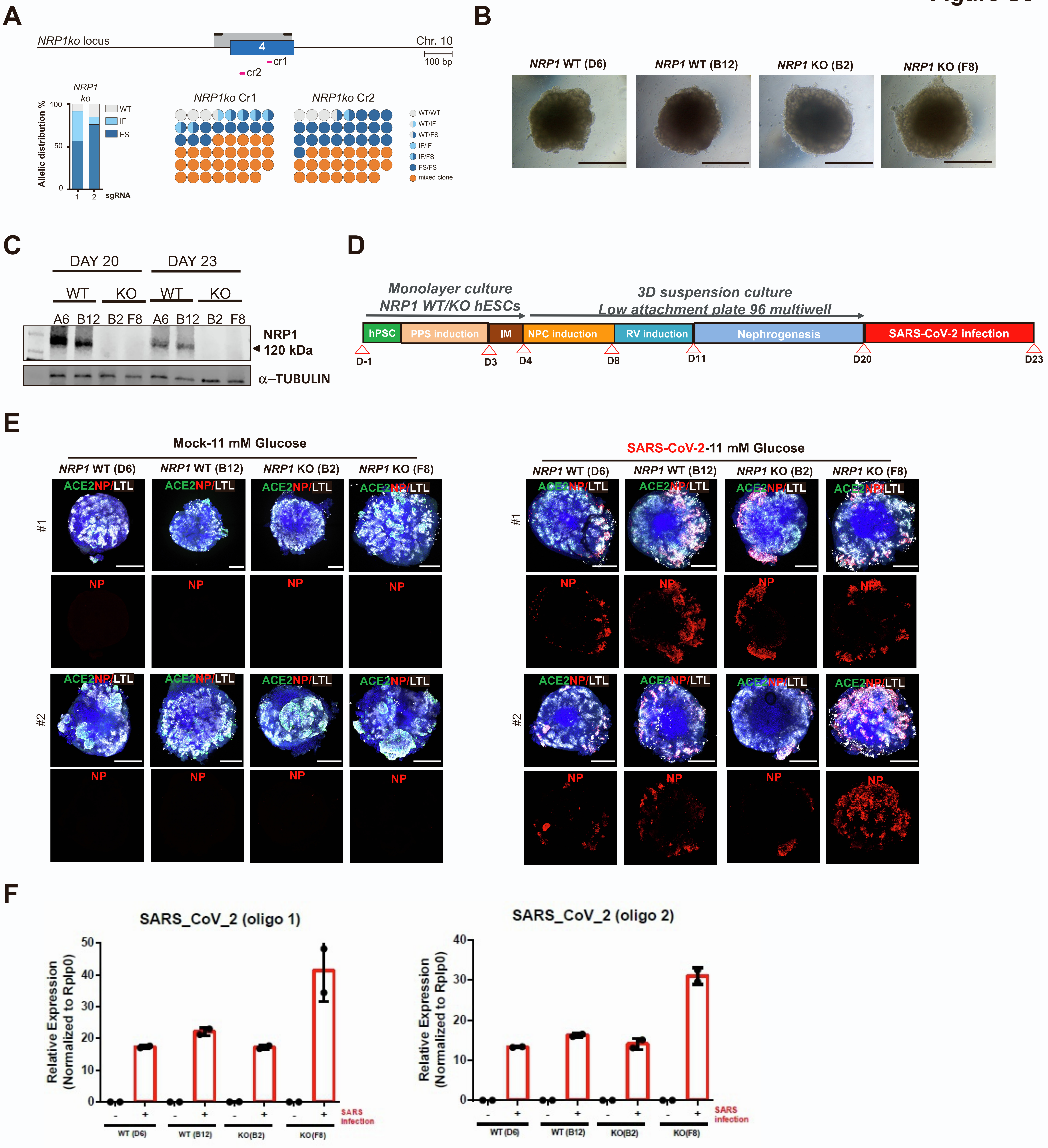
A
NRP1ko locus
4
Chr. 10
100 bp
cr1
cr2
NRP1 ko
Allelic distribution %
WT
IF
FS
sgRNA
NRP1ko Cr1
NRP1ko Cr2
WT/WT
WT/IF
WT/FS
IF/IF
IF/FS
FS/FS
mixed clone
B
NRP1 WT (D6)
NRP1 WT (B12)
NRP1 KO (B2)
NRP1 KO (F8)
C
DAY 20
DAY 23
WT
KO
A6 B12 B2 F8 A6 B12 B2 F8
NRP1
120 kDa
α–TUBULIN
D
Monolayer culture
NRP1 WT/KO hESCs
3D suspension culture
Low attachment plate 96 multiwell
hPSC
PPS induction
IM
NPC induction
RV induction
Nephrogenesis
SARS-CoV-2 infection
D-1
D3
D4
D8
D11
D20
D23
E
Mock-11 mM Glucose
SARS-CoV-2-11 mM Glucose
ACE2NP/LTL
NP
#1
#2
F
SARS_CoV_2 (oligo 1)
SARS_CoV_2 (oligo 2)
Relative Expression (Normalized to Rplp0)
SARS infection
WT (D6)
WT (B12)
KO(B2)
KO(F8)

Figure S8. Generation and Infection of *NRP1* KO Human Kidney Organoids, Related to Figure 4 and Figure 5.

A) Schematic of Cas9/gRNA-targeting sites (pink arrows) in the *NRP1 locus* showing exon structure (blue boxes) and PCR amplicons (light gray boxes). Histogram shows allelic sequence distribution after the transfection of the different gRNAs in undifferentiated ES[4] cells expressing an inducible Cas9 (iCas9). wt, wild-type; mut, mutation; FS, frameshift.

B) Representative bright field images of *NRP1* WT and *NRP1* KO kidney organoids. Scale bars, 250 μm.

C) Protein levels of NRP1 in *NRP1* WT and *NRP1* KO kidney organoids are shown by Western blot analysis. αTUBULIN was used as loading control. Data from a pool of 12 organoids/group is shown.

D) Experimental scheme for the generation and viral infection of *NRP1* WT and *NRP1* KO kidney organoids.

E) Immunofluorescence was performed at 3 dpi in Mock treated or SARS-CoV-2 infected (10^6 virus particles/organoid as determined in Vero cells) *NRP1* WT and *NRP1* KO kidney organoids. Representative confocal images show the detection of ACE2 (green), viral nuclear protein (NP, red), LTL (grey) and DAPI (blue). Scale bars, 250 μm.

F) Quantitative real time analysis was performed at 3 dpi in Mock treated or SARS-CoV-2 infected (10^6 virus particles/organoid as determined in Vero cells) *NRP1* WT and *NRP1* KO specimens for the detection of SARS-CoV-2 mRNA expression levels. The data are represented as mean ± s.d. n = 1 independent experiment from a pool of 12 organoids/group with at least two technical replicates each.

Figure S9

A

	Non Diabetic	Diabetic
**Age (yr)***	58.0 ± 23.07	71.3 ± 5.86
**Sex, F/M**	1/2	0/3
**Diabetic onset**	N/A	Type 2

*** Mean ± SD**

B

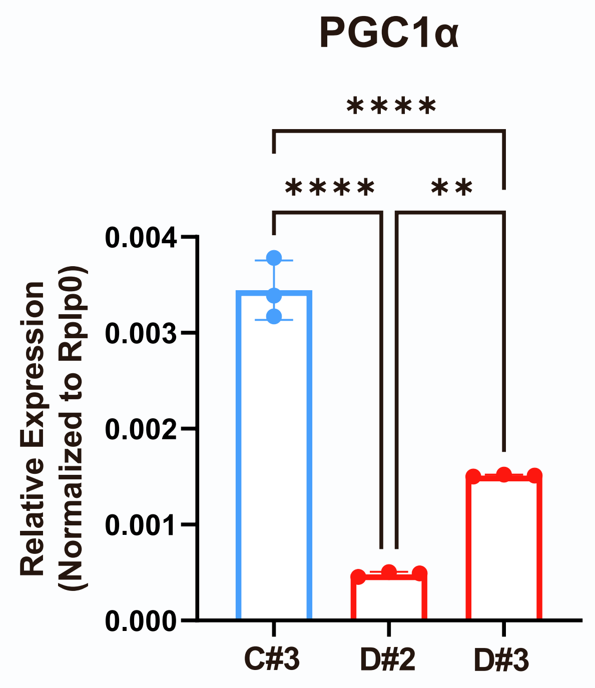

C

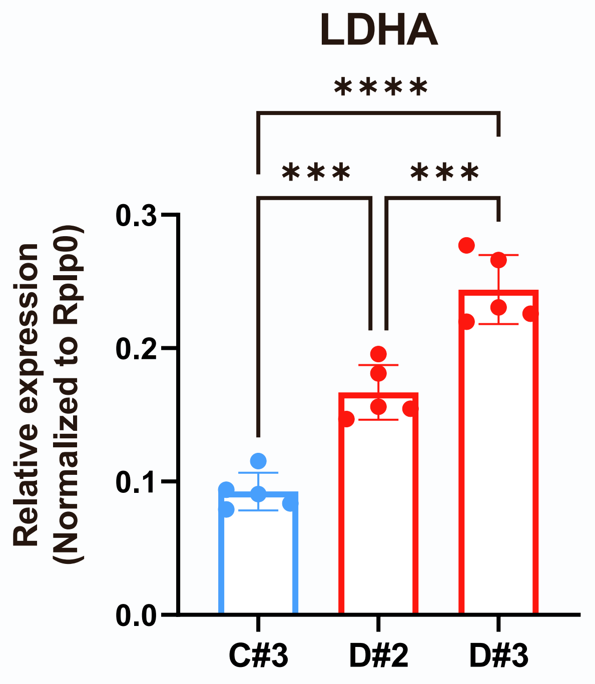

D

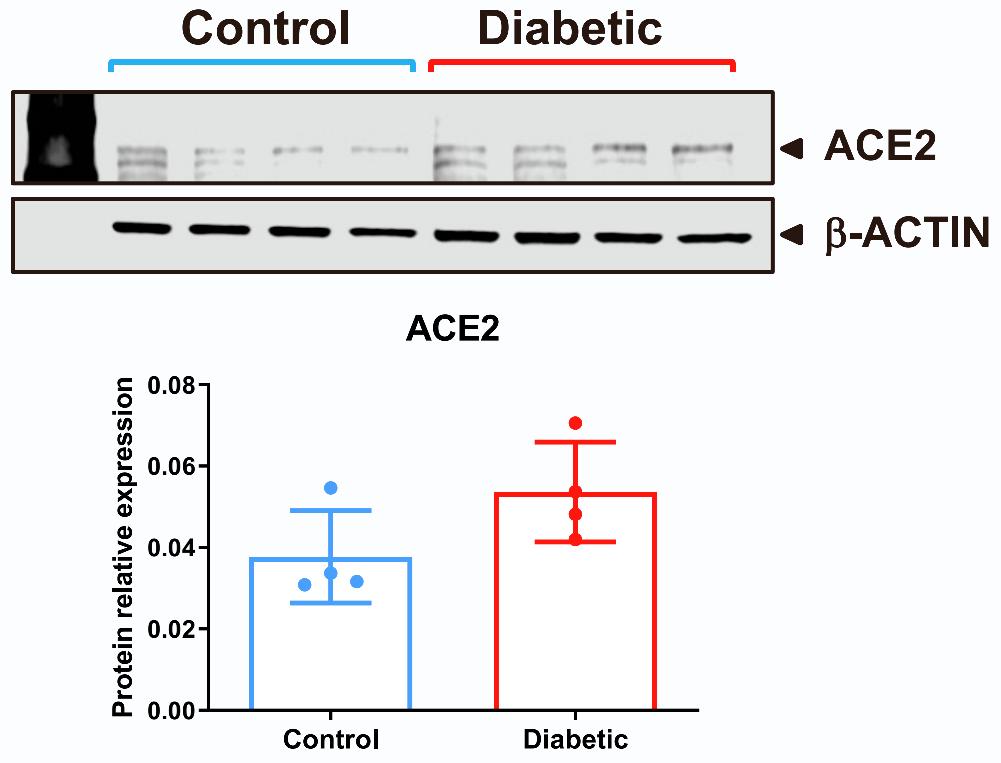

E

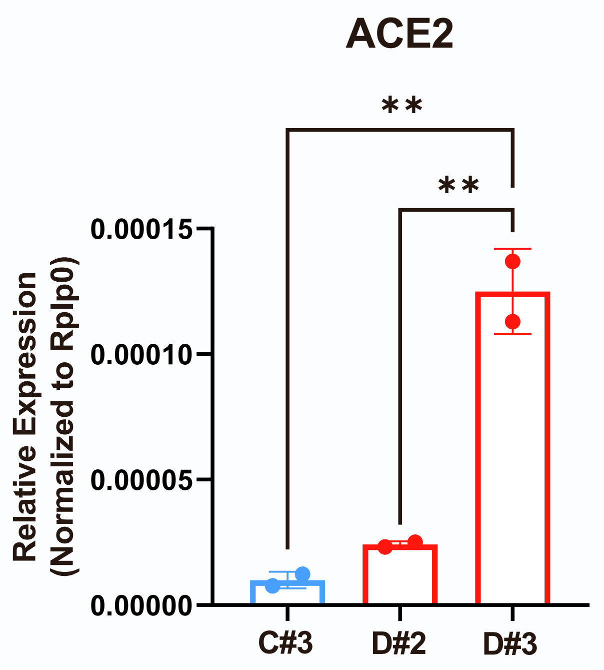

F

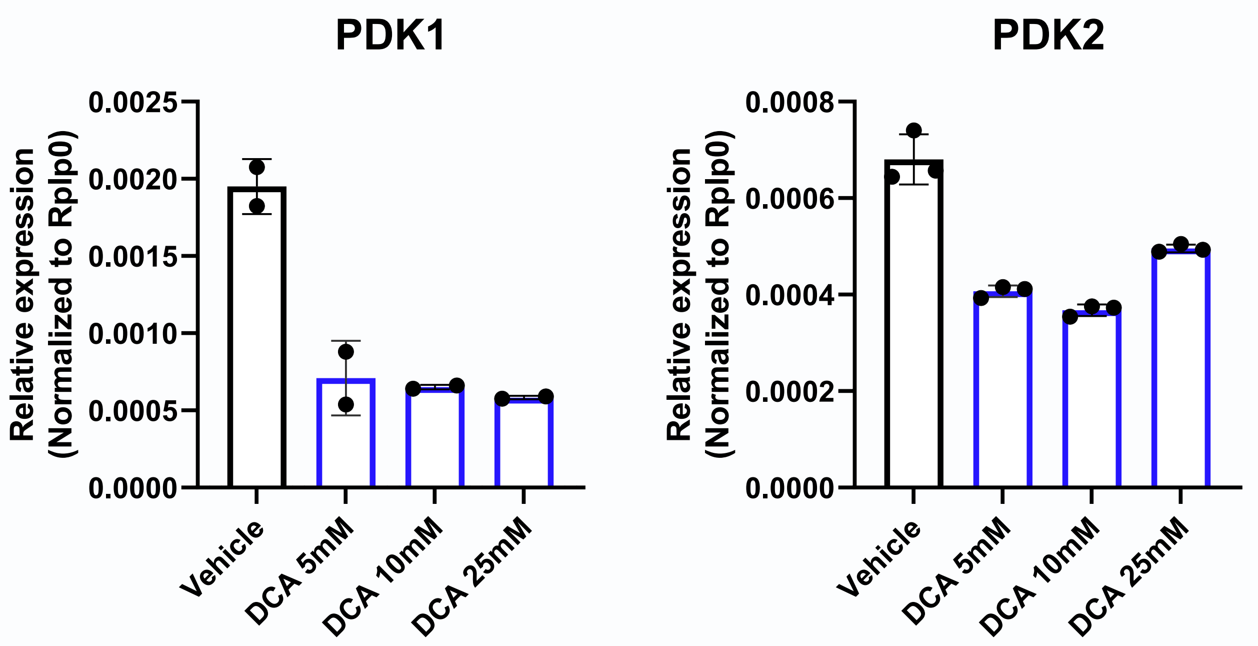

Figure S9. SARS-CoV-2 Infection in Patient Kidney Cells, Related to Figure 7.

A) Characteristics of non-diabetic (control) and diabetic patients, from which primary human proximal tubular cells (HPTCs) were isolated and used in this study.

B) mRNA expression levels of *PGC1*α in Control and Diabetic HPTCs determined by qPCR. The data are presented as mean ± s.d. of at least n = 1 independent biological replicate per condition with three technical replicates each. **P < 0.005, ****P < 0.0001, One-way ANOVA, Tukey's multiple comparisons test.

C) mRNA expression levels of *LDHA* by qPCR in Control and Diabetic HPTCs. The data are presented as mean ± s.d. of at least n = 1 independent biological replicate per condition with five technical replicates each. ***P < 0.001, ****P < 0.0001, One-way ANOVA, Tukey's multiple comparisons test.

D) Protein levels of ACE2 in HPTCs from Control and Diabetic donors as determined by Western blotting. β-ACTIN was used as loading control. The data are presented as mean ± s.d. of n = 2 independent biological replicates per condition with two technical replicates each.

E) mRNA expression levels of *ACE2* by qPCR in Control and Diabetic HPTCs. The data are presented as mean ± s.d. of at least n = 1 independent experiment with two technical replicates each. **P < 0.005, One-way ANOVA, Tukey's multiple comparisons test.

F) mRNA expression levels of *PDK1* and *PDK2* in HPTCs after treatment with different doses of dichloroacetate (DCA) or vehicle, determined by qPCR. The data are presented as mean ± s.d. of n = 1 independent experiment with at least two technical replicates each.
